# Supplementary material for: Socio-ecological factors influencing dietary behaviours among adolescents and young adults in rural Eastern Uganda: A qualitative study
Source: PLoS One. 2025 Dec 2;20(12):e0337797. doi: 10.1371/journal.pone.0337797 (PMC12671741; doi:10.1371/journal.pone.0337797)
Supplement: S1 File — Conceptual framework illustrating the socio-ecological determinants of dietary behaviors among adolescents and young adults in rural Mayuge, Eastern Uganda. (DOCX) [file pone.0337797.s001.docx]

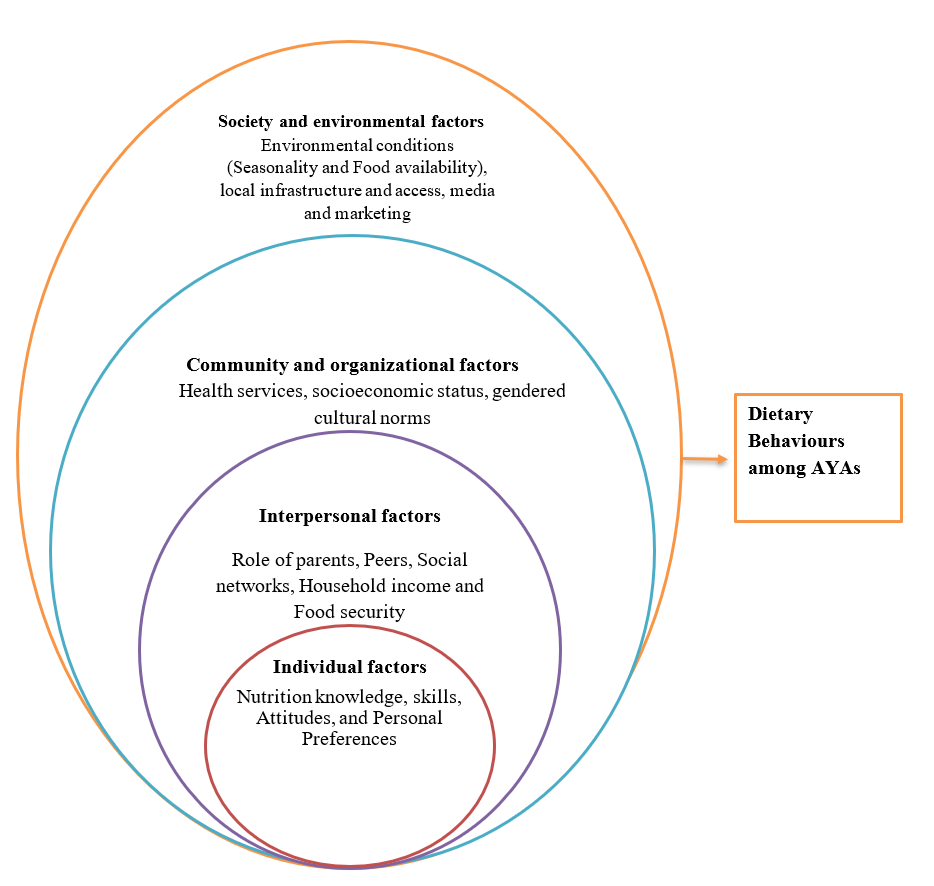


***Fig 1:*** *A hypothesized conceptual framework showing socio-ecological factors influencing dietary behaviours among AYAs in Rural Eastern Uganda*
