# Supplementary material for: Socio-ecological factors influencing dietary behaviours among adolescents and young adults in rural Eastern Uganda: A qualitative study
Source: PLoS One. 2025 Dec 2;20(12):e0337797. doi: 10.1371/journal.pone.0337797 (PMC12671741; doi:10.1371/journal.pone.0337797)
Supplement: S2 File — Interview and focus group discussion guides used with adolescents, parents, food vendors, teachers, CSO staff, and district officials. (DOCX) [file pone.0337797.s002.docx]

**ARISE-NUTRINT: Adolescents and young adults focus group discussion guide**

1. **Welcome**
   1. Facilitator welcomes participants on arrival.
   2. Facilitator invites participants to sit in a circle and sign the sign-in sheet with gender, age, and grade.
2. **Introduction**

*Good morning/afternoon/evening and welcome to our session. Thank you for taking the time to talk to us about issues relating to health and nutrition among young people in (country name). My name is ________ and assisting me today in taking notes is __________. The focus group we are conducting together is part of our effort to understand how health behaviors among adolescents in (country name) can be improved. We are meeting with you to learn about your ideas of a healthy diet and lifestyle and what can be done to help young people in achieving those. We will use this information to advocate for programs and policies that help you and students like you to live healthier lives.*

1. **Assent**

*Before we begin our discussion, I want to emphasize that the things you say during this focus group will not be attached to your name. We will be recording the focus groups so that we can make sure to capture the thoughts, opinions, and ideas we hear from the group. What you say may be put in a summary of this discussion, but here will be no way to identify who said what, and your names will not be included in the summary report.*

*You can refuse to answer any question or withdraw from the study at any time. We understand how important it is that this information is kept private and confidential. We ask participants to respect each other’s confidentiality and not to identify who participated in this group to others. If you have any questions now or after the discussion, you can always contact a study team member like me, or you can call the (country name) project team leaders whose names and phone numbers are on this form.*

**Distribute the assent forms.**

1. **Focus Group Format**
   1. Facilitator acknowledges that some participants may know each other, and others do not.
   2. Facilitator asks the group if anyone has participated in a focus group before. Facilitator explains that focus groups are being used more and more frequently for research.
   3. Facilitator emphasizes that the participants are the experts: we learn from you.
   4. Facilitator explains that we are not trying to get everyone to agree or achieve consensus, rather, we’re gathering information. It is okay if you have different opinions and ideas than others in the group. It is our goal to develop programs and policies that better support your nutrition and health. We want them to meet your needs and preferences.
   5. Facilitator clarifies that in this project, we are doing both questionnaires and focus group discussions. The reason for using both of these tools is that we can get more in-depth information from a smaller group of people in focus groups. This allows us to understand the context behind the answers given in the written survey and helps us to explore topics in more detail than we can do in a written survey.
   6. Focus group will last about one hour. Feel free to move around. If you need to use the restroom or excuse yourself at any time, please feel free to do so.
2. **Ground Rules**

Facilitator gives the ground rules:

- 1. *There are no right or wrong answers to the questions being asked today; all ideas are good ideas..*
  2. *We welcome and respect different points of view. Please answer what you truly think, regardless of the opinions of the other group members.*
  3. *We want to hear the opinion of everyone in the room, and we may ask some questions directly to you. But if you are uncomfortable with a question, feel free to pass. You are under no obligation to answer any question that makes you feel uncomfortable in any way.*
  4. *Only one person should talk at a time. When you are talking, please make sure to speak clearly.*
  5. *As mentioned earlier, information provided in the focus group must be kept confidential.*
  6. *Please turn off all cell phones and other recording devices you might have with you.*
  7. *During today’s discussion, there are many things to talk about; at times we may need to stop the discussion in order to move on. I apologize in advance for that. But we are distributing note cards to you, 1) so that if you have something to share that we don’t get time to cover, you can write it down, and 2) if you have something you want to share privately and not with the other participants, then you can write down those thoughts also. We will collect your pads at the end of the discussion. Please don’t put your names on them.*

**Turn on Tape Recorder**

Facilitator asks if there are any questions before we get started and address those questions.

1. **Focus Group Questions**

**1. To begin with, I want to know what being healthy means to you.**

What comes to your mind when you hear the word ‘healthy’?

1. What has shaped your understanding of being healthy?
2. **Now I want to know more about your dietary habits.**
3. What does a healthy diet mean to you?
4. What factors influence your food and drink choices?
5. What motivates you to eat a healthy diet?
6. What challenges do you face when it comes to eating a healthy diet?

**Let us discuss your surroundings when it comes to food.** What is your opinion on the:

1. dietary habits of young people in your household and community?
2. What food and drinks do vendors in your community sell?
3. What food and drinks do you typically buy from food vendors?
4. What factors influence you to purchase food and drinks sold by food vendors?
5. What are your opinions, as well as those of young people in your household or community, about food and drinks sold by food vendors?
6. **Now I will ask you about nutrition related health programmes**
7. Have you ever received any nutrition or food related assistance in school or community?
8. How have these programmes influenced your dietary or lifestyle behaviours?
9. What kind of programs would help young people like you to have a healthier diet and lifestyle? (Probe: Two nutrition/dietary based programmes)

Discuss for each program; the scope, at whom it should target, by whom and how it should be delivered, which challenges are to be expected and how they could be overcome.

Thank you again for your help. We really, really appreciate your time and your knowledge.

**ARISE-NUTRINT parents or guardians and teachers focus group discussion guide**

Let me begin our discussion by reviewing a few things about the focus group. We will be focusing on some specific topics. We are interested in what everyone has to say about them. If someone throws out an idea that you want to expand on, or if you have a different point of view, please speak up. Sometimes I may have to interrupt the discussion to bring us back to the topic or to move on to another question or topic, to make sure that we cover everything on our agenda.

**We will follow several practical guidelines during this session**:

- We want everyone to express your opinions about the discussion topics. We are interested in different points of view. There are no right or wrong answers, and we are not here to resolve any issues you may bring up or to reach agreement. We just want to understand your views.
- Please feel free to agree or disagree with what other people say, while respecting their views.
- Please do not hold side conversations. We want to be able to hear from everyone, and to be able to hear what everyone says.
- Please wait to be recognized by the moderator before speaking.
- Sometimes we will go around the table to share views on a topic. You can always “pass” if you prefer not to comment on that particular topic.
- Because we are also audio-recording the session, it would really help us if you could speak up.
- Do you have any questions so far?

**1.** To begin with, let us talk about the food habits and cooking practices in your household and community.

1. What are the norms and values related to cooking and dietary habits in this community?
2. What constitutes healthy eating habits in your and your family’s daily life?
3. What challenges do you and your family face when it comes to eating a healthy diet?
4. What motivators do you and your family have when it comes to eating a healthy diet?

2. Now we would like to discuss about the eating habits of young people in this community.

1. What do young people typically prefer to eat on a daily basis?
2. What is your opinion of the eating habits of young people in your household and community?
3. Today’s young people also prefer to drink sweet and sugary beverages. What do you think about that?
4. How does the presence of food vendors influence the dietary habits of young people?
5. What role do social institutions such as family or schools play in influencing the dietary habits of young people? 3. Let us discuss about some of the barriers and challenges to eating healthy among young people.
   1. What are the most significant barriers to young people eating healthy? [Probe about: taste and craving, food prices, availability of healthy and diverse food items, availability of junk food, time, and cooking practices]
   2. What can be done to promote healthy eating among young people? [For the suggested option, ask the following probe]
   3. How should this intervention be designed?
   4. Who should be involved in designing this intervention?
   5. What challenges do you anticipate and how should they be addressed?
   6. We need to add a table to capture the probes a-c

**Closing Remarks**

Thank you very much for participating in this focus group. The information you have provided has been very helpful. It will be used to help us to design interventions to promote healthy eating habits among young people. Are there any questions that I can answer before we end the session?

**ARISE-NUTRINT: Food Vendors Interview Guide 2024**

Hello, my name is (*insert name*) and I am a researcher with (*insert institution name*). I am part of a study team examining young people’s nutrition and health in this country and others. I would like to ask your opinions about young people’s eating habits and food choices. I would also like to ask you about the types of food you sell and the prices you sell them for. The information you provide will help inform our understanding of the foods that are accessible and available to young people in this community.

1. Can you tell me what healthy eating means to you? What are the reasons you think eating healthy is important?
2. What do you consider to be healthy food items? Which foods do you consider unhealthy? Which food items do you consider to be junk food?
3. What is your opinion of the eating habits of young people in this community? How healthy or unhealthy are their eating habits? Could you please explain why you think so?
4. What are the major health concerns associated with young people eating unhealthy food?
5. How do young people in this community feel about healthy eating and diets?
6. How do young people make food choices? How do boys and girls differ in their food choices?
7. What are the most preferred food items by young people when they visit your shop? What could be the reasons for the selection of these food items?
8. Today’s young people also prefer to drink sweet and sugary beverages like juices and soda. What is your observation about it in this community? What are the most preferred drinks by young people?
9. What sources of nutrition and healthy eating knowledge do young people have?
10. What role models do young people look up to as they learn about healthy eating and nutrition? In what ways do their role models influence their eating habits?
11. In what ways do community norms and cultural beliefs influence young people's food habits?

**Closing Remarks**

Thank you very much for participating in this interview. The information you have provided has been very helpful. It will be used to help us to design interventions to promote healthy eating habits among young people. Are there any questions that I can answer before we end this interview?

**ARISE-NUTRINT: Local Government and Civil Society Representatives) Interview Guideline 2024**

Hello, my name is (insert name) and I am a researcher with (insert institution name). I am part of a study team examining young people’s nutrition and health in this country and others. I would like to ask your opinions about young people’s eating habits and food choices. I would also like to ask you about the barriers to healthy diet among adolescents, policies and programs promoting heathy eating, as well as proposed improvements. The information you provide will help inform our understanding of the foods that are accessible and available to young people, food policies and implementation of the same in this community.

1. What are the major concerns regarding nutrition and diet among adolescents in [Country]?
2. What determinants shape up the dietary choices of adolescents in [Country]? [Note: Probe on all the following determinants]
   1. Biological determinants such as hunger, appetite and taste
   2. Economic determinants such as costs of food items and income
   3. Physical determinants such as access, education, skills, and time
   4. Social determinants such as class, cultural beliefs, and social context
   5. Psychological determinants such as mood, stress, etc.
   6. Attitude, beliefs and knowledge about food
3. On what information do you believe adolescents are basing their food choices? Do you see any problems arising from this?
4. What do you perceive to be the biggest barriers to healthy diets among adolescents in [country]? Please think of individual barriers but also of barriers in their immediate environment (their physical environment as well as their families and peers) and barriers on a societal level.
   1. Can you think of any ways to overcome these barriers?
5. Which role do you think does the availability or lack thereof of certain food options in adolescents’ environments play in encouraging or discouraging a healthy diet?
6. What kinds of programs are implemented at national or sub-national level to promote healthy eating habits and improve adolescent nutrition? (e.g., Aids, feeding programs, cash-transfers, supplements, education) [Note: Ask the following probes for each program that is being currently implemented?]
   1. What is the main objective of [Name of the program]?
   2. What kinds of activities are conducted under [Name of the program]?
   3. Who are the primary beneficiaries of [Name of the program]? Who are the secondary beneficiaries?
   4. Who is delivering them?
   5. How frequently are they delivered?
   6. What is the current coverage of this program?
7. How do you perceive the utility of these programs and policies? [Note: Ask these questions for each program and policy that is being implemented]
   1. How does [Name of the program] target the right populations and address their needs?
   2. How are the targeted populations made aware of [Name of the program]? What are the different ways for the target population to participate in [Name of the program] and avail the services and how to participate?
   3. What kinds of outcomes are achieved by the program?
   4. In your opinion, has [Name of the program] been successful in achieving the desired outcomes? What are the reasons for it?
   5. What are the unintended consequences of [Name of the program]?
   6. What are the challenges in implementation of [Name of the program]?
8. Can you think of any programs that could complement or replace the already existent ones?
   1. At whom should they be aimed? Why?
   2. Who should deliver them? Why?
   3. How frequently should they be delivered?
   4. What kind of resources would be needed to implement them?
   5. What challenges might be encountered during implementation?
   6. How could these challenges be addressed? [Note: Probe for each outcome]

Thank you very much for taking out time to talk to us and share your insights and opinions.
