# Supplementary material for: Socio-ecological factors influencing dietary behaviours among adolescents and young adults in rural Eastern Uganda: A qualitative study
Source: PLoS One. 2025 Dec 2;20(12):e0337797. doi: 10.1371/journal.pone.0337797 (PMC12671741; doi:10.1371/journal.pone.0337797)
Supplement: S3 File — Completed COnsolidated criteria for REporting Qualitative research (COREQ) checklist for this study. (DOCX) [file pone.0337797.s003.docx]

**Completed COREQ checklist**

**Manuscript title:** Socio-ecological Factors Influencing Dietary Behaviours among Adolescents and Young Adults in Rural Eastern Uganda: A Qualitative Study

| **No. Item** | **Guide questions/description** | **Reported on line number..** |
| --- | --- | --- |
| **Domain 1: Research team and reflexivity** |  |  |
| Personal Characteristics |  |  |
| 1. Inter viewer/facilitator | Which author/s conducted the interview or focus group? | Included in data collection; line no. 163 – 168 and 176 - 177 |
| 2. Credentials | What were the researcher’s credentials? (E.g.  PhD, MD) | Included in data collection; line no. 163 – 168 |
| 3. Occupation | What was their occupation at the time of the study? | Included in data collection; line no. 163 – 168 |
| 4. Gender | Was the researcher male or female? | Included in data collection; line no. 163 – 168 and 176 - 177 |
| 5. Experience and training | What experience or training did the researcher have? | Included in data collection; line no. 163 – 168 and 176 - 177 |
| Relationship with participants |  |  |
| 6. Relationship established | Was a relationship established prior to study commencement? | Included in data collection; line no. 185 – 186 |
| 7. Participant knowledge of the interviewer | What did the participants know about the researcher? (e.g. personal goals, reasons for doing the research). | Included in data collection; line no. 190– 193 |
| 8. Interviewer characteristics | What characteristics were reported about the interviewer/facilitator? (e.g. Bias, assumptions, reasons and interests in the research topic) | Included in data collection; line no. 163 – 169,184 – 187, and 190– 193 |
| **Domain 2: Study design** |  |  |
| Theoretical framework |  |  |
| 9. Methodological orientation and Theory | What methodological orientation was stated to underpin the study? (e.g. grounded theory, discourse analysis, ethnography, phenomenology, content analysis). | Included; line no. 96 – 98, 134 – 137, and 210 – 213 |

| Participant selection |  |  |
| --- | --- | --- |
| 10. Sampling | How were participants selected? (e.g. purposive, convenience, consecutive, snowball) | Included; line no. 114 - 125 |
| 11. Method of approach | How were participants approached? (e.g. face-to-face, telephone, mail, email) | Included; line no. 170 - 171 and 173 |
| 12. Sample size | How many participants were in the study? | Included; line no. 234 and table 1 |
| 13. Non-participation | How many people refused to participate or dropped out? Reasons? | Included; line no. 124 - 125 |
| Setting |  |  |
| 14. Setting of data collection | Where was the data collected? (e.g. home, clinic, workplace) | Included; line no. 170 – 172 and 177 - 180 |
| 15. Presence of nonparticipants | Was anyone else present besides the participants and researchers? | Included; line no. 170 – 172 and 177 - 178 |
| 16. Description of sample | What are the important characteristics of the sample? (e.g. demographic data, date) | Included; line no. 111 – 125, and in Table 1 |
| Data collection |  |  |
| 17. Interview guide | Were questions, prompts, guides provided by the authors? Was it pilot tested? | Yes; line no. 150 – 162, details provided in ***S2 File*** |
| 18. Repeat interviews | Were repeat interviews carried out? If yes, how many? | Yes; line no. 173 - 175 |
| 19. Audio/visual recording | Did the research use audio or visual recording to collect the data? | Yes; line no. 181 - 183 |
| 20. Field notes | Were field notes made during and/or after the interview or focus group? | Yes; line no. 181 - 183 |
| 21. Duration | What was the duration of the inter views or focus group? | Included; line no. 172 - 173 and 183 |
| 22. Data saturation | Was data saturation discussed? | Yes; line no. 131 - 133 |
| 23. Transcripts returned | Were transcripts returned to participants for comment and/or correction? | No; transcripts were not returned to participants for comment or correction due to logistical constraints. However, accuracy was ensured through audio recordings, detailed field notes, and verbatim transcription |
| **Domain 3: analysis and findings** |  |  |
| Data analysis |  |  |
| 24. Number of data coders | How many data coders coded the data? | Included; line no. 201 – 209, |
| 25. Description of the coding tree | Did authors provide a description of the coding tree? | Yes; 201 - 218 |
| 26. Derivation of themes | Were themes identified in advance or derived from the data? | Included; line no. 210 - 218 |
| 27. Software | What software, if applicable, was used to manage the data? | Included; line no. 210 |
| 28. Participant checking | Did participants provide feedback on the findings? | No; participant checking was not conducted due to logistical and financial constraints that made it unfeasible to re-engage participants after data collection. However, credibility was ensured through team-based coding, use of verbatim quotations, and triangulation across data sources (AYAs, parents, teachers, food vendors, district and civil society staff) |
| Reporting |  |  |
| 29. Quotations presented | Were participant quotations presented to illustrate the themes/findings? Was each quotation identified? (e.g. participant number) | Yes; line no. 260 - 552 |
| 30. Data and findings consistent | Was there consistency between the data presented and the findings? | Yes; line no. 243 - 552 |
| 31. Clarity of major themes | Were major themes clearly presented in the findings? | Yes; fig.1 and line no. 243 – 552 |
| 32. Clarity of minor themes | Is there a description of diverse cases or discussion of minor themes? | Yes; line no. 243 - 552 |
