# Supplementary material for: Socio-ecological factors influencing dietary behaviours among adolescents and young adults in rural Eastern Uganda: A qualitative study
Source: PLoS One. 2025 Dec 2;20(12):e0337797. doi: 10.1371/journal.pone.0337797 (PMC12671741; doi:10.1371/journal.pone.0337797)
Supplement: S4 File — Empirical framework illustrating socio-ecological factors influencing dietary behaviours among AYAs in Mayuge, rural Eastern Uganda. (DOCX) [file pone.0337797.s004.docx]

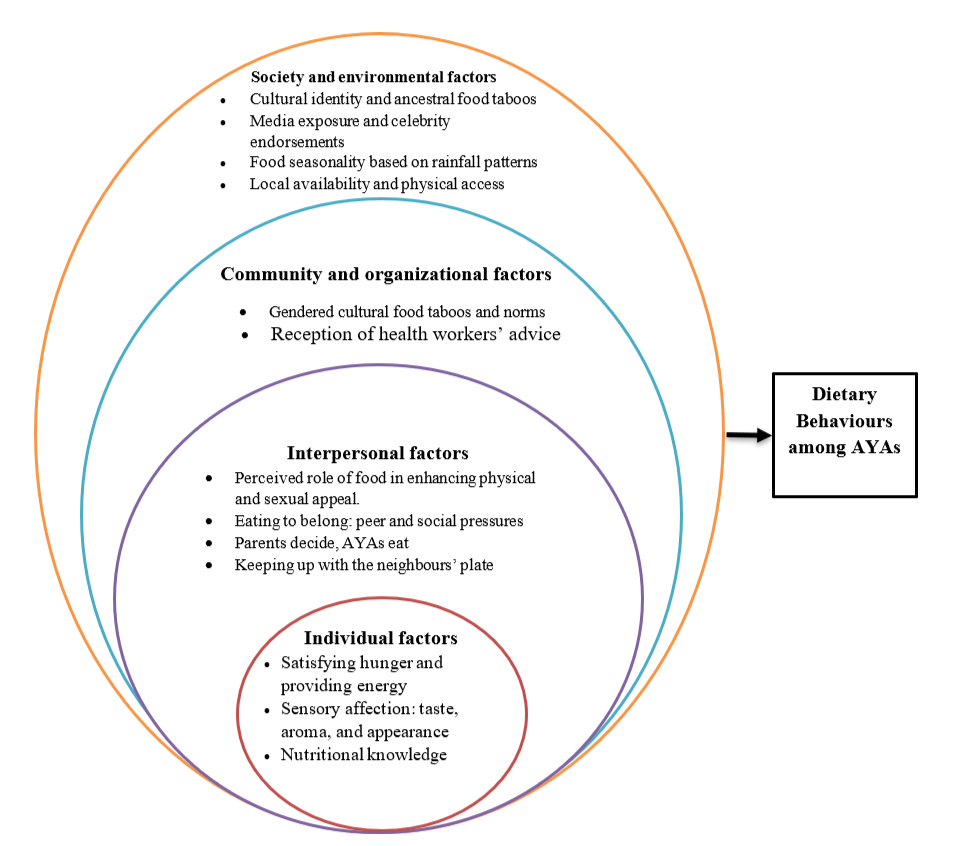


**Fig 2:** Empirical framework illustrating socio-ecological factors influencing dietary behaviours among AYAs in Mayuge, rural Eastern Uganda
