# Supplementary material for: Socio-ecological factors influencing dietary behaviours among adolescents and young adults in rural Eastern Uganda: A qualitative study
Source: PLoS One. 2025 Dec 2;20(12):e0337797. doi: 10.1371/journal.pone.0337797 (PMC12671741; doi:10.1371/journal.pone.0337797)
Supplement: S5 File — De-identified transcripts from focus group discussions and key informant interviews analyzed for this manuscript. (DOCX) [file pone.0337797.s005.docx]

**FGD_Boys_10-14yrs_In school_June_2024**

| **Participants number (R)** | **1** | **2** | **3** | **4** | **5** | **6** | **7** | **8** |
| --- | --- | --- | --- | --- | --- | --- | --- | --- |
| **Household size: Participants should have different household sizes (those from small and large household sizes should be prioritized)- insert number of people** | 08 | 05 | 12 | 06 | 14 | 13 | 05 | 07n |
| **Household heads: Participants should have different household heads (A- single parent household, B- two-parent household, or C-other guardians)** | A | A | B | B | C | A | C | C |
| **Marital status**   1. **Single never married** 2. **Married/staying with partner** 3. **Separated/divorced** 4. **Widowed** | A | A | A | A | A | A | A | A |
| **Disability (A-Yes ; B-No)** | B | B | B | B | B | B | B | B |
| **Subjective SES: A-above average ; B-Average ; C-below average** | B | B | B | B | B | B | B | B |
| **Religion: Participants should have different religions** | Participants represented different religions (catholic, protestant, born again, Muslim and others) | | | | | | | |

VENUE: At a primary school in Mayuge

Moderator (M):

**Time is 8.54am**

**Let’s begin, have you ever heard about healthy?**

All: Yes.

**M: So what does being healthy means to you?**

R1: Healthy eating meals that eating well without missing meals because there is plenty of food.

R2: I think that healthy eating means eating food that is body building and body protecting foods like bananas.

R3: Healthy eating means eating food that gives me energy and has nutrients that help in preventing the body from getting diseases.

R4: In my opinion its eating food that increases the blood supply in your body like greens.

R6: I think its eating food that’s full of nutrients that are helpful in your body.

R7: I think its eating foods which have both proteins, vitamins, iron and carbohydrates.

**M: Ok. Thank you so much.**

**We shall look into the foods ahead.**

**What factors influence your food and drink choices**

R1: I always first consider the kind of food that is tasty but not harmful. I don’t want to buy foods that will harm me like Gorillos. I ate them once but I know it’s not healthy.

R2: I chose the foods I eat because that’s what I desire to eat at that time.

R3: I chose foods based on the cravings for the foods.

R2: As for me it’s the desire to eat something. When I desire to eat some foods, I go for them as long as they are not harmful.

R6: Doctors advise that we eat food that will soften our digestive system and that’s what influences me with making food choices.

R7: For me it’s the hunger which influences me to eat what I eat. Although I reduce on chapati because they cause constipation.

R8: Usually, we eat what our parents prepare, what is available at home. Sometimes during the sweet potato harvest season, you find that we eat sweet potatoes because it’s what our mother always prepares.

**M: Ok. What motivates you to eat a healthy diet?**

R4: I get motivated to eat something due to the nice aroma.

R7: I always first consider the kind of food that is tasty and sweet but not harmful.

**M: Give us an example of that food with aroma.**

R4: For instance, you can be moving on the roadside and you smell the nice chapati aroma then you look for the money to buy.

R8: Whenever I have some money on me I get motivated to eat healthy.

R6: I am always motivated to eat a Rolex because it always have eggs in it.

R1: We have a food vendor who sells chapati at home and sometimes the aroma of the chapati really motivates me to buy from him.

**M: Any other? If none lets continue.**

**I: What challenges do you face when it comes to eating a healthy diet?**

R3: Much as mentioned the nice aroma of foods, we always get a challenge of finances to buy those healthy foods.

R2: I also fail to eat those foods due to lack of money.

R8: I like eating fish and eggs but I don’t have money to buy them.

R7: Sometimes our parents desire to give us healthy foods but fail due to lack of money.

**M: Someone mentioned greens earlier. What about greens?**

R7: The challenge with where I stay, greens grow along the road and behind latrines and people urinate on them, so I feel it’s dirty.

R8: Greens are also sold and they are very cheap but sometimes we don’t have money.

Secondly people say we should wash greens more than twice because they will lose vitamins yet they are urinated on. Greens grow behind latrines

**M: ok.**

**Thank you.**

**Let us discuss your surroundings when it comes to food.**

**What is your opinion on the dietary habits of young people in your household and community?**

R4: Young people have a habit of eating pizza, kikomando, Rolex.

**M: Which kind of pizza?**

R4: that’s a chapati mixed with eggs and vegetables then you fry and it becomes a pizza.

**M: Ok.**

R2: Most young people prefer eating deep fried foods like fried chicken, or chips.

R1: Whenever there is a function in the village, young people go for many rounds of food. They keep changing from one serving station because they don’t get satisfied.

R6; Others go for 5 rounds.

**M: Wow!**

R7: Some of them make sure they look for the cooks and ask for all the remaining food in saucepans. Sometimes they volunteer to carry food, carry saucepans, clear the plates so that they are added more food.

**M: What else do they do?**

R6: They always come back home with a chapati or fried and add on the food they are given. The problem is they don’t want to share with others. You really have to beg. They really like those fried foods.

R7: They also prefer eating roasted meat and rice. And they can dress very well even when they are going to buy food from vendors.

Most young boys want to fit with their peers that why they strive to make money so that they eat good things together.

R1: I have a brother at home who behaves different from others. He doesn’t want to eat food from vendors. He only prefers home cooked food like cabbages, greens, gnuts sauce, posho, rice. He doesn’t go well with fried foods,

**M: What about those other boys who are 10 to 14 like you?**

R1: They like eating greens but the older boys prefer deep fried foods and when they have money they don’t Miss Rolex, pizza, kikomando.

R5: The young boys who buy chapati and the like from vendors always come back home when they are not full.

**M: So what do you prefer eating yourselves?**

R8: I love eating posho because it give me energy to do my work well. I am not a matoke person.

R7: I love eating greens, posho ad beans.

R6: I like eating eggs, posho, matoke and greens.

R2: I like eating cassava and sweet potatoes because they make me full.

R4: I eat posho, eggs, beans, rice because I like the taste.

R1: I prefer eating greens that is well fried without any soup in it with posho, Yams ad sweet potatoes.

**M: What food and drinks do vendors in your community sell?**

R4: They sell katogo (cassava with beans), fried rice with beans and spaghetti.

R6: They usually sell cassava chips, matoke and sweet potatoes.

R3: They sell porridge, rice and katogo.

**M: What about those who sell to you at school?**

R3: They sell cassava chips, rice balls, katogo, and samosas.

R2: They sell cassava chips coated with curry powder. They also sell porridge at the road side.

R6: They sell fried cassava chips and boiled cassava coated with curry powder.

R7: They sell mandazi, chapati, and kikomando

**M: Ok.**

**So my next question.**

**I: What food and drinks do you typically buy from food vendors?**

R6: I usually buy cassava and katogo from them.

R1: My mum usually buys for me fried rice, katogo and rice balls from vendors.

R8: When I go to the food vendors in the trading center, I buy soya porridge mostly.

R5: I buy sweet potatoes and milk from vendors.

R7: I don’t have choices, I just buy whatever I come across.

**M: Ok.**

R4: I usually buy yoghurt, sugarcane and katogo.

R3: I like buying pancakes and katogo.

**M: Do the vendors bring katogo at school?**

R1: They do bring.

**M: Ok.**

**What factors influence you to purchase food and drinks sold by food vendors?**

R1: I purchase them in order to get energy in my body.

R3: at times Iam very hungry because I leave home without any breakfast, that’s why I buy from vendors.

R: Iam influenced whenever I have some money in my pockets. I don’t settle whenever I have money in my pockets.

R5: Sometimes the type of food we are given at home influences us to eat.

**M: What are your opinions, as well as those of young people in your household or community, about food and drinks sold by food vendors?**

R3: My opinion is that some of these vendors who are still young are making money to go back to school because they are school drop outs.

**M: Ok.**

**What do the others say?**

R8: I don’t really trust the food that is sold by food vendors although their main goal is to sell and get profits.

R2: Those vendors can even make you eat funny things. For instance those selling chicken and roasted eat. We are not sure whether it’s really chicken or genuine meat. They may be selling dog meat and birds instead of chicken. Sometimes we think they are roasting or frying those big wild birds.

R1: There is a vendor who sells fried chicken near the petrol station ad I use to buy from him. But I eventually stopped because he only sells chicken necks making me wonder why other chicken parts were missing.

**M Did you ask him?**

R1: He can’t tell us.

R In case you have an enemy, he can even poison you through those vendors because some of them are strangers.

M: Ok.

**Now I will ask you about nutrition related health programs**

**Have you ever received any nutrition or food related assistance in school or community?**

R2: No.

**What about in the community?**

R1: One time I found some people in the market educating about the dangers of eating snacks like Gorillos. They told us that Gorillos is used as rat poison In Tanzania and also used for lighting the stove. Yet we are busy eating Gorillos in Uganda here. They told us that if we eat it, we can get complications in our tummies.

**M: Ok. Which company were they coming from?**

R1: I’ve forgotten.

R5: Sometimes we meet people o market days educating that it’s not good to take a soda before eating anything because sodas washes away the food you ate. They also said Gorillos is harmful to our bodies.

R6: One time a man was talking about **harambee curry powder** and royco chicken still in the market. He started by pouring water in the curry powders and we saw rice husks popping out in the water. So he said that this powders are made out of rice husks which are colored ad not good for our health.

R5: There is another one who came with **Royco chicken** and poured water in the contents, then we saw some small metallic pieces there.

**M: Did you also see the metals.**

R7: We saw them at the bottom of the container.

R1: That very ma also taught us about pads that girls use especially the brands of Always and Princess. He said that **Princess pads** coati a chemical that looks like cotton yet it’s not cotton. He then poured water on that pad and covered it then we saw maggots.

**M: Are they the same people who were teaching about the Harambe curry powder?**

R1: Yes the same people. They also said that pregnant who should not eat Royco mchuzi mix because it contains dust and small stones which can affect the baby.

R7: They also told us that we should desist from taking tea with Ruwenzori tea leaves because it has a chemicals which causes miscarriage.

R8: I also met them teaching that we should be careful because some of the spaghetti, eggs that we eat and rice is full of chemicals. They said there is rice from china that’s made of plastic and that we should be very careful.

**M: Ok. Let’s continue.**

**How have these programs influenced your dietary or lifestyle behaviors?**

R1: I stopped eating Gorillos from that day.

R4: I stopped using harambe at home though I eat from school when I buy cassava coated with harambe curry powder.

**M: O**K.

**What kind of programs OR Interventions would help young people like you to have a healthier diet and lifestyle?**

R4: We need a health nutrition sensitization program

M: Ok.

**M: What is the Scope of activities?**

R2: By organizing nutrition counseling sessions.

**M: Ok.**

**Who are the Target group?**

Adolescents our age groups in and out of school.

**M: How should it be done?**

At schools through workshop, community outreaches and at the workplaces of the out of school.

**M: Let’s look at the potential challenges.**

R5: Some youth’s dont want to attend health programs

**M: What ways can they be addressed?**

R7: Educate them with demonstration materials of health issues arising from poor nutrition habits.

Demonstration from educative videos. Charts, IEC materials

| **Program 1 title** | **Scope of activity** | **Target group** | **Delivery mechanism** | **Potential challenges** | **Ways to address challenges** |
| --- | --- | --- | --- | --- | --- |
| We need a health nutrition sensitization program. | Organize nutrition counseling sessions | Adolescents our age groups.  In and out of school | At schools through workshop.  Community outreaches at the workplaces of the out of school. | Some youths don’t want to attend health programs | Educate them with demonstration materials of health issues arising from poor nutrition habits.  Demonstration from educative videos. Charts, IEC materials. |

**I: Is there any other intervention you are thinking of?**

Thank you for participating.

**FGD_BOYS_15-19 YRS_IN SCHOOL_June_2024**

| **Participants number** | **1** | **2** | **3** | **4** | **5** | **6** | **7** | **8** |
| --- | --- | --- | --- | --- | --- | --- | --- | --- |
| **Household size: Participants should have different household sizes (those from small and large household sizes should be prioritized)- insert number of people** | 04 | 03 | 10 | 04 | 06 | 02 | 11 | 06 |
| **Household heads: Participants should have different household heads (A- single parent household, B- two-parent household, or C-other guardians)** | B | A | C | B | C | A | B | B |
| **Marital status**   1. **Single never married** 2. **Married/staying with partner** 3. **Separated/divorced** 4. **Widowed** | A | A | A | A | A | A | A | A |
| **Disability (A-Yes ; B-No)** | B | B | B | B | B | B | B | B |
| **Subjective SES: A-above average ; B-Average ; C-below average** | B | B | B | B | B | B | B | B |
| **Religion: Participants should have different religions** | Participants represented different religions (catholic, protestant, born again, Muslim and others) | | | | | | | |

**Venue where the interview was conducted:** At a Primary School in Mayuge

**Moderator(M)**

**Transcript**

**Thank you so much now if I may ask, what do the youths here in this village enjoy most?**

R6: Youths in this community mostly enjoy football, music and entertainment. In addition to that they like working together and they engage in Saccos also.

R8: I just want to put something right like he said they enjoy football but generally they enjoy all the sports not only football.

**M: So now if I may ask; I want to understand, what does a youth from this village regard as being healthy like I want to know what being healthy means to you or to another adolescent here in this village?**

R7: What they regard as being healthy is they have to be having the following things firstly they have to have their own peace like when they are not suffering and being treated badly that can make them healthy because they be not overthinking you know when you have a lot of thoughts there is a way it can reduce on your life expectancy. In addition to that, a healthy youth from **this village** has to eat all the meals of the day, they have to maintain a balanced diet then also to add on a youth from **this village** in our age group has to be bathing and cleaning their clothes even where they sleep so that is the healthy life in my understanding.

**M: So they have to have their own peace, having a balanced diet and all the food… I wonder what a balanced diet is!**

R7: All food values.

**M: Okay we shall reach that part and you tell me something about that because I have come to learn from you… so who wants to add on that?**

R5: A youth from **this village** if they have to enjoy life very well they have to have a job because it is that job that can enable him to get money to meet their needs at the moment so it is helpful for the healthy being of the youth here in **this village** then the rest my brother over there has said it all.

R1: I want to add that we the youths we need to have jobs because there is a way that job can keep you busy when you are working all the time so it is needed for it keeps you busy.

R4: They have to be doing some physical activities like football, running all that to keep their bodies physically fit.

**M: Now you have told me about the healthy being of a youth you have said they have to be working, they have to be doing some physical exercises, they have to be bathing and then they have to be maintaining a balanced diet then also peace… so when you talk about being healthy, what comes to you mind when you hear the word healthy?**

R5: Being healthy is a lot and it includes many things one; you have to be cleaning your body like you have to brush your teeth regularly or daily then you have to bathe and clean your whole body. Then you have to clean your private parts because they can reach a time and they start smelling so for me that is how I understand a healthy person should be.

**M: Thank you R5…**

R8: It is like the other first things that a person needs to have their own peace then also be engaged in games and sports and when they have a job where they can get money and also to eat very well.

R4: In addition to that they have to have access to medical care so that they can check on him and his body status so that also helps a bit.

**M: So you think a healthy person has to have access to medical care… R6 had something to add…**

R6: To add on what my fellows have said for a healthy person what comes to my thought is that the person’s body has to be free from sicknesses and even the food they eat has to be good for their body.

**M: We are going to talk more on the good food, how is that supposed to be?**

R1: When we talk about food there is me eating a balanced diet like you can eat some Matooke, some posho… but how are you eating and at what time there is eating in the morning and then your next meal is in the evening but that is dangerous to the body and there is no way you can be healthy because you can easily get ulcers.

R7: The other thing I understand, to add on that a youth from **this village** or even in the places around in Uganda… all youths have to be careful with their sexual behaviors they can either abstain or consistently use condoms so that can help them be healthy and to add on that like if they wake up in the morning they have to wash their face because when you do that it helps for the eyes not to get sick and when you wash the eyes of course you have to wash the whole face so in turn you be feeling very well and very healthy.

**M: So what has helped you to know and understand that it is those things that help to live a healthy life… what has shaped your understanding of being healthy?**

R1: What has helped us to know that is the fact that we come to school and study so they teach us that as a person you have to have good personal character when you are eating and cleaning yourself in order to keep your body healthy.

R3: What has helped to shape my understanding is that… I am going to add on what my friend said that for a healthy person it depends on their body is, seeking medical care with nothing disturbing their body and also getting a balanced diet… eating that good food, fruits generally things that can put strength back in the body so that they can live.

**M: Now for you what has helped to shape your mind that it is those fruits that help in staying healthy?**

R3: There are some things that contain food values like proteins, carbohydrates which help in body building.

**M: Still that brings me back to ask how did you get to know that those are good for body building?**

R3: They taught us like that.

R5: To add on that for me I can go and consult from medical doctors and when I reach there they tell me that you have to be like this and this in order to be healthy or I can attend health seminars that take place in our communities in order to know that I must be healthy. Then another thing some people die on the villages so for example someone may die just because of dehydration so I’m in the position to know that if someone dies I can know that a person must be healthy by doing this and this.

R7: How I know about healthy life is I often watch health programs on TV and on radio stations when they are talking about being healthy so a person like me who hears that and learns I can know how I can best keep my life and how my grandfathers lived and how best we can keep the generation up to the next generation so hear that on the health programs and the teacher we have here they have done a great job to teach us about life they tell us to brush our teeth in the morning so generally they tell us how to keep ourselves clean.

**M: R5 has talked about what he has called community health seminars, do those usually happen?**

R7: They happen and the chairman holds meetings and he brings VHTs and they come to teach how a person can be healthy one; a person has to be having a toilet at home and that can help to prevent diseases then the other thing you have to be bathing as to also prevent diseases and also to promote general sanitation at home so the chairman and his team they have really shaped our minds so that is that.

**M: Now I want to know more about the dietary habits, what does a healthy diet mean to you?**

R1: That has a way it enlightens me that good feeding is not that I have to eat posho for example all the time but instead I have to change a bit like I can eat some posho then eat some potatoes, some greens so I keep on changing.

R4: To me healthy diet I understand it in a way that you eat all foods containing all food values like proteins and others.

**M: What are the others?**

R4: Carbohydrates, Vitamins, lipids, fats & oils so all those fall in that same category.

**M: Anyone with a different understanding about the healthy diet!**

R8: To add on that I think it is having meals on time like how you can be that in the morning I will take some tea before coming to school then breakfast at around 10am then at 1pm or 2pm I be having lunch so then the day goes like that minus these things of I come from home without eating and then up to the time I go back then I eat…

**M: You have told me about the healthy diet what I have understood is that you have to be eating food that has all food values and then also eating in time and then you have also told me that also when the cleanliness is there…**

R: The other thing is that you don’t have to over eat or under eat so you have to eat just what is sufficient for your body.

**M: So I would like to know what factors influence your food and drink choices?**

R5: You have asked the factors that influence food and drink choices and one is poverty because you might want to eat something but when you don’t have money to get it for yourself. Then diseases or sickness like you can want to take some sugar but when you are sick of diabetes so that cannot allow you to eat then we also have what they call cultural factors for example you might reach there and they say for us in our clan we don’t eat this and this for example like at ours they don’t allow us to eat meat from a cow that has stains on its body so there now you ask yourself that how will you get the proteins, the fats and the lipids without eating meat yet the meat is the source of fats so that is my submission.

R7: Me I think it is the geographical location like in town for them they eat those… geographical location like in villages they eat bananas because of their location.

**M: So you mean in town they don’t eat bananas!**

R: They are limited.

R5: Let me add something on his point; now for example People in Mbarara have bananas because they grow a lot of them. In Busoga, it’s mostly sweet potatoes; it depends on where you come from, and this side in Busoga there are sweet potatoes therefore those people eat Matooke because of their geographical location.

R1: What I want to add on that is that the most thing that influences the choices is the money in the pocket let’s say you are there in the city and a banana is at 500/= but when you have only 200/= so you be limited there.

R7: The other thing that influences, is the language now like for the Banyole me I am one of them both my parents are so now there in Budumba is where we are from and for us we mostly eat cassava flour. As a Musoga, I come from a clan that prohibits eating certain foods, especially those considered to be totems. We also eat a lot of sweet potatoes; it's part of our culture.

R6: To add on what my colleagues have said there is what they call long distance. Even when you have money, it’s hard to get juice if the shop is too far from where you are

**M: What motivates you to eat a healthy diet… because you have told me that a healthy diet includes eating in time, eating food that contains all the food values so like wat motivates you to eat a healthy diet?**

R1: What motivates us is wanting to fulfil all that the doctors tell us to do that when you eat this and this you get the necessary nutrients in your body then others eat just because they have taken long without eating that particular kind of thing so they eat because they have wanted it and they have the money to buy it then another can be there when the motivation comes from the rest of the people they are with because they are also eating so that they can fit in the group.

R3: The other thing which can help us is that we want to prevent the lameness of our body if you don’t eat some food values… that can also help us in our health.

**M: How?**

R3: When we eat good food, it helps us to avoid certain things like sicknesses like kwashiorkor, marasmus and all those others even polio… generally like that. But also, I love eating the kind of food I see in movies; it always looks so delicious.

R7: Firstly, what motivates us are the friends like I can be coming to your place and then I find when you have some fruits yet for me I don’t the possibility to get it so I can come to your place with my friend and we eat some things together so when I come and find that there is a change in my body I can also get motivated so that I can also get a balanced diet in my daily living.

R8: What motivates me is that… now where I have studied from, the teacher at school taught us that we have to eat a balanced diet so that we are healthy, so for me, I always try to eat a balanced diet. Then the other thing is that I can look at the neighbors and then desire food they have eaten, then we also go back home and decide to eat what they have eaten.

R3: The other motivation is that the food gives us strength in our bodies so there are some energy giving foods like I can be working in a factory when they give me heavy work but if I eat like posho and beans I can get energy very well without any disease disturbing me and when I am not feeling anything.

R5: What now that I ride a boda-boda, I select the food that gives us strength and energy in our bodies. For me, when I eat like posho and beans, I can get energy to dig very well.

R6: To add on that, what they have said… what motivates us to eat a balanced diet is the income you might be there when your earning per month is like 50,000/= that can lead you to go and pick at least 10,000/= and then go and buy some meat then you eat then also get some 20,000/= and buy some passion fruits ad nice drinks and bring for the family to drink but when you are not working even though you are attracted but when you are not working and not earning anything you cannot do anything.

**M: I would like to know, what are some of the challenges that we face when it comes to eating a healthy diet?**

R6: It is expensive of the items so you can reach a place where they sell meat and a Kg is 20,000/= but when you have 10,000/= so you cannot buy the thing you want to eat.

R5: To add on that is the poverty so when you don’t have any money you cannot go and buy a Kg of meat so that is the challenge and then the sickness of someone this can also be a challenge for example they can be sick of diabetes and they don’t eat sweet things so that can also be a big challenge that hinders those people from eating a balanced diet.

R1: What hinders in most cases is the large number of people at home like you can be 10 people but then you would like to have some meat but then they think of how they are going to serve it and they find it won’t be enough so that becomes a challenge…

**M: It is like as though you are telling me that healthy diet is all about eating meat… please share with me other challenges that are hindering you to eat a healthy diet just like you explained it earlier.**

R7: The other challenges are… you could be wanting to eat some greens and by the time you go to find it in the restaurant, you have it and it tastes so good for you but when you find the place where they got it from it is a surprise for you to find that the place is near the toilet so I can never eat greens again and I also go ahead and tell other fellows of mine that they get the greens from near the toilet so don’t eat so it becomes a challenge.

R3: Food taboos… those things of saying that this clan or group of people are not supposed to eat this and this are not good and they can hinder us to have a lot of things for our bodies…

**M: Now for you what clan do you belong to?**

R3: The Ngabi clan…

**M: So you don’t eat it!**

R3: Yes of course I don’t eat it…

R: It is what he has just said about the food taboos like how this one told us that in their clan they don’t eat meat from a cow with stains on the body so it hinders them to eat certain things.

**M: What happens when you eat meat from that cow?**

R: Like you know for these food taboos when you eat that there is something that happens on to your body so now like for us you can get skin rushes when you eat it.

R: Another thing is… like how this one said that they grow cassava only now that becomes a challenge because they cannot get access to rice because they don’t grow it…

R2: There are also some discriminations at home like because your father bore you from another mother and then he has another wife at home so when he brings something for you they don’t give you.

**M: Explain to me so that I get it clearly…**

R2: Like when they bring something to eat they be like this child is for my co-wife so I will not give him so they only give to their biological children and for you they don’t give you at all.

R: Let me also add on just one more… for example it depends on the location like you might be coming from town when you have to buy a mango, avocado is for buying and every other thing you just have to buy so you will not access some of the things but then for me who is in the village I can just go there in the garden and get some maize and eat and also get a mango minus anyone telling me anything so the location can also be a challenge.

**M: We are going to discuss your surrounding when it comes to food, so what is your opinion on the dietary habits of young people in your homes and community?**

R1: Most times at home we can have posho then for me I don’t get satisfied so if I go to the trading center there if I have some 1,000/= I top up with like some rice, spaghetti and stuff like that.

**M: So what about a person who has no money what do they do?**

R1: That one with no money if they be in the village then they can go and look for some fruits and eat that.

R5: For me I think the peer groups for example I might be walking with this friend and for them they eat a whole Kg of rice so now as for me to fit in the peer group I end up also eating a whole Kg of rice yet you have not even intended to do so.

R: For me in my thinking I would advise us the youths to put teachers’ instructions and teachings into good use in order to eat good food that fit for our bodies.

R: In addition to what they have talked about there is being there and for example I can be addicted to using drugs and after I have used my drugs there when I reach at home I find food that is just there like that and it is very small because they say that after using those drugs there is a way they increase your appetite to eat a lot so in the end you finish when the food you have eaten is not enough for you then you decide to go elsewhere to add yourself some food so you can add on some chapatti, rice that is if you have some money and then if you don’t have money then you end up eating sugarcanes and then some jack fruit and to worst point it can cause you to steal.

R5: Let me add on this, for me in my thinking I think that the employment opportunities for example there is a place there where they collect sand from most youths go there and they get jobs to work on sand and when it reaches at night they eat chapatti, mandaazi so therefore I think when you have a job there is a way it brings about someone eating even more than they have to.

**M: I would like to know here in this village, what foods and drinks do vendors in your community sell?**

R1: For us here the things that they sell most some of them are posho, sweet potatoes and cassava then for the drinks mostly it is soda reason being that they have a mentality that when you take some soda then you be spending your money very well.

R5: For the drinks just for me I live down there but there are very many drunkards so they sell alcohol more as a drink but for the food just like my brother has said there is a posho, potatoes, jack fruit like you know all the eats that usually be in the village are there…

**M: Even the youths buy the alcohol!**

R5: Yes, they do buy it to pass time.

**M: What about here at school in this environment, what foods and drinks are mostly sold here?**

R7: From here up to the center there I know they sell some chicken so when you go there you find it there, fish, Irish potatoes so even here at our school in case the doctors need to keep some balanced diet they do have them.

R8: For us at school they do have cassava more because it is cheap even if I come with some 300/= I can buy and eat… that fried cassava.

R1: Here at school what we mostly buy are the chapattis and yellow bananas…

**M: I was going to ask that which foods do you mostly buy from these food vendors?**

R1: Chapatti and yellow bananas because they are the commonest things here they are the ones they usually bring whenever we go and buy they are the ones available and still very less expensive…

R: A yellow banana is 100/= so you cannot go and buy bread of 2000/=.

**M: What other foods do they sell apart from those that you have mentioned?**

R1: They often sometimes sell pancakes, samosas…

R7: To add on that they sell water melons and avocadoes then also paw-paws then also the pineapples are also sold.

**M: What factors influence you to buy those foods that you buy from food vendors like R1 has said they buy mostly chapatti and yellow bananas because they are the cheapest meaning that money is a factor therein…?**

R5: I think let me give this… health factors for example you can go to the hospital and then the doctors tell you that if you want to live start eating some fruits like mangoes, yellow bananas and stop eating rice and things like meat to be able to keep your life very well so that is why I have told you that the health factors like when you are following the medical rules and regulations.

R3: The other thing that influences us is the fact of taking long to eat something so you can be there and then you are like you have taken long without taking some soda so the moment you get some money then you go to the shop and buy it if it is food you can buy rice and eat some…

**M: Which food do you take long to eat and you are like the moment I get money like this now like here as when we finish I am going to give everyone some compensation so what food have you taken long without eating…?**

R: Not to tell you lies I will buy rice because for us we eat it on special days at home to be sincere.

R: Even me that is what I’d do.

R: For me Irish potatoes…

**M: So now, what do you think about the foods or fruits sold here at school and in the communities?**

R1: We like those fruits because even the teachers tell us to eat them a lot so when they also teach us here and we learn we also go and inform or educate our grannies at home and other friends of ours who dropped out of school.

R5: Me also I think those fruits are also good because they help us to be healthy and being there when we have energy because they have the vitamins they are broken down during sterilization to release energy so then when you do eat the fruits you be having strength and then you are able to go and work.

R7: Me I see that they are sweet and because of that it makes me to get attached to that very thing but if I taste it and it is sour then there I will start avoiding it because now the youths in our generation we do like fruits because they are good.

R6: What attracts us to those fruits is that they are easy to get even if you have 100/= you can get and then you can be there when at your home there is a mango tree when it bares mangoes you go and eat or even you can also grow your own yellow bananas and when they are ready you harvest and then you eat.

R8: For me what attracts me to eat the fruits it is because they can quench my hunger I can be there when I am hungry and when I eat them I get satisfied.

R3: The other thing is that most times we usually watch programs on TV when they are teaching about the benefits of eating these things and the doctor can be telling you that if you have fats in your body you can take some lemon it can help you or it can even help if you have a constipation you can eat some bananas then you be okay.

**M: I want to ask about nutrition programs in this community where we started from you told me there in your community there are some times where the VHTs come and talk about nutrition so those are the programs I am talking about so you tell if you get them sometimes here at school do you get them or not?**

R6: That program often happens here at school and the teachers are the ones who do that they call for an assembly then they counsel us then give us guidance then they tell us about those fruits and then how we can keep our bodies healthy.

**M: How often does this happen at school here?**

R6: There are no specific days but there is a time that comes… I think they discuss in a meeting then they be like let us talk to these children about nutrition and other things.

R: For me when I had just joined S.1 there came some doctors who wanted blood from us then after they go the blood they told us ways we can maintain the nutrition but their aim was to get blood.

**M: We are looking at nutrition or food related assistance in school and you old me that here in school you have ever been taught about nutrition then the other one told me that doctors who wanted blood came here and after they got the blood they talked to you still about nutrition… is there any other nutrition program here at school?**

R1: There are some doctors who usually come here in the community… they often come and tell us about nutrition and what we need to eat to stay healthy.

R8: There are some doctors who still come to us there those VHTs and the chairman they come sensitizing about health and nutrition.

R7: There are some doctors from certain health centers like FUKANG who pass through with a drive with speakers calling for people to go and get blood check-ups and then they tell them how to keep heathy and to have enough blood in the body meaning it was also nutrition related…

**M: Now these programs about nutrition that you have talked about, how have they influenced your dietary habits?**

R6: How they have influenced is that after they finish telling people other people take it and then put what they have been told to work and after they start following those steps…

R5: When those doctors came they told us that for you to be healthy it doesn’t need you to eat a lot of food and stuff like that so it helps me as a person to know that I should not over eat because good nutrition doesn’t come so because of over eating.

R7: In addition to that, the doctors told us here in this community that it is not only the balanced diet that will keep you healthy but you have to be eating well prepared food let us say if the cook has cooked for us posho or even at home if it is left uncovered and flies come and fall on it then there it is contaminated so you will get sick so they told us that if it is being healthy then the balanced diet has to be there and even what we eat has to be clean. Then also to keep proper sanitation cutting the hair, cutting the finger nails, brushing of teeth then there we shall keep healthy and life will move on smoothly.

R: In addition to that still for example those doctors they always come and encourage the family members especially the elders that for a person to be healthy it doesn’t only mean that they have good nutrition but also having a toilet at home is necessary but still even if you don’t keep sanitation you will still get sick.

R: To add on that still those same people come to this place door to door encouraging people doesn’t mean that you have to be having a toilet and proper disposal of rubbish only but you have to sweep the compound and have a small jerry can behind the toilet after easing yourself you can wash your hands so that you keep proper sanitation and hygiene.

**M: Now the other question is what kinds of programs would help young people like you to have a healthier diet or lifestyle?**

R6: The first program would be the physical program like taking part in the physical activities I think can help us as youths so that our bodies are healthy then the other thing is for us to keep ourselves clean so that we can prevent diseases and also to eat food that has food values to keep healthy.

R8: The guidance and counselling programs at school they help me because I get a lot from them how to keep myself and also how to eat well.

**M: Guidance and counselling on what…?**

R: They tell us about many things for example our lifestyle and how we are supposed to eat and many others.

R1: I am still on that health program and this includes many things like physical activities because there is no way you are going to be healthy when you are no doing physical exercise it is like eating a lot of food when there is nothing you are going to do and you are going to just sit so those health programs help us the youths because for us youths we have to go and do other things.

R: Me I think that the nutrition program will do because all of us know that nutrition combines all that does with the food values like vitamins, carbohydrates and all the rest so for me I see that the program of growing vegetables and fruits is a helpful one to maintain our body nutrition.

R4: I think that farming can be a good program that can lead us to eat good food that if they are growing crops on infertile soil it can lead to poor yields so the farming program is the one that can help us.

**M: We have seen programs these are the ones I have written namely; physical exercise, sanitation, nutrition counselling and also to grow fruits and vegetables so I want to look at these tell about this program of growing fruits and vegetables which people should it target then we look at how it can be done and the challenges that can be involved and how we can solve the problem in implementing this program…**

R6: You have said the people it should target I think it should target the youths between the age of 15-30 because it can help them if they are in the garden growing those crops those other vegetables need a lot of time so it is going to keep them there when they are busy then after they will get money out of that which can help him do other things and they will be able to serve the community and the vegetables the community needs.

**M: How can program be delivered?**

R1: Why I think it should be delivered it is because I see almost the whole country is supported by agriculture because almost all the eats are just grown and there is no way you can get what you want to eat without agriculture especially here in the village for the people in urban centers they have no where they can do it from but for us here we have to… but the reason some people do not grow crops is that they don’t have where they can grow them from sometimes they can want but then they don’t have where to grow crops from and someone can even have the space but when they have no seeds.

**M: So how can we solve the problem of lack of space for growing crops?**

R: If you have no space then team up with certain people and after you are in that group it is not possible that all of you can lack space but when you make a groups this one can have seeds or the other one can have money to buy seeds and then you are able to grow crops.

R4: In addition to that, we can use what they call sack gardening; for example, you can go and buy tomatoes and you get something like a tin then you get soil and put in that tin then after you put your crop in that even though you are in a small area you can still carryout farming of that nature.

R7: I want to add that to carry out growing of vegetables and fruits we have to make a youth program and we team up so that we can get our own garden and if we are in a group it gives us he strength to work together as a group so that we grow our crops in plenty and then also have plenty of food through the youth program.

**M: Okay then let us go to the nutrition counselling and guidance… which people should this program target?**

R1: It has to target people like the old people from 60 years to maybe 80 years then also the youths… a youth of 20-40 years doesn’t have to eat things to make them fat and over grow because they still have the strength needed to work so they have to keep their bodies physically fit in order to work for their future then also the babies from 1-5 years they have to be given food with enough food values that are needed by their body…

**M: So how should we do the mechanism of delivery after we see the mechanism we shall go to the challenges?**

R: But for me my thought is different from my brother over here… because me I have my grandmother who is 85years old but he can tell you that my grandson please give me some mango and I also eat some because I also want to live and even if you are there and you refuse to eat then there you will die because you will get sick therefore all of us we need those vegetables so I think it should be open so all of us we need.

**M: So let us go to the mechanism?**

R6: I think there should be some organizations that should help these groups that have come to form Saccos and they want to come up with growing of vegetables so they should help in seeds, medicine because there is expensive medicine and you can drive to go to Iganga town and still find when it is not there but remember you have vegetables so if these organizations come and help then we can achieve what we want.

**M: What challenges do you think can affect the nutrition counseling program?**

R7: There is this saying that when I eat and finish it is good and when I eat and keep it is also good so both of those are good so now how it will be done we beg those above us because for us we can want it to happen but then we don’t have the capability because there is money needed there…

R: The government should support these people with things like seeds as they form Saccos and then after tell them what to do but then if there is nothing they are doing for them then there is no way it is going to survive.

**M: How do you want this program’s scope of activity to be like?**

R6: For me I think it should be from the youths between the age of 18-45 years because these youths have time and then they also have strength and they be working for their future but now these old people for them they should leave them aside because an old person just wants to sit there and rest because for them they are already tired.

R1: It should at least combine like three communities because it is not proper for a person to come all the way from that side to come this side… even when you tell a person from town coming this side they really see that you are making life hard for them and it is good if these things of growing crops they be available in the rainy season because when they choose to do in the dry season there will be need for watering and using of machines something which is very expensive.

R5: For me I would say that those things should be done in our families because it is not easy to manage something that involves very many people but if it is for the family the Dad can always say every this and this day we have to be able to go to the garden and grow some crops in the morning so I suggest that it should be done within our families.

R7: Since this is about health and nutrition I don’t think one family or three communities can decide for the whole country so what I am suggesting is that it should be for the whole country…

R1: I beg to correct on that when I said it should be at least three communities I meant it could be for the whole country but in groups of those three communities because still here there is a saying that togetherness is one so when you are many you can easily find a solution for something.

**M: I think we have reached the end of our discussion.**

**FGD_BOYS_20-24 YRS_OUT OF SCHOOL_June_2024**

| **Participants number** | **1** | **2** | **3** | **4** | **5** | **6** | **7** | **8** |
| --- | --- | --- | --- | --- | --- | --- | --- | --- |
| **Highest education level (A- Primary; B-Secondary; C - Tertiary)** | B | A | A | A | A | A | A | A |
| **Household size: Participants should have different household sizes (those from small and large household sizes should be prioritized)- insert number of people** | 2 | 2 | 4 | 5 | 14 | 5 | 6 | 4 |
| **Marital status**   1. **Single never married** 2. **Married/staying with partner** 3. **Separated/divorced** 4. **Widowed** | A | A | A | A | A | A | A | A |
| **Disability (A-Yes ; B-No)** | B | B | B | B | B | B | B | A |
| **Subjective SES: A-above average ; B-Average ; C-below average** | A | A | A | A | A | A | A | A |
| **Religion: Participants should have different religions (A-Catholic; B-Anglican; C-Born Again; D-Pentecostal; F-)** | Participants represented different religions (catholic, protestant, born again, Muslim and others) | | | | | | | |

**Transcript**

**M: Thank you for this opportunity given to me to speak to you. The first question is, what do the youths in this community enjoy most now for me I will give you an example me I enjoy football most and you also tell me what they enjoy most?**

R1: What they enjoy most is getting involved in work because 24/7 we are doing work.

R2: The youths enjoy being there when they are working.

R3: They involve in work most times.

R4: I think it is also the same to have some work to do so that by the end of the day you have some money for yourself.

**M: What about games and sports?**

R4: Yes, also that now for me I was going at this time by only that my auntie called me and told me to attend to this then after I go.

R5: What we like most here is the thing of hustling to live and have a lifestyle.

R6: Games and sports like football.

R7: Work and playing football then jogging around so that we have physically fit bodies and doing some exercises.

R8: Doing some work so that I get money to help here and there…

**M: When I talk about being healthy, what comes to your mind when I talk about being healthy?**

R5: I think it is preventing diseases.

R1: First of I think your behavior now let us say if you are so into women it is hard for someone to come and say that you are healthy then also being shabby all the time that also they cannot call you healthy.

R7: A healthy person is a person when they just look at them then they just say they are healthy like when they are walking down the road they are not kicking tins and stuff you know when kick tins and stuff they can think you are mad sometimes you move while talking to yourself then they can conclude that you are not healthy and you are mad.

R3: A healthy person has to be sharp in mind and do sensible stuff different from an unhealthy person.

**M: What does a healthy person do and what does an unhealthy person do?**

R5: They get up early in the morning and do some jogging and then in the evening they go and play some football so that alone if they see you they say that you are healthy because they have seen you jogging and doing some exercises.

R2: A healthy person does health check-ups more often to know their status.

R4: A healthy person goes to work because a sick person doesn’t work because they are always sick but a healthy person goes and looks for work and they work.

R7: A healthy person is that one even when some work comes up with in the community they call then upon and they come and then they do the work.

**M: What has helped you understand what a healthy person is or looks like?**

R4: For me I wake up and I go to work in the morning so that doesn’t make me to get sick…

R: A healthy person you can actually see it for yourself now you see for a sick person it is now cold but you can find them under the sun but then for you, you can feel at peace everywhere.

R1: When we go to school sometimes you get to know a lot of these for me the first time I got to know about this was at school and that is where I got to know about a healthy person. Then also another place is the hospitals there they also tell you about a healthy person then that is how I got to know.

R7: I can know even when I am in the community because you can call a person for work and they tell you they are not feeling well when they are not able to go and work and you can see because previously they have been affected by red eyes and it has been the case here.

R3: An unhealthy person you can literally see from their lifestyle they are not always not happy and they don’t enjoy life.

**M: I would like to know about the dietary habits of the young people so what is healthy eating to you, how do you understand it?**

R7: When you are eating greens, cabbage and all sorts of that kind…

R6: Eating some cassava, sweet potatoes…

R5: Eating carrots, lemon and then other fruits.

R: Now for a healthy diet you can eat some eggs for body building and then you can eat some fruits and also some rice because right now if someone eats some rice they feel they have eaten very well.

R4: I see others say that healthy eating is eating some meat, Matooke, rice and they forget that when you also eat greens it is also being healthy it is not only meat.

R7: I support what he has said, it is greens that helps in body building because all the time those cows are injected but you can eat beans you see sometimes we go and visit prisoners and then you find when they are eating posho and beans but when they are well body built but there has never been meat for them to eat.

R5: For me I think that when they tell us to have a healthy diet they don’t mean to eat meat but then they mean eat some greens and stuff like that.

R7: You cannot get sick and they put you on soda instead they give you water and then they also recommend passion fruit juice.

R1: For me I think healthy eating is taking things like Bushera there is that Bushera that has nutrients that are good for your body.

**M: What influences the food choices for the young people in this community?**

R7: A person can be there when their body demands to eat something you can feel like eating something and there are times you don’t feel like eating something and also sometimes the environment where you are or working in at that time you can be working under sunshine then you feel like you want to drink some cold water.

R4: I think it is the appetite but it depends on what you want to eat and there is also the yearning to eat that very thing.

R7: There is also when someone can be there and they see what to eat from others like what others eat is what they eat then also there is also someone who can buy and they don’t give their friends some and then the other one also says let me buy and eat the thing that the other on has refused to give me some.

R6: For us boys, when we eat posho and beans, we get the energy for do heavy work like making bricks or loading sugar canes. It is about having energy to keep working to make money.

R1: There are some youths amongst us who use drugs and so you might find that you go and use drugs and they increase your appetite to eat what you can find.

**M: What motivates you as a youth to eat healthily?**

R8: When you get the posho and you want to eat it…

R1: I just eat what is there… mostly cassava and dry tea [black tea]. I don’t know much about which foods are healthy.

R2: Your capability also motivates you to eat healthily.

R7: Like how he has said that your capability is the drive so you cannot go and get meat if you don’t have money or even chicken those are the people you see when they don’t have but then they are all over where they don’t belong and we have more of those people they be there when they are bragging but when they don’t have even 1,000/=.

R3: As for me; I don’t watch TV much, but when my friends in town who have smartphones tell me about foods they see on Facebook or TikTok, I also feel like trying them.

R1: Now that I ride a boda-boda, I eat foods that fill me fast so I can continue without feeling hungry.

**M: What challenges are faced by the youth in wanting to eat a healthy diet?**

R1: The challenges they get I think for the youth to behave what they are not it may trigger them to start stealing in order to get for themselves what they want.

R4: Some youths don’t want others to be better than them and they want the girls to be seeing them as smart ones and even the healthy eating and it can lead to some of them starting to steal like he has said.

R3: We boys can eat *emamba* [lunch fish] freely, but girls are not allowed. For us, it makes us strong, but for them it is a taboo.

**M: So now if you are there and you want to… we termed healthy eating as eating carrots, greens, apples and then others so what would hinder you from eating those things even if you wanted to but you cannot access the healthy diet that you want?**

R6: It can be sickness and then the behavior of a person.

R2: Sometimes when you don’t have a job you cannot afford to get a healthy diet.

R4: Even the thing of having where you want to grow crops from but then when you are tired and you don’t have the energy to do that and then you don’t have seeds to plant and get the desired food.

R7: The other person can want to grow crops but then when they don’t have any piece of land where they can do that.

**M: In your opinion, what comes to your mind when you hear about the dietary habits of young people at home?**

R7: They eat posho, rice, Matooke and sometimes they don’t want to grow crops especially Matooke because they fear they take long to get ready for harvest.

R3: They eat guavas, jack fruit and everything that is why you see…

**M: Now those who have money, which food items do they usually buy if they have some money?**

R1: They mostly buy chicken, egg roll, chips, yoghurt you know those expensive things and for these youths even if they get some 10,000/= in their pocket they buy those things and it is what they like.

R7: Bond 7, X5 gin, yoghurt, power play, predator then also they enjoy playing lotto with those machines.

**M: Are there youths who can get money and they buy greens for themselves?**

R7: No, the only greens they buy is mairungi they buy it from ghetto…

**M: Where do they buy these egg rolls?**

R: On the road side there in Mayuge.

**M: Now for you all who are here if you have money what do you buy?**

R3: For me if I have money I go to the stall there and I make a pizza then I go and get myself a power play.

R7: I get a piece of chicken and then also get yoghurt then I feel big in the city.

R4: For me I buy chapatti and a soda then I take.

R1: When I get money I go and buy chips and Oner then I eat and feel happy.

R2: For me I buy chicken and milk.

R: For me I buy soda because all the rest we do eat them always.

R6: For me I buy some meat there and then soda.

**M: So what factors influence your food choices as youths that you go and buy those different food items?**

R2: Money is the number one factor.

R3: Then also you can be so greedy then you go and buy that thing and eat.

R6: It depends there can be youths who are proud and they feel themselves so they can feel that peer influence.

R1: For me I think for us the youths when you make money there is that thing that pushes you to spend it so you just have to fight that because even if you don’t have appetite but because of the money you have you want to show people that you can buy those things and you eat well.

**M: What do you think of the various food items youths buy or that you buy?**

R7: I think you just feel proud because then also someone could buy it and you couldn’t afford it so it becomes your chance to also brag on them and luckily enough at that time they didn’t have money so you feel good on them.

R1: Youths like girls and so when you see your friend shopping something for their girlfriend you can also go and do the same for yours.

**M: Do you all think that when you are having those food items do you consider it healthy eating or not?**

R5: I think it is unhealthy because those are the things that cause sickness in our bodies.

R3: We just eat those but they are not good stuff because they have chemicals that go into the body and then weaken the body hence exposing it to sickness but we just eat.

**M: What makes you eat those food items even when you know they are not healthy items?**

R5: It depends on the current situation that you are in…

R: Situation like when this one has or this one doesn’t have there is that money that comes by impulse and then you find me you friend I can give you money and because they did not expect that they go and have something to eat.

R6: You might find your friend eating what you think it is bad and he also buys for you and because you don’t want to disappoint him you also eat but still it can be sweet for you.

**M: Are there nutrition programs that target youths here in your community?**

R2: No, we have never seen those.

**M: But would you like to have those programs and which ones in particular would like to have here in your community?**

R2: I think if there could be a program to train us the youths about catering so that we can learn how to cook and then we could start cooking on functions…

R4: For me I have ever gotten a catering job and I work so on the weekend when we get a gig we go and work.

R7: Even us we can cook that good food and then people just eat it.

**M: How do you want that catering program to be done?**

R: It is best that we start doing it from the community as the youths and then from here to the whole country.

**M: What age bracket do you want this program to cover?**

R: I think it should be from 18-25 years like there has to be an older person who can instruct these ones that you have put more firewood or you have to do this and that…

**M: What challenges do you think might affect this program?**

R: There are haters that can come and try to ruin our work they can even put poison so over controlling people to enter the program.

**M: Is there any other program that you would like?**

R4: Maybe they could come and put for us a program to teach us vocational skills and training…

R5: They should come and train us on farming activities so those also can help us…

**M: What challenges can be involved in the farming activities program?**

R: I think getting fertilizers, spraying to avoid pests, preparing the land for cultivation… and all that.

**M: I think we have reached the end of our discussion.**

**FGD_Girls_10-14yrs_ In school_June_2024**

**DEMOGRAPHICS**

| **Participants number** | **1** | **2** | **3** | **4** | **5** | **6** | **7** | **8** |
| --- | --- | --- | --- | --- | --- | --- | --- | --- |
| **Household size: Participants should have different household sizes (those from small and large household sizes should be prioritized)- insert number of people** | 08 | 05 | 12 | 06 | 14 | 13 | 05 | 07 |
| **Household heads: Participants should have different household heads (A- single parent household, B- two-parent household, or C-other guardians)** | A | A | B | B | C | A | C | C |
| **Marital status**   1. **Single never married** 2. **Married/staying with partner** 3. **Separated/divorced** 4. **Widowed** | A | A | A | A | A | A | A | A |
| **Disability (A-Yes ; B-No)** | B | B | B | B | B | B | B | B |
| **Subjective SES: A-above average ; B-Average ; C-below average** | B | B | B | B | B | B | B | B |
| **Religion: Participants should have different religions** | Participants represented different religions (catholic, protestant, born again, Muslim and others) | | | | | | | |

**Moderator (M):**

**June, 2024. Time is 3.07pm.**

**M: Let’s start.**

**What comes to your mind when you hear the word healthy?**

R1: Healthy means a situation where one stays in a clean environment and uses clean things to eat food in order to avoid diseases.

R7: Healthy means growing up well without any distances.

R5: Healthy means being well without diseases, sleeping in a good place and clean environment.

**M: Ok. Who else?**

**M: So what shapes your understanding of being healthy?**

R8: When I go to the hospital and Iam told that i don’t have any infections, I will know that Iam healthy.

**M: Ok.**

R1: You get to know from your body that you are healthy when you look good.

R6: When Iam able to walk I will know that Iam healthy.

R5: When you go for regular checkups you will know that you are healthy.

R2: I get to know that someone feels energy on the body.

**M: Thanks**.

**So what does a healthy diet mean to you?**

R3: A healthy diet to me is when you eat proteins, vitamins, vegetables, greens and water.

**M: Ok.**

R1: To me it will have proteins and carbohydrates.

**M: Ok. Who else.**

**Tell us what you think.**

R3: here at school we eat beans then at home we eat fish, milk, and egg. That’s a healthy diet to me.

**M: Ok.**

**So factors influence your food and drink choices?**

R4: Iam influenced to pick that food in order to avoid diseases.

**M: Like which foods?**

R4: Like fish

R1: What influences me to eat posho is that it gives me energy.

R3: Whenever I get the opportunity, I cook greens because I was told by a community health worker it helps to prevent diseases.

R1: I am influenced by the information I have that when I eat beans, I will get energy.

**M: Ok.**

**So what motivated you to eat healthy diets?**

R5: I get motivated because proteins give me energy.

R9: I also want my body to have proteins and vitamins which helps in fighting diseases.

**M: Ok.**

R8: I am motivated because I want to grow and get energy. I also want my body to look nice and sexually appealing, so I have to eat sweet things, which helps me gain weight and improve skin colour to look good.

R4: I eat greens because they will help me get enough blood.

R3: I always hear my parents being counseld at the health center, they were told to stop eating sugary things and that’s why Iam motivated to drink water.

**M: What challenges do you face when it comes to eating a healthy diet?**

R3: lack of finances to buy those healthy foods.

R2: Sometimes I want to eat healthy but don’t have appetite due to sickness.

R7: Sometimes we just lack appetite eve if we are not sick.

R6: lack of foods limits us from getting healthy foods.

R2: Some of those healthy foods smell badly for me, like greens (i.e amaranth leaves, cabbage..). That’s why I fail to eat them.

**M: Let us discuss your surroundings when it comes to food.**

**What is your opinion on the dietary habits of young people in your household and community?**

R8: Young girls our age like eating rice.

**M: Ok.**

R7: Most young people like eating matoke and meat.

R6: They prefer eating rice and meat.

R6: The like eating foods that give them vitamins.

**M: Like which ones?**

R6: like greens, avocado and fruits.

R1: On my side, I prefer eating millet bread and fish.

R3: Most young people prefer proteins like fish meat.

**M: What about drinks?**

R4: They prefer milk and soda.

R2: They also prefer taking packed juice like u-fresh.

**M: What about girls in particular what special hobbits do they have?**

R3: Girls are picky and they want only sweet things.

R2: Girls like copying what their peers are eating. When my friends have pocket money, they buy fried cassava and pancakes. If I don’t also buy, they laugh at me

R1: Girls eat what is provided to them.

**M: What food and drinks do vendors in your community and school sell?**

R4: They sell coated cassava.

R2: The sell rice balls and samosa.

R7: They sell stick ice.

R5: They sell mandazi and chapati.

R6: I always see them selling pancakes .whether salty or banana pancakes.

R8: They usually sell mwezinge (fried soya snack)

**M: Thank you so much.**

**What food and drinks do you typically buy from food vendors?**

R2: I usually buy fried coated cassava.

R6: I usually buy pan cakes.

R7: I always buy rice balls

R4: I usually buy rice balls and samosas.

R3: I buy rice samosas and salty pancakes because they are cheaper.

R1: I usually buy my favorite which is mandazi.

**M: What factors influence you to purchase food and drinks sold by food vendors?**

R1: What influences me to buy from them is the fact that the one I buy from is really clean.

R2: What influences me is hunger.

**M: Ok**.

R3: I buy samosas from a vendor who is clean and has dry containers.

R4: Iam influenced by the size of the food. If one cuts big cassava, that’s when I buy.

R7: When I see my favorite artist eating something, I feel like I want to try it too.

**M: Ok.**

**What are your opinions, as well as those of young people in your household or community, about food and drinks sold by food vendors?**

R2: I always think that what we buy is clean.

R3: I think that most of these food vendors are earning a living through food vending because they have children like us to take care of/

R7: Many of them have many children and they bring their foods here so that they make money.

**M: Any other?**

**If none lets go on.**

**Now I will ask you about nutrition related health programs**

**Have you ever received any nutrition or food related assistance or program in school**

r3: what we have is a demo garde where we plat our vegetabkes , cassava e tomatoes, matoke etc. on staurday we always getcassava that they cook for us..

m: ay other program wether I the community?

r1: our mothers teach us how to cook ad that’s what I kow.

M: So has the availability of a school garden influenced your dietary or lifestyle behaviors?

R5: There are students who have learnt how to farm some of them dint know. They now do it at home.

R4: We have benefitted from vegetables and cassava.

**M: Does the school teach you nutrition related topics?**

R4: Yes they do.

**M: Ok.**

**M: So what recommendations would you want to see happening at school and in the community?**

R3: I suggest that this year, the school should cook for p.7s matoke and beans on Thursdays.

**M: What about programs?**

R1: They should teach us how to plant vegetables so that we do it at home.

**M: How should it be designed?**

R2: We should have a group of atleast 6 to 10 students in a group.

**M: Who should be involved?**

R7: Those from 10 to 16 years.

**M: What challenges do you anticipate?**

R5: Some members might spoil the seeds when planting wrongly.

**M: What can be done there?**

R7: We need proper skills of planting in a row.

**M: Thanks so much for participating in this meeting.**

**FGD_Girls_15-19YRS_Out of school_June_2024**

**DEMOGRAPHICS**

| **Participants number** | **1** | **2** | **3** | **4** | **5** | **6** | **7** | **8** |
| --- | --- | --- | --- | --- | --- | --- | --- | --- |
| **Household size: Participants should have different household sizes (those from small and large household sizes should be prioritized)- insert number of people** | 09 | 08 | 7 | 6 | 5 | 5 | 6 | 4 |
| **Household heads: Participants should have different household heads (A- single parent household, B- two-parent household, or C-other guardians)** | B | B | B | A | A | B | C | C |
| **Marital status**   1. **Single never married** 2. **Married/staying with partner** 3. **Separated/divorced** 4. **Widowed** | A | A | A | A | A | A | A | A |
| **Disability (A-Yes ; B-No)** | B | B | B | B | B | B | B | B |
| **Subjective SES: A-above average ; B-Average ; C-below average** | B | B | B | B | B | B | B | B |
| **Religion: Participants should have different religions** | Participants represented different religions (catholic, protestant, born again, Muslim and others) | | | | | | | |

**Moderator (M)**

**Time check is 16:40 PM**

M: **What comes to your mind when you hear the word healthy?**

R1: Healthy to me means eating and sleeping well.

R2: Healthy means eating well without anything disturbing you.

R3: To me, healthy means that you are physically fit. You don’t have any problems and you eat well.

R4: Healthy means living a life where you do not frequently fall sick.

R5: Healthy life means living a stress free life.

**M: OK**

R6: To me healthy means living a life full of happiness.

R7: A good health is when your parents do not mistreat you.

R8: Healthy means living in a good environment where no one mistreats you.

**M: ok**

So **what has shaped your understanding of having a healthy life?**

**How did you get to know?**

R8: What shaped my understanding is the way I am treated, the type of place I live in, it makes me know that I live healthy.

**M: ok**

**You told me that healthy means eating well, being treated well. Etc. what shaped your understanding?**

R7: I understand that through seeing that someone is eating well.

R4: As for me, I talked about mistreatment because one time someone treated me so bad.

**M What happened?**

R4: This person mistreated me and used to ask me, don’t you have a place you came from.

They also used to buy food and give us leftovers that has remained on the plates or some time refuse to give us good food.

That’s what made me know that I am in a bad health.

**M: Is that still going on?**

R4: No I am now back with my mother, and life is okay.

**M: ok**

R5: When I stay somewhere and I’m treated well, I will know that I am living a healthy life.

**M: Treated well how.**

R5: For instance, if I am not treated as a kid, not being ruled all the time like a kid.

**M: ok, meaning you are now grown up. You don’t want to be taken as a kid.**

R2: What shaped my understanding of healthy living is when I saw that I eat a balanced diet, I get medical care.

R3: To live healthy is when no one mistreats you. You are taken as a child of the family and not over being led like a child, and not being beaten.

**M: Are girls your categories still beaten?**

R3: Yes

**M: I thought it ended in school.**

R8: We are still being beaten.

R1: To me, when I get little food, I won’t be healthy

**M: Don’t you speak when you are not satisfied.**

R1: No one cares.

**M: Now I would like to know more about your dietary habits. Please tell me about your dietary habits and what does a healthy diet mean to you?**

R7: As long as I eat chicken, I will take that as healthy eating.

**M: Which kind of chicken?**

R7: Whether local chicken stew or fried chicken then steamed matoke, then if I eat rice and beef.

**M: So that’s your healthy diet.**

R7: Yeah and if I eat that kind of food I really feel peace in my heart.

R1: To me a healthy diet means that I eat breakfast, lunch and supper.

**M: Give us examples of food items in those diets.**

R1: for instance if I take tea, bread, fruits like jackfruit, pawpaw, food like sweet potatoes, beans and rice

R3: The foods I eat and consider a healthy diet to me are spaghetti, cassava and sweet potatoes,

**M: What about sauce?**

R3: With sauce I prefer eating beef and fish.

R6: A healthy diet means eating matoke, yoghurt, milk, sweet potatoes avocado, greens.

R5: To me it’s eating food like posho, fish and meat

R7: What I call a healthy diet is when I eat posho and beans.

R1: What I call a healthy diet is when I eat posho, fish and drink sodas.

**M: Ok.**

**So what motivates you to eat a healthy diet?**

R1: The way the food was prepared can motivate me to eat it.

R2: What motivate me is that I want to eat what helps my body to have energy.

R4: What motivates me is the food that appeals my eyes whether well prepared or not. Sometimes my friends talk about the foods celebrities eat, like fried chicken or pizza. Even if I haven’t seen a pizza myself, but wish to taste it one day.

R8: What motivates is the food aroma.

R6: I get motivated to eat healthy food because I get satisfied.

R5: For me as a girl, what motivates me to eat a particular type of food is the desire to eat what helps my body to have energy to do my daily work and to also grow very well.

**M: Ok.**

R5: To me healthy food has good aroma that motivates me to eat it.

**M: Than what are the challenges that you face when it comes to eating a healthy diet?**

R8: Sometimes you may want to eat healthy but lack the finances to buy.

R7: Sometimes the healthy food is not easily available and you just have to admire.

R4: Sometimes our caretakers have the ability to buy those foods but just don’t want to them yet you really desire to have them but you don’t have the money.

R1: As a girl, since I was young, my grandmother told me never to eat emamba. Even now at my age, I can’t try it because people will say I disobeyed our culture, even though I know it gives energy.

**M: Ok, what about the others.**

R6: Sometimes you may be having a child who needs to eat ad dress but afterwards the child falls sick. You cannot go and buy food stuffs when your child is sick

R5: The same applies to school fees issues. One cannot buy foodstuffs when your child has been chased for school fees. You have to first clear fees before you deal with food issues.

**M: Apart from looking at expensive things like chicken, someone mentioned about eating greens as a healthy food. What challenges do you face when you want to eat such foods?**

R2: When you are staying in the town centers, you can’t have access to farm land and can’t do it at home because you stay in a squeezed place.

R7: When I eat greens especially dodo (amaranths) the whole body itches me seriously and leaves me with spots.

**M: What happens who you eat other types of greens.**

R7: I don’t get issues when eat the other types. That also includes taking milk. When I take milk i vomit the whole night and lose sleep. So I fear eating some foods or dairy products because of that.

R8: Sometimes we are told that we have hand that destroys crops. You might be having greens in the neighborhood where you can pick from but the moment they see you picking, they start their levying their accuses on you that you are the one who made those greens dry out.

**M: Drying them how?**

R8: It means that whenever you pick something in the garden, the crops dry immediately. So they call you a curse and a crop destroyer. Meaning you can’t easily get those greens.

**M: Eeh. Do they sometimes put the blame on the hot season?**

R3: No they put the blame on you.

**M: Ok.**

R2: Sometimes I may want to buy those greens but lack money to do so but at times when I eat such foods I don’t feel comfortable.

**M: Let’s discuss your surroundings when it comes to foods.**

**What is your opinion on dietary habits of young people in your households and community?**

R2: They mostly like eating rice and they eat too much.

**M: Ok.**

R4: They like eating rice, posho ad beans

**M: Why do they prefer such foods?**

R4: They prefer such foods because it makes them full for a while and rice is a delicacy for most young people.

r5: they like eating katogo.

**M: Which katogo?**

R5: Cassava mixed with beans.

R2: They mostly like eating sweet potatoes because it’s always cooked in plenty so when they eat at lunch, they can pick it from the saucepan several times.

**M: Do you mean that young people don’t get satisfied easily?**

ALL: Yes.

**M: Is it the girls or the boys?**

ALL: Both girls and boys.

R3: There are some girls who really eat a lot. They want to eat double the portion they have been served.

R2: Not all the time. Sometimes we go with what we have been served.

R7: As for me I want to eat 3 plates of food at a go.

R2: Ooooh

R6: I can eat 2 plates of food. And I feel like eating all the time.

**M: So do the girls like eating snacks as well?**

R4: When I have my money, I go and buy snacks as many ties as I can afford.

R7: Sometimes when we don’t have money for buying snacks, we end up picking sweet potatoes several times from the saucepan so that we feel satisfied.

R5: For us girls, when you eat foods that make you grow fat, people admire you and say you are beautiful, so I also try to eat such foods when I can.

R8: What you should know is that the biggest number of girls eat double plates of food and add snacks on top of that.

**M: Ok.**

**Let’s look at food vendors where you buy food from.**

**What food and drinks do vendors in your community sell?**

R1: They sell samosas, chapati, katogo (cassava and beans mix)

R2: The food vendors in my area sell fruits like jackfruit, passion juice, pineapples.

R3: They sell jackfruit. Mangoes, samosas and chapati.

R7: They sell fried chicken, chips and others.

**M: What others?**

R7: Rice balls, soya snacks and deep fried cassava chips.

R6: They mostly fry pancakes banana ripes, chapati

r5: some vendors sell Ebigodo (cooked cow hinds and head) cooked bananas and boiled cassava.

**M: Ok.**

**So what drinks and foods do you usually buy from the food vendors?**

R7: I buy rice balls, pancakes and samosas.

R4: I usually buy sugarcane, mangoes and fried cassava. When i buy those items I feel good.

R5: I buy pancakes, chapati, cakes and banana ripes.

r2: I usually buy chapati, soya snack and mangoes.

R6: I usually buy chips, sugarcane, jackfruit and avocado from food vendors.

**M: What about drinks?**

R6: I buy yoghurt.

R3: I buy chapati, pancakes and bananas.

R8: I buy sweets, sodas and avocado.

**M: What are your opinions and that of young people about food sold by food vendors?**

R6: I buy from a food vendor who has customer care is the one I want to buy from.

R4: The vendor I buy from should be very clean and has customer care. I can’t but food from a dirty place.

**M: Ok.**

**What about the others.**

R2: I always want to buy food that will make me satisfied and also give me energy.

R: My thoughts about that food is that I want to buy something that’s really delicious.

**M: Delicious like which foods?**

R3: Delicious like cakes, chapati and bean mix then sodas.

**M: Any other?**

**Ok.**

**M: Ok. Thank you so much for participating.**

**FGD_GIRLS_20-24 YRS_IN SCHOOL_June_2024**

| **Participants number** | **1** | **2** | **3** | **4** | **5** | **6** | **7** | **8** |
| --- | --- | --- | --- | --- | --- | --- | --- | --- |
| **Highest education Level attained (A-None B-Primary; C-Secondary, D-post secondary)** | C | C | C | B |  | B | B | B |
| **Household size: Participants should have different household sizes (those from small and large household sizes should be prioritized)- insert number of people** | 10 | 11 | 10 | 6 | 7 | 10 | 7 | 8 |
| **Marital status**   1. **Single never married** 2. **Married/staying with partner** 3. **Separated/divorced** 4. **Widowed** | A | A | A | A | A | A | A | A |
| **Disability (A-Yes ; B-No)** | B | B | A |  |  |  |  |  |
| **Subjective SES: A-above average ; B-Average ; C-below average** | B | B | C | C | C | C | C | C |
| **Religion: Participants should have different religions (A-Catholic; B-Anglican; C-Born Again; D-Pentecostal; F-Muslim)** | Participants represented different religions (catholic, protestant, born again, Muslim and others) | | | | | | | |

**Moderator (M)**

**Transcript**

**M: Thank you for giving me this chance to speak to you and like I have introduced myself to you, you are also going to introduce yourselves but I think we shall do that at the end. So my first question is what do girls in this community enjoy most?**

R1: I mostly enjoy…

**M: Now for me let me tell you for me I enjoy football, going out and music…**

R6: I enjoy netball and listening to music.

R8: For me with music and netball…

R7: I like reading and music.

R2: I enjoy outing, going to club, swimming and then music.

R4: I mostly enjoy music, parties and then outings.

R3: I mostly listen to music and dancing.

R1: I mostly enjoy watching movies and when we are at school I enjoy playing football then dancing, reading and also movies.

**M: Now we are going to talk about being healthy, what comes to your mind when I talk about being healthy?**

R1: For me to be healthy I have to eat food that gives me strength and when it builds the body and it is well cooked then another thing I have to be showering and also drinking water.

R5: I think I should be clean and wash my clothes, I have to eat food that is prepared very well and then I have to drink enough water.

R8: When I hear a thing about that I have to be clean and the food I eat I have to be eating I in time no these things of eating half of it and then the flies come and start from where I have stopped.

R3: If I want to be healthy I have to be healthy I need to eat good food like rice, Matooke and then the sauce should be meat…

R4: For me I have to be clean and even in the place where I am at it is clean then also to drink water.

R2: I have to be clean then also to drink boiled water, I have to eat well cooked food and then also enjoying life.

R6: Being healthy you have to be alive and then you are clean and then the food you eat is also good and healthy.

**M: Okay, what has helped you to source out or to know what a healthy person should be like?**

R1: For a person to be healthy still they be there when they are strong and without stress then also they don’t have sickness with them you can literally see their body when they are healthy.

**M: How did you get to know that?**

R1: I studied and got to know about that in school.

R: Even me at the school where I was studying is where I got to know about being healthy and even how you feel you can know if you are sick or not and even at home the parents also tell you that you have to do this and this for you to be healthy… you have to wash your clothes, you have to eat food in time, you have to drink boiled water.

**M: Now we are going to talk about nutrition and healthy eating, what does healthy eating or eating a healthy diet mean to you?**

R2: For us we think that eating well is eating rice, meat, chicken, macron like how you know things of nice people so they forget that even when you eat greens is also healthy eating and you are adding nutrients to your body.

R: Healthy eating… like how she has said that we think of eating pilau, meat and we forget that you might be eating pilau when for me I am eating greens then I make a good skin better that yours who is eating pilau.

R6: Healthy eating is not eating good stuff it depends on how you are eating now like for us in the village people who see us eating greens they think we are eating bad things those who are eating meat and the other nice things but still I think we are doing better because for meat you have to fry yet for greens it is just boiled with no oil and the nutrients…

R1: Eating healthily… others think that eating well and healthily okay for us we eat meat, rice, Matooke, fish are the good things but then for a healthy person to me they have to eat some greens, jobyo, garden eggs… why I say so is that because you will eat meat which brings sickness and then for me I will have my greens which make me feel healthy and cures some of the sicknesses within the body.

**M: What influences your food choices as youths in this community?**

R5: I think it is when the heart desires to have and eat something, when it doesn’t want then I cannot eat that thing…

R6: For me to choose to eat something it is the heart and the appetite because I cannot force myself to eat a thing when I actually don’t like it and then you have to have something that you can afford you cannot put yourself on something that you cannot afford like if you cannot afford meat then you cannot want to eat it.

R2: Now some of us are still in our parents’ house and then the parent will not go ahead to buy greens and then for you just because you have seen your other friends eating meat then you think that you will also eat meat so you have to eat what is available so anything available is what we go with.

**M: Okay, what else we have seen the heart’s desires, parents, and then what else influences what you will eat or choose to eat… and tell me is there anyone who eats something because they have seen others eating it?**

R: Not really because you can find that I am eating meat and yet this one does not eat meat and so the other one cannot eat that because they have seen me eating it…

R: There are people who do eat something because they have seen other people eating it so they go and buy that particular thing and they eat it that when they are admiring you and so because of that they have to buy that thing and they eat it.

**M: But what motivates people to eat what they do eat?**

R5: You be admiring to eat that particular thing.

**M: Is there any other thing?**

R5 Others do because of the appetite… then others it is just for them to be seen so that others can admire them and so that motivates them when they see their friend eating chicken they also go and buy it.

**M: Now, what challenges do youths of your age face when they want to eat healthily because you already told me what you think a healthy diet is so what challenges do you face when trying to have a healthy diet?**

R6: The challenges we face when trying to eat a healthy diet is lack of money and you will not go to your parent because they will ask you that… sometimes the parents are harsh and sometimes the others in case they listen they might not be in position to help or others can buy but at times they don’t have money and you could also see it for yourself.

R2: They be not having money and yet they cannot even go and steal even if they go and steal they will cut them with a panga.

R5: Now for us girls you can admire something when you don’t have money that is when you go to a boy and they lie to you and give you money and before you actually get know it you are pregnant just because you wanted to eat the other thing that you wanted.

R3: There are those young children who you can send for things to eat and then when they are bringing them to you they first take some of it before they bring it to you…

**M: Why is that so…? Okay let us go ahead so we have looked at the things regarding nutrition around you so what opinions do you have about the dietary habits and practices of young people in terms of food at home?**

R1: I think when we are preparing to eat… you have to first eat and after eating you eat when you have kept quiet and then before you eat you first pray. The other thing is that you have to eat as you drink water to avoid food from choking you.

**M: Thank you so much. I am repeating the question for those who may not have understood that when time comes for food how do girls here behave either here at school or at home?**

R5: Now girls of our age we have to… you cannot go for food when you have not washed your hands so you have to eat food with clean hands and then you have to eat when you are seated down and then you don’t have to talk while eating.

R8: When you are going to eat you have to first go and get water then go and eat food then after you pray then you eat and then after eating you wash the plate.

R6: When I am eating I don’t eat so fast because when I add speed that I eat so fast then I get stomach complications so I have to eat slowly and that is for me…

**M: Now let me ask another… are there shops and the canteen here at school and I intend to ask that what do girls mostly eat from there like the eats and the drinks?**

R1: They don’t usually go to the shops but they cook some *“mondi”* so you see it is one person who has gone but they want to finish the whole bucket.

R2: For us we mostly eat chapatti so you can eat chapatti until when you feel like wheat is filled in your stomach intestines and then samosas we eat them and feel like we don’t want to stop like when we are enjoying.

R7: We mostly eat rolex and soda…

**M: So you have talked about *mondi*, chapatti, samosas, mandaazi, cassava… is this fired cassava or not?**

R6: The fried one.

**M: Is this the one they call chips?**

R: It is two types the one for cassava and for Irish potatoes but me I enjoy the one of Irish potatoes.

**M: Okay, so what about the drinks you have mentioned soda… what else?**

R3: There is U-fresh juice.

R2: For me I mostly like yoghurt and Stoney.

R5: For me I like the passion fruit juice.

R1: For me I like Oner most.

R6: For me I enjoy Yacket juice.

**M: So now what influences your choice to choose those types of foods and drinks that you eat mostly?**

R8: The thing that causes it is the admiring and then also having some money.

R7: It is the heart that triggers me to want to eat what I want…

**M: What is your opinion on the things sold because we have seen they sell samosas, chips and soda so what do you think about these things?**

R2: For me I might be hungry yet my friend over there is selling a thing also they want to get money and earn a living so I cannot allow to stay hungry when my friend is selling a thing that I want to eat and they themselves are looking for a living so by all means I have to go and buy.

R8: For me I cannot be hungry when the person selling eats is my friend so what I do I go there and talk to them and if they can offer me something to eat or lend me something to eat then I pay back later.

**M: Do you think those eats that girls usually eat are they good or not?**

R5: For me I think they could be good because they help us sometimes in our bodies.

R6: The eats that they tell us be very good…

**M: Why do you say so?**

R6: It is because someone cannot go and get something bad and then they come and sell it so they have to put good things to attract customers and if they bring something bad then it means they will not get customers.

**Thank you for participating**

**FGD_Parents_Female_June_2024**

| **Participants number** | **1** | **2** | **3** | **4** | **5** | **6** | **7** | **8** |
| --- | --- | --- | --- | --- | --- | --- | --- | --- |
| **Age** | 39 | 34 | 45 | 29 | 49 | 31 | 28 | 52 |
| **Highest education Level attained (A-None B-Primary; C-Secondary, D-post secondary)** | A | B | A | B | B | B | B | B |
| **Household size: (insert number of people)** | 9 | 4 | 10 | 5 | 8 | 4 | 8 | 9 |
| **Marital status**   1. **Single never married** 2. **Married/staying with partner** 3. **Separated/divorced** 4. **Widowed** | B | B | C | B | B | B | B | B |
| **Disability (A-Yes ; B-No)** | B | B | B | B | B | B | B | B |
| **Subjective Social Economic Status (SES): A-above average ; B-Average ; C-below average** |  |  | B | B | B | B | B | B |
| **Religion: Participants should have different religions (A-Catholic; B-Anglican; C-Born Again; D-Pentecostal; F-Muslim)** | Participants represented different religions (catholic, protestant, born again, Muslim and others) | | | | | | | |

Venue: XXX

This is XXX. I am conducting an FGD with female parents on the Arise Nutrint study In Mayuge district. The time is 12:43hrs.

**M: What are the norms and values related to cooking and dietary habits in this community?**

R7: We have a different tribes in the community. Some take millet bread as their staple food while other its posho, other sweet potatoes. Then most foods are prepared using firewood.

R2: Most families in the community eat posho and beans. Others use firewood while others cook on a charcoal stove.

**M: Do they cook the beans boiled or fried?**

R2: It depends. If they get cooking oil they fry the beans.

R6: In the community people prefer eating cassava bread because it’s cheap. They also used to cook silver fish a lot until it was stopped at the lake.

R4: people cook cassava bread a lot because it doesn’t consume a lot of firewood. They also eat greens.

R5: The most common food In the villages is cassava which most families eat without any sauce if the greens are not there due to lack of rains.

R7: In my community people eat potatoes a lot .they like it because you can eat it even if you don’t have sauce.

R8: where I stay, people use sweet potatoes and chwada (cassava bread) that’s the easiest food you can get during the dry spell like this one.

**M: What constitutes healthy eating habits in your and your family’s daily life?**

R3: to me it means balancing foods where I can get chwada and silverfish. That means I will be able to get carbohydrates and proteins but we still don’t have to eat the same foods every day. It’s important to keep changing.

R1: To me healthy means eating food and fruits, making well cooked food and ripe fruits that are suitable for eating. .

**M: Ok.**

R5: To me it’s eating food that is body building. .

**M: Like which food?**

R5: Like posho. Then you add fruits like pineapples that gives you vitamins.

**M: Is that what you eat at home?**

R5: That’s what I eat at my home.

**M: Ok.**

**Some of you mentioned energy giving foods and others mentioned body building foods, please tell us the difference.**

R7: Those foods have classes. For instance if you eat posho and rice for lunch, there won’t be any difference. It’s better if you eat posho and beans then you change to matoke and silver fish. There you will also get proteins as well.

**M: Ok.**

**So what challenges do you and your family face when it comes to eating a healthy diet?**

R3: There are people who know what a healthy diet is but the money to buy these food items may not be there and you will find such families eating one meal a day or sleeping without food.

R5: Sometimes I admire fish but find myself buying 10 kilos of cassava flour because fish is expensive. So we really want to eat a balanced diet and change foods but have financial constraints.

**M: Any other challenge?**

R1: People want to plant food but have no land to plant.

**M: What brought that?**

R1: The land owners took it away for sugarcane plantation. We used to hire land and farm but nowadays even where to plant sweet potatoes is a problem.

**M: So what motivates you have when it comes to eating a healthy diet you were mentioning?**

R6: What motivates me is whenever I sell my farm produce I get some good money and make sure I give my family a treat of fish, fruits , veggies or eggs.

R4: What motivates me is the season. When time for harvesting coffee comes, I sell and make sure my family eats a balanced diet.

R2: Unfortunately this season isn’t good because plants have dried up and we don’t know whether we shall get anything.

**M: So it hasn't rained here?.**

R8; It hast rained for a while.

**M: Ok.**

**Now we would like to discuss the eating habits of young people in this community.**

**What do young people typically prefer to eat on a daily basis?**

R2: Those teenagers prefer eating rice.

**M: Boil or fried?**

R2: Whether boiled or fried, as long as it's rice.

R4: The young people in my community want to eat rice day and night. They don’t get tired of it.

R6: At home children ask me whether I have cooked fish on specific days. If I haven’t, they eat but are unhappy.

R3: During dry seasons, we mostly have cassava bread and sweet potatoes. Those are the easiest foods to get and that is what they [AYAs] eat.

R8: Young people prefer eating Rolex, chapati, sodas, and energy drinks.

R5: If you prepare chips and chicken they enjoy it more than matoke and ground nuts. Then they also prefer eating fried eggs and fried roadside chicken.

R1: young people also like katogo.

**M: Which katogo?**

R1 Cassava and beans.

**M: Today’s young people also prefer to drink sweet and sugary beverages.**

**Is that true?**

All: it’s true.

**M: What do you think about that?**

R4: Most youths like drinks like kombucha and sweet drinks because they were told that it will help them erect.

R3: Young people like taking sugary drinks because they want sweet things and this has brought problems for girls because men use it as a bait to get them. They know girls love sodas.

R2: We always tell the young people that sweet drinks and sugary foods like chocolates are not good for their teeth but they insist.

R3: Most young people prefer eating rolex, plain chapati, sodas, and energy drinks.

**M: Ok.**

**How does the presence of food vendors influence the dietary habits of young people?**

R2: Food vendors have caused children to become uncontrollable because they now do odd jobs so that they get money to buy eats from vendors and they no longer want to do house work because the most common punishment is denying them home food which doesn’t affect them since they make their own money.

R2: food vendors are relatively cheap so if a child has UGX 500, he rather goes and buys katogo from the vendor.

R3: Children have learnt how to steal to the extent that they are now stealing from their friends in order to eat from the food vendors.

R5: Some girls are involved in early sexual debut because they want money for snacks and don't want to feel out of place at break time.

R7: Some of the kids don’t reach school. They change their uniforms on their way and go for odd jobs so that they can get money because of peer pressure.

**M: Do food vendors sell healthy foods or**….?

R5: They don’t sell healthy foods. Some of the foods are decayed, some are half cooked and some are very dirty. They take it for granted because they know that children have to buy. When you take a glance of where some of them cook from, it’s very disgusting, comes with dirty plates and places food in a very bad environment.

R6: Those selling mulokoni (cooked cow legs) always take back home the remaining pot then they warm the next day and bring it when flies have fallen in the sauce. They use that opportunity because they usually sell it at night and in a dark place.

R8: Most of those vendors are dirty. One time a lady selling mulokoni didn’t cover and she brought a dead rat inside.

R4: These days they even do witchcraft to call for customers. They wake up in the morning and wash their hands or face inside the food. Others wash other parts and pour in the food to call for customers so those foods are dirty.

R2: Most times those vendors bring foods which fall in the same class, for instance one brings plain fried cassava, the other coated cassava and the other boiled cassava. They are all the same.

**M: What role do social institutions such as family or schools play in influencing the dietary habits of young people**?

**Let’s talk about schools.**

R2: Schools convince parents to pay something small so that their children can have porridge or a meal during lunch time most especially this applies to parents who can afford.

R1: Schools support the existence of vendors who come and sell eats to the students.

**M: Ok. What about families. What role do they play in influencing dietary habits in young people?**

R5: Families always try to change the diet so that these young people don’t lose morale or their appetite. Families know that at school the child eats posho and beans so they try to change the veggie, greens and fish, if finances allow.

R7: Families have people who work and whenever they get some money, they buy nutritious foods.

**M: Let us discuss some of the barriers and challenges to eating healthy among young people as we look at interventions.**

**M: What are the most significant barriers to young people eating healthy?**

R6: The most significant barrier is lack of nutrition awareness in the community.

**M: How can it be designed?**

R3: We can have radio awareness programs like radio Mayuge where we can get prime time and we talk. Then we can organize community meetings as well.

**M: Who should be involved?**

Churches and mosques, Learners, teachers.

**M: What would be the challenges?**

R7: Native attitudes of the students toward nutrition programs. When you mobilize them they start criticizing you and the program

**M: So what can be done?**

R2: We need self-help projects that create an income.

**M: Ok. Any last words on nutrition?**

R2: I suggest that the government creates channels that teach parents nationwide about healthy eating habits.

**M: Ok. Thank you so much my dear parents.**

**FGD-Parents_Male_June_2024**

| **Participants number** | **1** | **2** | **3** | **4** | **5** | **6** | **7** | **8** |
| --- | --- | --- | --- | --- | --- | --- | --- | --- |
| **Age** | 58 | 35 | 54 | 58 | 54 | 41 | 70 | 52 |
| **Highest education Level attained (A-None B-Primary; C-Secondary, D-post secondary)** | D | C | C | B | B | C | B | B |
| **Household size: (insert number of people)** | 8 | 4 | 5 | 6 | 10 | 6 | 6 | 6 |
| **Marital status**   1. **Single never married** 2. **Married/staying with partner** 3. **Separated/divorced** 4. **Widowed** | B | A | B | B | B | B | B | B |
| **Disability (A-Yes ; B-No)** | B | B | B | B | B | B | B | B |
| **Subjective SES: A-above average ; B-Average ; C-below average** | B | B | B | B | B | B | B | B |
| **Religion: Participants should have different religions (A-Catholic; B-Anglican; C-Born Again; D-Pentecostal; F-Muslim)** | Participants represented different religions (catholic, protestant, born again, Muslim and others) | | | | | | | |

**The venue is XX primary in a classroom.**

**Time is 12:56 pm**

**Let’s begin. Let us talk about the food habits and cooking practices in your household and community.**

**M: What are the norms and values related to cooking and dietary habits in this community?**

R1: Most people here, especially the young people like eating fried foods.

R7: Older people don’t fry their sauce. They just add ground nuts in the sauce and eat that way.

R4: People think that eating healthy is eating foods like rice, matoke, and they despise millet bread.

R4: Since there is a lot of poverty, they prefer cooking rice because one can eat rice without sauce.

R1: There is a lot of poverty in this village. One may want to buy meat but has no money. So what they do is, if they get greens it’s eaten as sauce.

R2: Due to poverty, if one fails to get fish, they instead eat silverfish.

**M: Ok.**

R8: Nowadays the pattern of food has changed. Previously people used to eat at least 3 meals a day but now they can eat only one meal a day. People no longer have choices on the food. They eat what is available.

Long time, we Basoga, were matoke and sweet potato eaters, but nowadays it's posho and beans or silver fish for a change.

R6: In this community, some foods are taken as medicine. For instance we give children silver fish if they get measles. Unfortunately, fishing for silver fish was stopped from our surrounding water bodies.

**M: Who stopped the fishing?**

R: The government stopped the fishing activity yet we were used to using silver fish as sauce when the season was dry like now when we can’t get greens. We used to buy silver fish of UGX 500 and it would be enough. Now we have to buy for UGX 5000 for just one meal!

**M: Sure**?

R1: Due to poverty, we are getting many diseases because we can’t eat what we are supposed to eat. We are used to the fact that we take maize to the maize meal for grinding but again we hear that the flour comes out with some metals or steel particles from the maize mills. We also like frying foods and don't feel good if we don’t. Then due to the process at the maize mill, we lose a lot of nutrients while milling the maize.

**M: Ok.**

**What constitutes healthy eating habits in your and your family’s** **daily life?**

R3: What constitutes a healthy eating habit, is when the family learns to farm and grow things like fruits, greens and also rear chicken so that they eat whenever they want.

R5: What helps us is that we farm and plant fruits and veggies.

**M: What do you plant?**

R: I plant veggies like cabbages, ground nuts and fruits like sugarcane.

R2: At my home we rear chicken and always eat eggs. This is a habit and all family members know that we have to have eggs at all times.

R7: Since there are financial challenges at times, we always plan ahead and stock foods. We also have a small garden for only greens where we get ours from.

R4: Most importantly is that I rear cows that give us milk for both selling and drinking. Having a cow is important because the money I get from the milk helps me to buy greens.

R5: What has helped me is that I plant food that I can also sell to support other issues and eat as well.

**M: What food did you plant?**

R6: Cassava and potatoes.

**M: What challenges do you and your family face when it comes to eating a healthy diet?**

R4: The first challenge is lack of money to buy some healthy foods like fish for your family. The prices keep soaring higher so I fail to buy these foods.

Rr6: The challenge we have is the big population of our families. We have big families, to the extent that even when you want to feed them with something healthy, you fail because of the cost implications.

R2: When we gave birth to many children, land became small for the family. It's no longer enough for planting a variety of food stuff.

R7: Nowadays there are so many diseases that we spend a lot of money on healthcare that would have supported the family in healthy eating.

R5: We have been having a challenge of cutting the prices of our farm produce. The more the prices go down, the more the financial challenges where we fail to buy for our families things like fish due to struggling with finances.

R6: Drought has been one of our main challenges. Just like now, we don’t have rains and many plants have dried up in our gardens including greens that would have been helpful at home. This season we are already afraid that our beans and maize will not yield.

R1: The changing seasons have also affected us. Sometimes we have a drought for long but when it rains, it rains heavily and destroys all the crops.

**M: So now what motivates you and your family when it comes to eating a healthy diet especially the times when you decide to eat healthy?**

R: I like those bitter greens mixed with ground nuts and I always dictate that we cook them at home even when I have money because it helps us to have nice skin and clear eyes.

**M: Ok.**

R5: There are times when we attended workshops and they told us that when we eat greens it helps us to get enough vitamins in our bodies so whether I have them grown at home or buy them, I always ensure we eat them.

**M: Ok.**

**Someone mentioned that in the village people think that eating greens is a sign of povert**y.

R5: That’s true because most people haven’t yet gotten a chance where they are educated on what to eat, the few of us who got that chance are the ones who know what to eat.

**M: Now we would like to discuss the eating habits of young people in this community.**

**What do young people typically prefer to eat on a daily basis?**

R8: Most of those children prefer eating rice, fruits, fish and matoke. They don’t want to hear you talking about silver fish, eggplants or greens. They even tell you that they can’t eat greens and only prefer eating fish.

**R6:** As parents, we usually prepare food based on what we have harvested or can afford to buy; and I always tell my children to learn to eat what is available.

**M: Ok.**

R1: In our community, these young people prefer eating fruits like mangoes, jackfruit. They eat what you cook but become happy when you buy meat and fish.

**M: What about food?**

R1: They eat all foods like rice. Cassava, potatoes, but prefer rice.

**M: You can as well tell us what they prefer to buy?**

R5: I’ve observed that they prefer eating snacks a lot because they like having something that they can eat all the time. Sometimes they leave food at home and still go to look for snacks like bagia, samosa, and biscuits. Then most times they dont like eating beans because they think that the only healthy sauce is meat.

**M: So they prefer eating snacks**.

R5: So much.

R7: I prepared my home well by planting fruits and they really like eating fruits.

R6: The taste of meat and fish is so good and convinces these children that they think eating things like greens, isn’t nice because they are also not aware of the benefits, but they also like eating fruits.

R2: My child often asks for foods they’ve seen on television. It’s hard to say no when they insist.

R4: I always see that my children like eating katogo (cassava and beans) with fruits. Unfortunately, nowadays fruits are very few and when it’s there it isn’t enough for them.

**M: You planted a few.**

R4: We planted a few but they are always stolen, especially when you leave them on the tree to ripen. We usually remove unripe jackfruit from the tree and keep it to ripen in the house because of thieves.

**M: Ooooh.**

R2: Those young people like eating sugary foods like sweets, ice, biscuits. They also like eating sugarcane, avocado, jackfruit and ripe banana. They only prefer eating cassava in the form of katogo ( cassava mixed with beans).

**M: Today’s young people also prefer to drink sweet and sugary beverages. What is your view about that**?

R6: They like buying some powder known as jolly-jus which they either lick or dilute with water the whole day. It even leaves them with colored mouths that we sometimes think they have taken sodas.

R8: Children like ice cream. Sodas, packed juices like U-fresh in addition to sweets.

**M: Why do you think they like them?**

R4: It’s because those drinks are sweet and young people like sweet things.

**M: Ok. Let’s now look at food vendors. Do you know who food vendors are?**

All: yes.

**M: Let me first ask this question, what foods do young people usually buy from food vendors**?

R2: At break time food vendors bring their baskets and children buy Omondi (rice balls) so much. Then in the community they like buying sodas and jolly jus.

R4: Those children like chapati so much. And for drinks, they really like jolly jus juice.

R3: They prefer buying sugarcane so that they can share it with their friends.

**M: What about at home?**

R1: They like buying kikomando (chapati and bean). They also like buying katogo (cassava mixed with beans).

R2: Most chapati sellers wake up at 5am to prepare chapati and their biggest clientele are the young people. They also buy fried chicken pieces a lot with soda or juice.

**M: As parents, How does the presence of food vendors influence the dietary habits of young people?**

R6: Children have started stealing because they want to buy edibles from vendors. Secondly, those edibles have made them drop out of school. They prefer to work in sugar cane plantations where they get money to buy food from the vendors.

R8: That food sold by food vendors is not clear, because we are not sure of how they prepared it since they come with already cooked food for sale.

R3: When vendors bring food before 10am, most children start feeling hungry even before break time just because of looking at the vendors who came early to school so it’s affecting them.

R4: Many children are also getting spoiled because they want to look for money to buy food from vendors.

**M: Let us discuss some of the barriers and challenges to eating healthy among young people. Although you have talked about so many challenges affecting healthy eating among young people, what can be done to promote healthy eating among young people**?

R2: Family members should get involved in the production of food so that we have plenty of food, greens and fruits. When we make an effort we shall get a big yield.

**M: Who should be involved?**

All: family members and Community members

**M: How should it be designed?**

R3: empowering all family members of the importance of producing healthy foods

**.M: What Challenges do you anticipate?**

R4: Animals that destroy our crops

R5: Lack of enough land. Lack of financial support

**M: What are the Solutions?**

R2: District extension workers should get involved and support in providing technical advice .

R6: Fencing school land to protect the crops from animals.

R5: Another intervention would be creating awareness about healthy eating

**M: How should it be designed?**

Invite the nutritionist’s and other technical people to carry out awareness programs

**M: Who should be involved?**

R1: CDO and community.

**M: What are the anticipated challenges?**

R2: Failing to facilitate the officers, Poor time keeping by community where they want to first go for farming

**M: Ok. Any other?**

**Thanks for participating in this focus group. The information you have provided has been very helpful. It will be used to help us to design interventions to promote healthy eating habits among young people. Are there any questions that I can answer before we end the session?**

**…………………**

**…………………**

**If none, thanks so much for your time.**

**FGD_Teachers_SecondarySchool_June_2024**

| **Participants number** | **1** | **2** | **3** | **4** | **5** | **6** | **7** | **8** |
| --- | --- | --- | --- | --- | --- | --- | --- | --- |
| **Age** | 51 | 32 | 28 | 23 | 32 | 33 | 30 | 21 |
| **Duration in teaching position** | 20 | 4 | 3 | 1 | 5 | 9 | 5 | 2 |
| **Household size: Participants should have different household sizes (those from small and large household sizes should be prioritized)- insert number of people** | 5 | 4 | 1 | 8 | 3 | 1 | 3 | 5 |
| **Marital status**   1. **Single never married** 2. **Married/staying with partner** 3. **Separated/divorced** 4. **Widowed** | B | B | A | A | B | A | A | A |
| **Disability (A-Yes ; B-No)** | B | B | B | B | B | B | B | B |
| **Subjective SES: A-above average ; B-Average ; C-below average** | B | B | B | B | B | B | B | B |
| **Religion: Participants should have different religions (A-Catholic; B-Anglican; C-Born Again; D-Pentecostal; F-Muslim)** | Participants represented different religions (catholic, protestant, born again, Muslim and others) | | | | | | | |

This is XXXX (CSO) conducting an FGD with teachers from secondary schools Mayuge district.

**M: To begin with, let us talk about the food habits and cooking practices in your household and community.**

**What are the norms and values related to cooking and dietary habits in this community?**

R6: As a person I like eating g-nuts sauce and greens. Children love rice so much but whenever I cook boil greens, they refuse to eat food because they want it fried. The same applies in the community. Most families like fried foods and whenever they cook it boil the children refuse to eat stating that it’s not tasty. But that’s the nature of children. Then at schools, students like posho and beans.

**M: What about drinks?**

R6: They prefer taking water because much as they like sodas, it’s expensive for them.

R1: I usually eat greens and millet bread. Then in the community, many people prefer eating matoke, and cooking with charcoal. Unfortunately, charcoal is now very expensive, most homes in the community also don’t cook lunch during schooldays. Then with for the students to come back then start preparing supper. These students sometimes go to schools where they don’t provide lunch, or the students missed lunch and get back home when he is angry and just waiting for supper. That’s why they get back home when they are in low moods and don’t want to participate in any household chores.

At schools, the students eat posho although sometimes it’s not good quality and doesn’t not well cooked. That food is not fries yet students want fried beans. We can’t fulfill that all the time because their parents have no money to facilitate that all the time.

R6: At school children eat posho and beans but they change diets on weekends,

**M: Thanks.**

**Please tell us about the sauce they eat.**

R4: They like fish so much. Whenever you cook g-nut sauce, they will tell you that you didn’t cook well. Then they also like milk and water.

R7: Day scholars usually eat posho and beans including the teachers although the teachers eat beans which are fried and have a thick soup than for the students. Sometimes we have greens which are for those who don’t eat beans due to ulcers, though once in a while even those who eat beans get to eat greens. Then in the midweek, they eat fried brown rice.

The boarding students rarely change a meal and if they are to change diet, they will eat rice and greens on a weekend or silver fish and rice, there are students who buy from the vendor’s food like cabbage and rice, cabbage and greens or katogo. Some buy silver fish and rice.

I personally like millet bread and smoked fish. Thick pork, chicken stew or greens, although sometimes we don’t have money to buy passion fruits i.e. sometimes the season is ad.

R3: I want to state that am influenced by the season when it comes to the food I eat. When it’s a season of sweet potatoes, I will eat sweet potatoes, when it’s a season of maize, I will eat that and the same applies to greens. If it’s a season of greens, there are those which grow by themselves and I eat lot at that time.

I also stock dry foods for my children always. The children don’t want to eat the same kind of food every day they will always tell you that they are tired,

R4: I want to first say that I am used to eating soft foods like matoke and rice, then at school, we had a cook who was very dirty and would cook very dilute food. We complained and he was discharged from his duties. Unfortunately, we still had to deal with posho and beans everyday till we complained and he was dismissed,

There was a time when we over ate beans and posho every day until a student got sick and now there is some change of diet. The teachers also get to eat fish, chicken, meat and other foods. So these days we are healthy. Songs of ulcers among the children is out. We also take milk once In a while.

We complained and said that a poorly fed children will sometimes perform poorly because of lack of meal and that’s improving now.

M: Ok

R3: at home have adolescents but the girl wants only soft foods whole the boy eats any foods. The girl want rice all the time. She even says that they were told to eat rice at school.

Then at school, when it comes to break time, adolescents buy eats from vendors who sell chapati, mandazi, cassava and rice balls. But cassava isn’t much eaten like the other foods and the vendors go back with it. They like eating chapati every day and don’t eat to heed to advice of cooking well. They like eating fry foods and don’t believe that cooking oil is very dangerous. Some schools have adjusted and are also frying the beans on top of all the fried eats the child is buying leading to a health hazard.

R8: At home we are blessed because I did food and nutrition at school which has helped me plan for my family’s meals well in terms of a balanced diet. However, when it comes to teenagers, they sometimes desire specific foods however much you try to cook a balanced diet. They prefer eating rice all the time and parents nowadays do what their children want. We eat a lot of rice and no complaints. We usually eat beans, g-nuts and soy sauce.

**M: What about at school?**

R8: most times schools provide posho and beans whereby some fry and some don’t fry. However, in our school, beans are fried although some of these teenagers get tired of the same diet everyday that’s when they get beans from the kitchen and buy chapati or cassava instead of eating posho. Some eat posho with avocado and leave the beans other decide to take tea and daddies from morning and dodge posho. There is always a change of diet once in week which makes these children so happy that they come back for 3rd round.

**M: Thanks so much**

**We request to know, what do you take as healthy eating habits in your and your family’s daily life?**

R4: In my family, I always insist on eating vegetables and fruits.

**M; what’s the food value?**

R4: Vegetables and fruits have a lot of vitamins.

R2: I also like buying fruits and avocado and greens which are not fried.

**M: What are the food values?**

R2: They work as a brain booster.

**M: Ok.**

R3: people should eat more of greens and silver fish in order to improve their brains. Most times peop**le** like fish but the fresh fish is the only nutritious one.

R2: every meal should always have greens because it helps build the immunity and chases away diseases. Then silver fish will help in bone growth of these children and their skeletons will be strong.

**M: Ok.**

R8: A healthy eating habit to me is when a child is eating from a clean place ad also takes I a lot of fluids like water which helps in digestion so that the child is not dehydrated and we avoid issues like constipation.

**M: Drinking what?**

R8: drinking water, juice, porridge or tea is healthy.

R1: To me a healthy eating habit is when a child eats while seated ad doesn’t move around with food, it helps to avoid choking. Then they should eat a lot of starch because it makes the brain docile. They should also eat fruits like water melon for the easy breakdown in the body plus taking a lot of water. They can also eat fruits like watermelon or bananas.

**M: Ok. Is there any other submission?**

R7: to me health eating means eating foods that are have little oil in them or not fried at all. for instance if you cook matoke, eat with boiled g-nut sauce or you can just cut boil it with tomatoes, onions and g-nuts. You can eat greens once in a while especially the red greens, you also take milk to build the body. One can also eat eggs and importantly cook from a clean place and use clean utensils to maintain that hygiene.

M: Ok.

R6: As for me a healthy eating habit is eating a balanced diet which is food with all nutrients. For instance, you can have a plate with rice, matoke or yams. When it comes to sauce you come to sauce you can put g-nut sauce, greens and an avocado. Then in a week you can eat a boiled egg because it’s very useful to our bodies. It builds the body since it’s a healthy food to eat. Then you can accompany it with a juice. I prefer juice to soda.

**M: What challenges do you and your family face when it comes to eating a healthy diet?**

R5: The biggest challenge is financial constraints. Sometimes however much you try to economize, you find yourself touching school fees and you end up fail to get a balanced diet.

R1: for my case, I am a farmer who can’t fail to have some food items in my garden like passion fruits, matoke and greens and I mostly do my farming when I am not teaching at school. The challenge I get is that I come back very tired with my cassava that I boil and take with tea. I always fail to find time for fruits or even getting fresh passion fruits to make juice. I always feel it’s a time waster. Then during working days, I always have leftovers of yesterday’s sweet potatoes or rice which I take with hot water ad go to teach. So I fail to get time to prepare proper healthy meals due to the work pressure and being exhausted.

**M: ok.**

R2: We have so many household members and each wants to eat differently. Sometimes you can bring eggplants home and some say we shall not eat that, then the next day you bring silver fish and they refuse to eat it. So you find yourself abiding by their wants and eating what they prefer and failing you to prepare healthy foods.

**M: Ok. Are those adults or children?**

R2: Sometimes its adults sometimes children.

R6: The challenge I encounter is failing to get fresh foods in the market because I love my food fresh. For example you can find when a mango fruit which has taken some good days and you just buy it because you know the food value of that mango.at times they even sell spoilt in fruits to us.

R5: I also want to talk to about finances. We have a lot of expenditures to do yet we want to eat some healthy foods. Sometimes I get into conflict with my sons because they always feel that I don’t consider their desires yet I want them to eat well but due to a lot of expenses, I fail to meet their expectations. Nowadays I eve hide money so that they think not there.

R4: We are many at home and other family members are allergic to some foods. For instance when we want to eat meat, we fail due to at least 5 members being allergic to meat. Yet fish is very expensive. Sometimes we get a special meal of eggs that day.

R7: The biggest challenge we have is finances where by when we get a salary, you stock dry foods ad fail to get other essential foods due to money. Our children ask for different meals for a change but no finances.

R8: to me, the challenge is time. I always see that cooking different dishes is time consuming.

**M: Ok.**

**So what motivators do you and your family have when it comes to eating a healthy** **diet?**

R5: What motivates me is that it depends on the type of food because if I make millet bread, I have to eat it with pork or meat.

Secondly it’s the neighborhood pressure. When my children see our neighbors eating well. They also start demanding for fruits, chips, some chicken ad i have to look for those items so that they dot admire others.

Then I always see food chart at health facilities which are very educative on the right food s we have to eat. That really motivates me.

**M: OK.**

R5: What motivates me to eat a healthy diet is whenever I eat healthy, I don’t fall sick. Secondly is whenever my children see my neighbors cooking greens, they come back and ask me to buy for them too. i buy vegetables so that I don’t disappoint them.

R1: What motivated me to eat healthy is the people I meet who look very healthy. When I discovered that they eat, fruits, avocado, greens, I also started copying because previously I used to think that it was about eating meat and chicken.

Secondly it’s the money. Whenever one gets some money. It pushes you to buy what you have o teen eating every day. You will start buying bread, blue band, eggs.

R4: Whenever I am stressed, I don’t to show people that I am stressed, I go and buy every good thing including healthy foods so that people don’t know what is happening to me.

Secondly us whenever I am happy, I eat healthy foods,

**M: You are right.**

R: What motivates me to eat a balanced diet is generally I am used to it and I find myself balancing the foods even if I am sick. Our mom natured us to eat natural things and she always tell us that if you eat natural foods, you will reduce the chances of going to the hospital. I feel I should have a body appearance that looks good.

**M: Ok.**

R6: personally I have knowledge of the importance of a balanced diet where I know that if I eat, I will be healthy because have eaten the food values that my body needs.

Then sometime heart desires to eat. Then it pushes you to buy what you want to eat.

**M: Now we would like to discuss about the eating habits of young people in this community.**

**What do young people typically prefer to eat on a daily basis?**

R2: Those young people mostly like eating rice, chicken, and meat. However, when it’s sweet potato season, that’s what they eat; when it's maize season, the same applies. Even greens depend on availability.

**M: Which chicken?**

R2: Fried coated chicken with a bottle of soda. There are those who drink alcohol and Rolex. Eat that with boiled foods which they call bad food.

R1: they like eating sweet things like sweets, and soda. They don’t like taking water.

R4: They like eating kikomando, pineapple juice, potato chips, the girls prefer chicken ad chips, fish and chips,

R5: most times young people like eating energy giving foods most especially carbohydrates. The girls like soft foods.

R8: Most times young people say that its poor people who eat boiled foods so they dont want to eat.

**M: Today’s young people also prefer to drink sweet and sugary beverages. What do you think about that?**

R5: Its true because my so likes eating sodas and jolly jus juice.

R6: I have observed that gird like ice-cream because it’s very sweet ad they enjoy it.

R4: they like taking sweet drinks because they say it gives them energy. Things like sodas ad jolly jus.

R2: Students often talk about foods they’ve seen on TV or their favorite celebrities eating. It definitely affects their choices.

**M: ok.**

**Someone mentioned food vendors, so how does the presence of food vendors influence the dietary habits of young people?**

R3: the first fact about food vendors is, their foods are affordable that a child can get at rice balls at 100 UGX or katogo at 300UGX.

R7: The problem I see is that sometimes these guys sell cheap but expired foods because they font throw away left overs.

**M: I want to ask whether you have some model farms at your schools where children do the garden especially in greens.**

R7: it’s seasonal/

R2: they only plant when it’s raining.

R1: we plated vegetables last term but the rains have even scarce.

**M:**  **Let us also discuss about some of the barriers and challenges to eating healthy among young people. You talked about most of them.**

R5: We mentioned all the barriers. But at school, there is a key challenge; most schools provide posho and beans; some fry them, some don’t; but it’s what is consistently available

**M: Yes, you did.**

**What can be done to promote healthy eating among young people at schools?**

R2: We can sensitize them at schools.

**M: How can this intervention it be done?**

R2: through healthy food eating competitions where other children will learn from others how to eat healthy.

We can also sensitize them through adverts like the way mountain dew does.

**M: Who should be involved in designing this intervention?**

R1: The Teachers ad parents.

R: The Students

R5: prefects, media, parents

**M: What challenges do you anticipate and how should they be addressed?**

R5: lack of finances.

R6: Priority of school programs where they have other priorities.

R3: Some children lack interest and they get bored quickly.

R4: Negative attitude of parents because they don’t want to attend school meetings.

**M: So what Solution to these challenges?**

R5: We need VHTs to come to schools and convince head teachers how important nutrition programs are so that they also priotised them.

**M: what about the issue of finances?**

R4: we can do fundraising through house patrons from the learners and the administration to support financially.

R2: If the government ca support these programs by recruiting more teachers for food and nutrition.

**M: Do you have teachers for nutrition?**

R 6: It’s not a common subject but the books are there.

R7: another intervention I propose is that the nutrition subject should be made compulsory.

**M: all schools should have a trained nutrition teacher.**

**M: who should be involved?**

R4: Ministry of education and sports.

R5: NGOs, WHO and MOH.

**M: what would be the challenges to this?**

R5: Sometimes government doesn’t consider some issues unless the NGOs intervene.

R8: There will be corruption.

**M: So what would be the solutions?**

R4: A strict law on financial misuse should be enacted by the government.

R2: If the government lacks finances, they can at least empower the teachers with the skills.

R1: Government should attract in more implementing partners to support.

R5: The biology teachers can be empowered more because they have a topic on balanced diet.

R1: Strict monitoring and evaluation.

R7: We can use music dance and drama and award the best students.

Then the subject should be more practical especially in cooking demos.

**M: Ok.**

R1: We need to sensitize parents to support their children.

**M: Thank you.**

**M: Ok. Thanks for accepting to give us your time.**

**FGD_TEACHERS_PRIMARY SCHOOL_June_2024**

| **Participants number** | **1** | **2** | **3** | **4** | **5** | **6** | **7** | **8** |
| --- | --- | --- | --- | --- | --- | --- | --- | --- |
| **Age** | 43 | 30 | 29 | 25 | 32 | 26 | 36 | 35 |
| **Household size: Participants should have different household sizes (those from small and large household sizes should be prioritized)- insert number of people** | 5 | 4 | 5 | 3 | 4 | 4 | 8 | 6 |
| **Marital status**   1. **Single never married** 2. **Married/staying with partner** 3. **Separated/divorced** 4. **Widowed** | B | B | B | B | B | B | B | B |
| **Disability (A-Yes ; B-No)** | B | B | B | B | A | B | B | B |
| **Subjective SES: A-above average ; B-Average ; C-below average** | B | B | B | B | C | B | B | B |
| **Religion: Participants should have different religions (A-Catholic; B-Anglican; C-Born Again; D-Pentecostal; F-Muslim)** | Participants represented different religions (catholic, protestant, born again, Muslim and others) | | | | | | | |

**Transcript**

M: This is XXXX (CSO) going to conduct a focus group discussion with teachers of primary schools in Mayuge. So, the time is 11:09am, we are going to start. Thank you so much for giving the opportunity to speak, let me use the central place for all the two. So, I have the first Question to ask that, Aaaah… if you talk about food habits and cooking practices in your community, in your schools, in your house holds, what are some of the common cooking practices in your communities, in your schools if I may use it to schools because you are teachers. Maybe before you submit, you can actually interact in any language, you can speak Lusoga, Luganda or English, those ones I will understand, so what they mostly cook in schools.

R6: This is No.6.

**M: Yes.**

R: In most of the schools in go to, the common type of food that they really prepare and cook is posho and beans.

**M: Okay… any other addition, thank you No.6.**

R7: This No.7. Aaaah… some of the schools can mix some vegetables in beans plus posho and sometimes they also maybe on weekends for Boarders the school can prepare some little rice.

**M: Okay… so I have learnt that they cook some vegetables and rice and meat over the weekends. Any additions? No.2…**

R2: No 2. Some schools add some wheat flour in the beans to make the sauce heavy.

**M: So, the wheat makes the sauce heavier?**

R2: Mmmmh…

**M: Okay. thank you. Aaahm anything apart from the vegetables, beans, rice that they normally cook in the different schools. No.3.**

R3: Yeah, No.3. Particularly, I will present the ones that are cooked at school since it is in a rural setting.

**M: Mmmh…**

R3: For us they usually take beans, posho and sometimes some greens but on rare occasions even some meat, that’s on weekends.

**M: Thank you so much. Now I also wish to understand what constitutes health eating habits in your schools? Is the question clear or I repeat what constitutes a healthy eating habit in a given school from your own perspective?**

R5: I don’t know if I have understood, thank you very much, I am No.5. Aaaah… are you trying to talk about the ranging content or the chemical content of the food like the carbohydrates?

**M: I just want to understand what you as teachers take to be a healthy food.**

R6: Now, healthy food is one which is composed of a number of classes of foods like the carbohydrates, proteins, fats, vitamins, water and the mineral salts which I believe in one way or the other, they are normal provided but in smaller amounts.

**M: Thank you. I want to reduce it to the local context of a pupil feeding in this school or another school, what do they eat and you feel that they have a healthy diet.**

R6: Aaah, I am No.6 presenting.

**M: Yes please.**

R6: In my opinion, when it comes to feeding learners and the young ones for them it’s a bit different.

**M: Mmmh**

R7: Again, what entices them, is something that may contain less contents of diet, but for them, they are all about something that is rare, that they need once in a while. Actually, if we are to sample, if we happen to give them a meal of rice, for them they really feel so happy and contented and making it worse if we combine it with a sauce which is meat again the excitement goes beyond the normal.

**M: Do you think they take that to be a healthier diet or is it because they take long to eat them.**

R4: To me, I think it is not healthier like the way you said but they take long. But in my opinion, I believe the one which is healthier is posho and beans.

**M: Okay... why do you think posho and beans are healthier compared to others?**

R1: I believe these two contain the biggest percentage of proteins.

**M: Okay thank you. And I want to still understand typically when you look at their plate as adolescents, do you think what they eat school differs from what they eat at home or it is relatively similar?**

R3: Thank you, actually what they are fed on at school in terms of quantity is far smaller than what they eat at home. However, there are categories of learners who manoeuvre their ways and they are given our food by the cooks at school so that they eat.

**M: So, what does that mean, should I say what has been served is insufficient in terms of quantity or?**

R5: No 5 presenting. Basically, as you introduced the topic you talked about adolescents, and adolescents have a number of characteristics. It is actually a period of storm or stress, there is some development in them, they feel they need more energy, so you notice that even when it comes to fluidity, they require much fluids in order to mobilise their energies.

**M: What are some of the challenges that you face at school while providing a healthy diet to these adolescents.**

R1: This No.1 presenting. At school, the challenge we usually face when providing these adolescents with a healthy food, some other times it is a financial crisis, where you find that in these rural settings, some of the parents, lack the motivation to provide the money so that the school can also use to see that we can also provide for them food.

**M: Okay so, the first challenge I have learnt about is the financial constraint, and I have been informed that this makes it difficult to buy the food you want. Aaah… any other challenge?**

R7: This is No.7, the school may go to buy maybe meat so that these learners can change on the diet but they always face a challenge that some learners may not be likers of meat, some of them maybe their likes are on fish, so that one becomes a challenge to us (not clear) so when we buy meat knowing that the other group of learners may still survive on beans and not change the diet.

**M: So what motivates the adolescents in your schools when it comes to eating healthy. What do you think motivates them to eat what they eat?**

R4: this is No.4 presenting, normally students like quality food.

**M: Mmmh…**

R4: Food which is good to them, more so, fatty food. So when you put a lot of cooking oil, they prefer it.

**M: So when the food is fried, it motivates them to eat?**

R4: Yes

**M: That is what I have understood. Okay any other motivator?**

R7: No 7. I think the quantity normally served, will also motivate them, because normally there are those who transfer them from other schools to others, because in those schools they serve more food than their school.

**M: So when the quantity is sufficient, they are surely taking it?**

R8: They will always want to eat.

**M: Does this differ between boys and girls?**

R8: Another motivator will be the activities at school, the kind of activities they involve in, will motivate them to eat, there were these games and many other games.

**M: What do the young people prefer to eat on a daily basis, is there anything that you have observed that they prefer to eat on a daily basis? We are talking about preference not what is available.**

R7: Actually, to me, I think it bases on the locality and also the cultures.

M: Mmmh…

R7: Like I have moved to very many areas, like in Busoga here especially the typical Basoga, potatoes is the staple food, when you go to districts like Namayingo, Busia, where we have very many Samyas its kalo, then when you go up like in Northern Uganda where we have the Acholis and more and the Langis still it’s the Kalo, so I believe these Learners may take food as their preference basing on the locality and also the tribe.

R5: Whenever we cook unfried green vegetables, they [AYAs] refuse to eat food because they want it fried. They say that unfried food is not tasty.

R4: They [AYAs] prefer eating good and sweet things. Like at my school, they say that if you eat sweet things, you also become sexually appealing… that is their reason, and that is how they understand it. During that adolescent stage, when they eat sweet things, they believe they will also become sexually appealing to their partners

**M: Okay, and you have highlighted that the Basogas find it easy to eat potatoes, the Samyas, Kalo, also the Langis…**

R7: Yes

**M: Okay, any other thing that influences their preferences other than culture and tribe, that you have observed? As we think through, we have also noted that today’s young people prefer to drink sweet and sugary beverages, it that correct?**

R2: That’s correct.

**M: So we want to understand, what do you think about that, your general thinking about their preference of sugary beverages?**

R5: Its No.6 presenting, in my thinking its due to the advancement of new technology and also the change in the generation where we are living because in our time, we could not find any harm in taking tasteless hot water at times when there are no tea leaves but here because of the advancement in people’s earnings, decentralisation of resources, that parents can get easy access to sugar and other sugary contents it makes the young ones to grow while adapting to such things because they are in the reach of parents who in most of the times provide them so they grow and they are addicted.

**M: So, you think they are eating because of the current technology?** Okay, any other thinking?

R7: Thank you very much, No.7, Aaah… just to supplement, when I look at most localities especially the localities we are living in, there were indigenous foods which used to be there, but they are no longer being embraced in some of these communities especially in some of the families because of the changes in the financial status changing. Aaaah… you notice that some people do not embrace having vegetables, greens, but via these sweet things, they like them. You find the refrigerator is having, yoghurt is there, juice is there, sodas are there, and basically there has been lack of information to we the adults and the young ones, we have not given them information about what kind of diet they need, what kind of diet that will not become a problem in the future. So they also keep on taking the sweet things and leave the other things.

**M: So, do we have food vendors in our schools, this could be a canteen, a basket vendor from the fence of the school, do we have that?**

R3: Yes, we have them.

**M: So, how does the presence of those food vendors influence the dietary or eating habits of these young people, what would be your comment about their influence on the eating habits of the adolescents in your schools?**

R8: Its No.8 presenting, sometimes you find that these young adolescents, when you compare them with is presented by the vendors, you know there is what we call monopolising things, for them they will end up buying what is presented to them because they may not have the option of going anywhere. So, like if its break time, maybe someone wants the juicy things that we have talked about but there is nowhere to find them, then that child will end up buying what has been presented, I always see that.

**M: So, what is always presented?**

R1: Always Juice and some bread I think… dough nuts, mandazis.

R3: Then a simple addition, these vendors have also adapted, the modernisation as my brother was saying, they don’t bring these ancient foods, they bring the modern food that suits the prevailing conditions where these learners are developing in, because now we are no longer seeing yams, we are no longer seeing these other traditional foods that for us we used to buy those days

**M: They don’t bring Katogo…**

R1: Aaaah… they rarely.

**M: Okay.**

R3: in my opinion, I believe its learners who determine what these vendors must show, its all about the learner’s preferences, so these vendors are influenced by what learners like most because of their age.

M: Mmmmh…

R2: Because, let us say you are a food vendor, you do not stock what will not be shopped very fast because you are trying to balance the diet, you need to look at the consumer preference, what do your customers like most, when these vendors are shopping, they target, first of all they look at the age of these learners, and basing on the age, what do they prefer and in their stages. So you realise that these children for them are more interested in such things which contain these sugar contents and they are of low prices.

**M: So they dictate what the vendors should bring?**

R2: Yes

**M: Okay, interesting, So in that regard I want to know whether there is any role that the school have played in influencing the dietary habits of these young ones, have schools played any role?**

R7: Aaah… the schools actually play a great role in influencing the dietary habits of learners in such a way that, not that every learner, even if you have talked of these vendors bringing food in our schools, some learners may not be having money to go and buy. These schools sometimes do provide some edibles to the learners, especially breakfast, they can say, maybe if there is somebody making chapatis, they can make an order and give to the learners, which helps these learners to change the diet.

R7: In addition to that when you look at the way how some other schools have come up with a policy of adding up to change the diet of the learners, they have also looked through and also come up with a menu, and at least once in a while you see that their learners change. They can say that every Wednesday, let them have rice and meat, just once in a while to see that they can cope up and balance the diet.

**M: Okay, do you want to add anything?**

R7: No…

**M: Okay, we have discussed about some of the barriers and challenges of eating healthy among the young people, but could there be… or my question would come again saying, what are the significant barriers to the young people eating healthy foods, from your experience, what is that thing considered to be the most significant barrier?**

R3: Yes, thank you, No.3 presenting, for me I think, the most significant barrier is finance. When ever the school lacks money, I think it will not afford to provide a balanced diet to the students.

**M: Yes…**

R4: This is No.4, in addition to what my colleague has said, the high cost of these foods, so schools find it difficult to buy those different types of food, because they are more expensive and costly.

**M: Mmmh….**

R4: Being agricultural products, they keep on fluctuating, which is hindering giving them a balanced food diet.

**M: So, Aaaah… do you think the foods are available but they are just not affordable or they are not just not available. What is your comment on that? No.8 does you want to comment on that?**

R8: The foods are very available but like my brother has been saying that some foods that are available are very costly.

**M: Mmmmh…**

R8: And therefore schools have minimal, amounts that they cannot be able to divide and buy to give to the learners, but me I have also another concern, some of the schools have remained conservative, because now when we talk about this diet, so of these foods are very cheap, take an example of schools which ae giving beans throughout, we have the egg plants in the market, we have these greens in the market, those things are very cheap, but now some schools or some leaders are just conservative to changes. They just look at that as, they don’t take it as serious, me it’s what I see sometimes.

**M: So I want to understand, do you think tastes and craving of these adolescents could be the reason why these people are conservative?**

R8: Sorry?

**M: Do you think maybe it is because these foods are having a different taste, and people don’t crave for them?**

R7: Not as such…

**M: Okay, you wanted to add something…**

R6: Actually am jut supplementing, this is No.6 presenting. In the very line, to me I think they are these 3 factors influencing, 1 is accessibility, at times accessibility goes with locality.there are certain food stuffs which maybe available, in place but when they are inaccessible. Two…

**M: I want to understand that clearly before we go to 2, inaccessibility.**

R2: Like we have very many food stuffs on the market, but you find that when you look at the location of the school, the school may not in position to access that market, and therefore to access such kind of food stuffs.

**M: Is the accessibility related to no road, no money, high cost of transport, distance?**

R2: Actually, it is related to expenses and also the distance in between.

**M: Okay, now number two.**

R2: Number two, is lack of awareness or lack of information about the importance of such food stuffs.

**M: Mmmmh…**

R3: Because even the locally available food stuffs may not be consumed to the maximum because people lack information about their importance in the body so at times, some of the food stuffs are not consumed because people are not aware of their importance.

**M: So that takes me to the next question, what can be done to promote healthy eat among the young people? Because we have seen the challenges, we have seen a number of them so what do you think going forward could be done?**

R5: Thank you very much, No.5 presenting, aaaah… maybe I want to make soe approach n this form which I believe is now going to help us all, that is the social, the political, the economic… I am forgetting the fourth here but I think there are four broad that I have been thinking about, whereby each one has a way that influences. Forexample if I may have you come again then I give you the social bit of it.

**M: I want to understand, what can be done to promote healthy eating among these adolescents.**

R5: What can promote…

**M: Yeah, how did you come up with the concept of social, political, economic and the forth is about to come.**

R5: In our schools especially these rural schools, we have concepts which are political, that schools should not charge any money. That means the learners, even the posho which can be bought from the near by centre, and the beans, the school leaders will say, haa… we can’t charge. The learners will there, the administrators will e there, they will move on hungry, there and there and again and again. Then the social part of it, once, actually the political will now influence the social where the parents, the guardians and the rest of it, having heard that there is not charging, RDC said we are not charging any money to the school, they will also remain reluctant… I will not pay money for feeding. And given the situation the learners come home when there is very little food, now, getting little food at home, and no food from school at all will again make an influence on these adolescents, they will develop other mentalities, other behaviours will come in to supplement on what they eat at home. Either some will go to look for food from peoples’ gardens, to look for jack fruit, to look for sugarcane, others will not even reach school, they will want to go and look for money to have some money, but if the vendors come, they buy for themselves.

**M: So, going forward, how should we address this challenge?**

R8: Okay, thank you, No.8 presenting, Me I feel that if we are to address all those challenges, the first issue I would consider is sensitizing. If we are sensitizing, the importance beginning from, sensitization will be done on various levels, it can be done on family basis, it can be done on society, the local areas, it can be done in schools by various stakeholders lets say if it’s a school, we always have people who are concerned in the feeding department so if those people come up and transfer the information and give to these children. So if we can sensitize, the school leaders can inform the children the importance of health diets and even as we are talking to these children we can hold a parent meeting and teach these parents the value of the children to be given such foods that will help the school administration because as these parents are being informed they will be able to support the children. Sometimes there are things where we will need to challenge the government decisions because the government will say, you should not send away children from school because of food by now if we call these parents and we give them the value as to why these children should have food at school, they may end up supporting these children.

**M: Because that was my next question that what are the challenges do you anticipate if you choose to go that way, yes do you want to comment?**

R6: Mmmh… for me I wanted to just add on what my colleague has said, we have some directors and principles of the schools. Some are businessmen and they are not aware of what a balanced diet is, and its purpose therefore we need to engage them and maybe via sensitization meetings like some things are available in the communities like those greens and the like but they are not aware what a balanced diet is, sometimes they think that when they provide rice and meat that is over.

**M: Okay, so we wanted to anticipate the challenges that are likely to come in the due course of sensitization and advocating for healthy eating.**

R5: Thank you very much, this is No.5 presenting, I feel the teachers in schools can be good help, especially the rural schools even the town schools can have what we call gardening school, those are kind of gardens which are not far away from the school but are within the school compound, farming in bags, farming in jerricans, if they can have like greens planted within the bags around the school, around the verandas, which they can harvest once in a while, and make a nice meal, which impacts on the children’s minds that if am at home I can do something. And then those that have land, the rural schools, I think those ones, the agriculture, biology even all other teachers can come on board and then they start doing some farming. Which can improve on the diet.

**M: So, I asked about the possible challenges, he highlighted that at some point they may have to challenge the government programs but other than those challenges of policy where the government is saying this and that, which other challenges do you anticipate to face when advocating for a healthy diet?**

R5: As you advocate for that, you realise that culture differs. There are some communities where as you are talking about a good diet, fish must be part, without fish…

**M: its not a good diet…**

R4: Its noting, in such areas, you need to address them like he talked about sensitization. We need to have teams, either voluntary teams, or incorporated into policy, and they move to talk about diet. You know am told in the Health Centers IV, am told there are those nutritionists I think, in hospitals, but their work I think is office work, they don’t go down to meet communities if they go down, it means they are signing something, to reach there and say “you people…you know” and they come out.

**M: At all Health Center IVs, District Hospitals we have either a nutritionist or a nutrition focal person, so, your recommendation is that they should come to the grass roots…**

R4: Come to the grass roots where the adolescents are…

**M: And they teach them about a healthy eating diet.**

R4: Yes.

Thank you for participating

**KII-basket food vendor_secondary school_June_2024**

| **Participants number** | 04 |
| --- | --- |
| **Age** | 26 |

Interviewer (I)

**I: First tell me about yourself. Are you married?**

R: I am married.

**I: How old are you?**

R: Iam 26 years old.

**I: How long have you been vending food stuffs?**

R: I’ve been doing it for 5 years.

**I: What your level of education?**

R: I stopped in Senior 4.

**I: What religion are you?**

R: Iam a Moslem.

**I: What’s your tribe?**

R: Iam a Mugishu.

**I: Ok.**

**Can you tell me what healthy eating means to you?**

R: To me helps a person’s body to be healthy because they eat well. . Secondly eating healthy also means that one will be able to chase sicknesses especially these that are bacterial and the airborne diseases because the body will be having strong blood to fight against diseases.

**I: So the blood will be strong.**

R: The blood becomes strong when a child eats proteins, carbohydrates.

**I: Ok.**

**We shall expound on that later. Is there any other thing that describes what eating healthy means?**

R: One will eat chicken and says he eats healthy then another one will think he didn’t eat well because he ate greens yet he ate healthy.

**I: Ok.**

**So what foods do you consider as healthy foods items?**

R: I consider carbohydrates to be healthy food items especially cassava. When a child eats cassava, it helps because its energy giving. Then we have energy giving foods like posho, sweet potatoes whether salted or not. children enjoy eating them. then are foods that can’t be eaten with salt.

**I: What other foods?**

R: Other foods are rice, matoke especially the older adolescets.childre like eating Irish potatoes and its worse when they smell it from the neighbors. Sometimes its poverty that limits us but it would be good if we cooked for them different types of foods because they have different functions in the body. \

**I: What other classes of foods do you consider healthy apart from carbohydrates?**

**I: Ahhhhh**

**I: What about sauce?**

R: Most times one needs to eat vegetables, beans, gnuts, meat, chicken, and fish. It’s good to mix.. If i eat meat today then vegetables tomorrow, there I will be reducing on the bacteria’s that i might have got from the meat.

**I: How?**

R: Most times vegetables are medicinal. or i eat my pumpkin leaves.

**I: Ok**

**So what foods do you consider unhealthy?**

R: To me if one is eating the same type of food apart from posho, it becomes unhealthy because there are people who like eating rice daily. yet it of no use in the body.

**I: like what food?**

R: like rice. I consider rice to be unhealthy.

**I: You also mentioned posho.**

R: To me posho is a good food but not rice.

**I: Do you consider rice unhealthy?**

R: Yes I consider rice unhealthy.

**I: What else is unhealthy whether at home or bought from vendors?**

R: I really can’t tell.

**I: Ok.**

**What about junk food?**

R: Those are foods which contain a lot of oil and makes one very thirsty.

**I: like which foods?**

R: like fried Irish potatoes. Whenever you eat junk foods, you become very thirsty.

**I: Irish potatoes or chips?**

R: The problem is that people here fry rice and Irish potatoes with a lot of cooking oil and it causes a lot of thirst becomes unhealthy because drinking water excessively causes weight gain.

**I: What else?**

R: Chips and chicken. Fatty fish especially the one sold per kilogram.

**I: The one sold in kilos is so fatty?**

R: It’s really fatty and mature.

**I: So what is your opinion about the eating habits of young people in this community?**

R: The young people in my community like eating snacks, chips and chicken, cassava chips, mandazi, chapati,

**I: How healthy or unhealthy are their eating habits?**

R: Too much oil is not good for their bodies but just because we are ignorant and sometimes we say that we eat what our hearts desire. When you tell young people to reduce on the consumption of oily foods they think you are stopping them from eating well. Those foods are unhealthy but explaining to people is also a long process.

**I: What are the major health concerns associated with young people eating unhealthy food?**

R: There are people who get dysentery due to the type of food they eat. Others get diarrhea, malnutrition.

Then some people eat for instance lunch at 1pm they eat the next meal the next day at 1pm this contributes to getting stomach ulcers.

**I: What other health related issues?**

R: Some people develop dysentery due to the foods they eat, others diarrhea or **malnutrition** due to eating poorly. They cannot look healthy because they don’t take juice or eat properly in time. If a person eats at 6pm then again 6pmthe next day there will be a difference. That’s the major cause of ulcers.

**I: What other health challenges?**

R: Kwashiorkor also affects people who have poor eating habits. Sometimes due to poverty, one will not even have sugar for tea and will be used to eating left overs as well that are full of insects which can as well cause stomach issues like diarrhea or swelling of the belly. This is caused by eating cold foods without any hot water.

**I: What about the older youths 17 to19 years, what diseases do you think can affect them due to eating unhealthy foods?**

R: I see them looking so bad, some will have reddish hair, others lose weight just because of poor eating and sometimes ulcers.

**I: How do young people make food choices? Let’s start with boys.**

Most youths want to eat good things to the extent that they dodge classes the next day and go for odd jobs in the sugarcane plantations so that they get money for buying what they desire to eat chips which his peers were eating yesterday while he had posho ad greens. That’s the reason they go to make money in the plantations. Though. Most of them prefer eating kikomando (chapati and beans.). This is because you can eat if you have 500 or 100shs.

**I: What about drinks?**

R: They prefer soda.

**I: What about girls that age?**

R: There are girls between 10 to 19 years who want to slay and don’t want to eat things which will cause gain weight, or foods that will increase the belly size no. In case they eat chips plain of maybe 2000shs, they will only take water the whole day without adding any other thing until evening. They think it’s the way to avoid weight gain yet they are adding weight.

R: There is also this traditional issue. From childhood, girls in this community are taught not to eat emamba (lungfish); it is considered taboo for me or any other girl or woman. As a result, girls avoid it; even if they might want to try it

**I: Which chips?**

R: Irish potato chips.

If the girl doesn’t have money, she will take cassava for 500shs and water the whole day. But also, most times those who want to eat well get temptations where by men capture their hearts by buying for them sodas and chapati that’s why we have so many early pregnancies.

**I: Ok.**

**Remind me what you sell?**

R: I sell cassava chips and mandazi. I used to sell samosas but they were not yielding much. Students really like cassava chips.

**I: What are the most preferred food items by young people when they visit your food basket?**

R: They prefer eating cassava chips though they some new innovations on the food. I fry cassava chips sprinkled with onions and they really buy it. Whenever I miss coming, they look for me and ask me why I dint come.

**I: So you sprinkle onions after frying.**

R: Yes I chop and sprinkle fresh onions and sprinkle after frying the cassava.

**I: So that’s what they prefer most.**

R: They really buy that a lot.

**I: Today’s young people also prefer to drink sweet and sugary beverages like juices and soda. What is your observation about it?**

R: They like taking such colored ice sold by vendors. That ice mixed with sugars and powdered jolly jus. They really like eating it.

**I: Ok.**

**So how do they get information on healthy eating?**

R: That information is mostly in health facilities. Those who educate about good nutrition habits take long to come but when you visit the health centers, you get a lot of information how you should eat , how you shouldn’t use a lot of cooking oil, how we shouldn’t use burnt onions etc..

**I: Ok.**

**Who are their role models that they look up to as they learn about healthy eating** **and nutrition?**

R: This generation of kids watches TV and social media. You will find a poor family but with a smart phone. Though they, mostly see adverts of maybe curry powder on TV some adverts from other food companies. When these adolescent watch them they also ask their parents to buy what they saw in the advert for instance they will want to eat chips and chicken or tea with bread, rice with fish or ‘we want to eat the food that we saw Chef Godwin advertising, sometimes the parents cannot afford what these children want.

**I: What ways do community norms and cultural beliefs influence young people's food habits?**

It really affects for instance as a woman I can be told not to eat chicken yet its cooked at home every day and you really desire to eat yet it. Unfortunately you are told that it’s a taboo for you to eat that chicken. It’s a serious issue that has made some children learn how to steal food because they can’t access it due to culture.

**I: Ok**

**FOOD VENDOR’S ACCESSIBILITY, DIVERSITY, AFFORDABILITY ASSESSMENT**

**You said you sell cassava chips.**

R: Including mandazi, samosas, I also sell Irish potato chips in the evening although now I have a baby.

**I: How old?**

R: 1 year old.

**I: So you sell cassava chips, samosas,**

R: Half cakes and mandazi

**I: Ok.**

**How much is fried cassava?**

R: A piece is 100shs

**I: In which currency?**

R: In UGX.

**I: What about samosa?**

R: They are at 200shs

**I: How much are the mandazi?**

R: I sell at 200 UGX because that’s what most of them can afford.

**I: You said that you have been making Irish potato chips?**

R: Yes.

**I: How much is it?**

R: A plate is 3000 UGX.

**I: Do you sell it at school?**

R: Not at school.

**I: let’s look at the prices down in the table.**

**I: Is there anything you want to say before we end this interview?**

R: I want to say that the problem we have nowadays is that the parents of young people don’t listen otherwise they should stop buying for children jolly jus ad eats which are too sugary. These children eat a lot of sugary things and very cold in form office.

**I: Thank you very much for participating in this interview.**

**KII_BASKET VENDOR_PRIMARY SCHOOL_June_2024**

**Interviewer(I)**

**Transcript**

**I: Thank you so much for giving me this opportunity to speak to you I want also to know a few things from you before we start… first of all your age…**

R: I am 35 years old.

**I: Your current job…**

R: Teacher (in-charge of welfare)

**I: Your marital status…**

R: I am married.

**I: You stay in the rural or the urban areas!**

R: In the Rural.

**I: Religion…**

R: I am a catholic.

**I: And your tribe…**

R: I am a Sabin.

**I: Could you tell me your role as a welfare teacher at your school?**

R: One; I have to ensure that there is food for the learners and with feeding we have two categories there are those who only take porridge and there are those who take solid food and this depends on what a parent can afford if a parent wants solid food there is a quantity that is required of a parent like we estimate in a term the parent who wants to have solid food brings 15kg of maize flour and 5kg of beans and in our estimation we estimate that a child takes at least a quarter a kg per day that is porridge and lunch and then those who cannot afford solid food they also bring 5kg of maize flour in estimation that it can push them for the 3 months.

**I: Could you tell me in your opinion what healthy eating means to you?**

R: Healthy eating could be referred to having a meal that adds value to one’s body.

**I: What are reasons you think eating healthy is important?**

R: Majorly one is for proper growth both mentally and physically.

**I: Then two…**

R: Two; at least to prevent certain diseases because there are diseases that originate from the kind of feeding that you use.

**I: Okay, so what do you consider to be healthy food items?**

R: Healthy food items should be food stuffs that contain food values like we prefer majorly posho and beans much as it may look local but we are sure there is some component of carbohydrates and proteins in the beans then fats if we have included some oil to some extent we assume that we are at least providing a balanced meal which has at least more than one food value in it.

**I: Which foods do you consider unhealthy?**

R: For now, of course our preference is posho and beans, the unhealthy foods we look at foods that may interfere with the body system if taken for example all foods could be healthy but depending on how they prepare them like if you prepare posho if it is not ready eventually it will result into another complication so foods that are unhealthy could be those that are not well prepared.

**I: Have you heard of junk foods?**

R: Not really.

**I: Because I wanted to ask what foods do you consider to be junk foods?**

R: It may look to be a new term but to me I think they are referring to foods that we eat but in actual sense they do not help our body.

**I: So what is your opinion about the eating habits of young people in your school?**

R: Well we only provide for sustainability… we don’t consider all the factors that are required of a healthy meal. We look at sustaining the child to push on the day while at school.

**I: So would you say their eating is healthy or not healthy?**

R: It could not be healthy but for sustenance…

**I: Okay, so what are the major health concerns associated with eating unhealthy food?**

R: It may result into diarrheal diseases and digestive disorders so such a child with the two complications may not concentrate in the learning setting.

**I: How do young people in your school feel about healthy eating?**

R: I know they feel they should have the best but the affordability limits them to go by what is available.

**I: But what do you think for them they consider to be a healthy diet?**

R: In general, for them they look at the nature of the food like if you had… take an example if you provided posho and beans then provided rice and meat eventually the child will run for rice and meat so to them they consider these other modern meals to be healthy meals compared to the posho and beans.

**I: So how do young people make food choices… what do they base on to choose what they eat?**

R: They base on what you have at hand… if you have what can enable you to buy the simple meal like we have the posho and beans here, you go for that much as they would prefer to have something better than that but due to such factors… pocket factor now… what do you have you cannot have something which is expensive yet what you have cannot purchase it.

**I: Do you think adolescent boys and girls have the same food choices or it differs?**

R: It differs… adolescents normally like our setting adolescents tend to want to consume bulk food compared to other age categories like you find both boys and girls between the age of 14 to 17 their eating rate is high so you find that we provide but it might not meet their required levels because those ages that is when eating becomes almost part of them adolescents majorly most of them.

**I: So what are the most preferred food items that young people from your school eat and where do they get them?**

R: The ordinary provided is posho and beans then like we have vendors also who come in with boiled cassava, fried cassava much as in actual sense it is the same food carbohydrates that is what children take as a medium of change from beans to cassava those who can afford because this boiled cassava can go for as low as 300/= a child can get so normally those who prefer to change go for vendors that is when they acquire…

**I: So you as a welfare do you dictate what the vendors should bring and what they should not bring in?**

R: Yeah, like we do not dictate actually what they should bring but we take them through the precaution at least for someone to supply food in a school setting they should at least come with a medical form at least recommended by a medical doctor not from any other health center that one helps us because you can’t tell the intentions of a vendor but we look at that so after acquiring a medical form we are sure we are dealing with someone who will not cause havoc to the children then the food that they bring also we also try to inspect to confirm that the food has not gone bad or contaminated there are those who may bring food of the previous day so we also try to check and when discovered children are also sensitized that if you buy something and it is not good at least report and we interface in most cases we stop them those who bring in food that is not standard then the business continues.

**I: Today’s young people prefer to drink sweet and sugary beverages and soda, what is your observation in your school?**

R: It doesn’t differ because we have among the vendors there are those who bring packed drinks in polyethene bags could be jolly jus or any other liquid with food color provided there is sugar they normally prefer running for such because they mostly associate it with soda so they love it really.

**I: So the most preferred drink is jolly jus!**

R: It is the most available though not preferred because it goes for 100/=.

**I: So what else do they take?**

R: There are those who are also… depending on the home they come from who carry their own sugar to supplement in the porridge… those who can afford but our porridge is provided without sugar.

**I: Do these learners or adolescents ever learn about nutrition and eating a healthy diet?**

R: We do it at class level like in one of the classes it is a topic on its own then in the other classes it is just general encouraging that when you eat well you will be healthy and we also try to highlight on which food they should eat so that they don’t think that eating well is eating meat and rice but eating well can mean taking any food stuff that will add value to the body and improve on your health.

**I: What role models do they look up to in order to learn how to eat or those that influence their eating behaviors?**

R: I think they copy directly from we the teachers because as they take posho and beans we also do as well take posho and beans only that we leave them once in a while when we are lucky and our bosses provide something different we also take it as a chance but they are encouraged and there is no way yet they can resist yet they have seen even the teachers consuming the same then we try and we make sure that what we eat is what they eat though slightly for the teachers when it comes from frying the sauce it is slightly different from theirs because for them they are big in numbers so you find that the quantity of spices used are minimal unlike for the teachers.

**I: Lastly, in what ways do community norms and cultural beliefs influence young people’s food habits?**

R: Like some communities eat differently and now a child reaches at school and is made to eat uniformly with the rest like we may have a child from a community where they have Matooke but then here we don’t have Matooke… you find a child comes from an area where the staple food is cassava but then here we have posho and beans so you find that to some extent when these changes come in if they are not used at first it becomes a challenge so it takes time to get used to this food that differs from what they eat at home so that us where we find it as a challenge and a factor influencing the nature of feeding at school and the communities where they are coming from.

**I: So do you sell fruits at school?**

R: Fruits are seasonal if it is the season they bring…

**I: Currently are they bringing?**

R: Currently the season does not allow.

**I: And for snacks you said they sell pancakes…**

R: Bans, then roasted cassava and boiled…

**I: How much are the pancakes?**

R: It is 100/= each then even the cassava it is split at that same cost then for the bans it is 200/=, 300/= they can even go up to 500/= each.

**I: Anything else that they sell?**

R: Sugarcanes, a piece starts from 300/= up to 500/= anything that goes beyond that looking at a rural setting it means children may not afford but there are those who are not intelligent enough who combine they join two to get one piece.

**I: There are no dairy products there!**

R: No.

**I: Okay thank you so much we have come to the end of this interview.**

**KEY INFORMANT INTERVIEW – Mayuge District Health Staff**

**Key**

**I:** Interviewer – TB

**R:** Respondent

**Q:** Question

**P:** Probe

**Q: What are the major nutritional concerns that you see among the youth and adolescents here in Mayuge District?**

**R**: The major concern is that these adolescents do not produce a lot of food by themselves but they eat a lot of food. The adolescent boys eat too much, almost everything that they come cross. The girls do not eat too much but they eat frequently. As a result, some of them have become overweight. For the case of good food supply, I believe we have enough food for these adolescents to eat from as a District.

**Q: Are the nutritional needs of these adolescents met from the current food supplied?**

**R**: Partly, yes. Most of the local food produced here in Mayuge is eaten locally by the family members in most homesteads. The diet is not yet specifically met very well. Most homesteads do not eat or take a long time to eat specific types of food that constitute a balanced diet. For example, fruits, these are seasonal and most households that produce them sell the, commercially this leads to a diet gap in such a homestead.

**Q: What do you consider as the determinants that shape up the diet choices, of adolescent’s choices in Mayuge district?**

**R**: The availability of certain kinds of food determines what to eat. The availability of food in a given season largely determines what adolescents eat. Their diet changes with the season. Peer pressure also influences certain people to eat certain types and quantities of food. For example, there is myth among girls here in Mayuge district that if they eat a lot and grow fat, they will be loved by boys because of their enhanced beauty. Additionally, other factors like sickness can also influence the food one eats. Lastly, some parents, especially those that are well off are able to buy specific types of foods especially those that are not produced locally in Mayuge. This also influences the food types that the adolescents will be exposed to for consumption.

**P: Is culture something that plays a role in these food choices?**

**R**: Yes, it used to be but lately those cultural barriers are not there. Additionally, the Busoga in Mayuge do not have a specific type of food that they are bound to eat as a cultural obligation.

**Q: On what information do you believe, adolescents in this area are basing their food choices?**

**R:** The adolescents here in Mayuge usually eat the food we are ready and able to offer here in Mayuge. Much as they may have their own food choices, they mostly eat what is grown locally. They rarely have the option to choose from a wide variety of foods.

**P: Do you see any problem with the youth just choosing from only the food crops that are available?**

**R**: Yes, this is because it limits them from having a balanced diet.

**Q: What do you perceive to be the biggest barrier to healthy diets among adolescents in Mayuge district?**

**R:** Mainly poverty. Most adolescents are from poor households that can not afford to offer their families balanced diets. This is a big limitation. Secondly, the district produces a lot of food which discourages the people from buying any other additional food that would seemingly have been nutritious. For example, some time back we had a case of an 18-year-old adolescent being malnourished. This shows the nutrition gaps in our current food supply here in Mayuge district. Information and knowledge about effective nutrition for households is still a very big problem in Mayuge district. In most cases the information is there but the people do not know how to balanced the available food stuffs effectively.

**Q: Can you propose ways to effectively overcome these challenges gaps you have stated mainly the information gaps?**

**R**: The most effective way is to sensitize the people in communities about balancing the diet quite often. Secondly, is through trainings of these adolescents about nutrition using the VHTs, teachers in schools, peers and health workers at health facilities.

**Q: What role do you think the availability of certain food options in the adolescents’ environments play aiding them have a healthy diet?**

**R**: Positively, the availability of various food options aids the adolescents to have access to a balanced diet. Negatively, the adolescents rarely have access to various food options so, they have no room to make choices. They eat the food stuffs that are readily available and produced here in Mayuge which to some extents are limited.

**Q: What is your opinion about adolescents making choices about specific food stuffs like junk food?**

**R**: The food choices of some adolescents to eat fast food stuffs like chapatis, chips etc influences the food choices of their peers as well. This leads to adolescents opting for food choices that are not as nutritious which can potentially lead to malnutrition. Selectivity of food choices by adolescents is mostly common among the girls who prefer junk food like chips and fried chicken. This has even forced them to start looking for men to sponsor their lifestyle which has resulted to consequences like early pregnancies.

**Q: What role do you think the lack of specific food choices in the environment of the adolescents play in discouraging a healthy diet?**

**R**: Lack of specific food choices in the environment of the adolescents can easily lead to malnutrition due to lack of specific food nutrients in them. Additionally, in a bid to get access to a nutritious diet, these adolescents and the families they come from end up incurring high costs to purchase these food stuffs which greatly impacts their incomes.

**Q: What kind of programs are implemented at the national and district level to promote healthy eating habits and improve adolescents’ nutrition?**

**R**: For specifically adolescents, we do not have a specific program for them but a had a program for children under 5 years. We have none at the district level here in Mayuge.

**P: What was the main objective of the program for the children under 5 years?**

**R**: During our nutrition assessments, we realised that children who were malnourished were not particularly from poor families but were as a result of their care takers lacking the right nutrition knowledge. We therefore started this program called PD Half. Under this program, we had role models, the mothers who had nourished healthy children and we trained them. We sent them to their respective communities to teach mothers who had malnourished children on how to raise their children in a healthy manner. So, the cardinal objective was to teach better nutrition practices to mothers who had malnourished children using mothers that had raised healthy children as role models using the locally available food stuffs. This worked greatly.

**P: What other activity did you do during the implementation of this program?**

**R**: The other activity was that we would feed these malnourished children for the first 15 days of their inception in the program. This food was locally collected, provided and prepared by the peer mothers in the program.

**Q: What was the role of the district in the implementation of this program?**

**R**: The district mainly provided the nutrition knowledge through trainings.

**Q: How frequently did you carry on the trainings?**

**R:** The training with the peer mother or the role model mothers took 3 days. The mothers later went in the communities and mobilized mothers with malnourished children, usually not more than 12 mothers and that they coached in teams.

**Q: How often did the role model mothers mentor the mothers with malnourished children?**

**R**: The mentorships lasted for a maximum of 6 months. But it greatly worked. The program in general was active for 3 years (2016 to 2018).

**Q: How do you perceive the utility of these programs and policies?**

**R**: PD Half was a very good program and policy. This is because we used locally sourced foods and labour to implement it so, it was community centred which made it gain a lot of impact in the community. So, it is great theory that works because we have seen reduced cases of malnourished children here in Mayuge district because it focused on using locally available nutritious foods which also makes it sustainable.

**Q: How did you target the right population to participate in this program?**

**R**: We did a nutrition assessment of the children that needed help with improving their children.

**Q: How did you make the people in the various communities aware of this program?**

**R:** We did nutrition counselling of the guardians with malnourished children and greatly convinced them to join our program. Some parents accepted and the others refused. But after seeing a great improvement in the physical health of the children in our program that were being fed. The parents that had initially refused to participate also decided to join the program. The mothers to malnourished children that had gained from this program also started to mobilize their fellow parents to join the program and benefit from it. So, mobilization was mainly by the peers, VHTs and community leaders.

**Q: What other outcomes did you achieve out of this program?**

**R:** Over time, the fathers also started to attend these PD Half programs and through them they started to see particular food crops that they were not producing at their homes lie dodo. Through the parents interactions in this program, parents started planting and producing nutritious crops at their home that they had seen from other parents in this program. This aided us to buffer the local production of nutritious foods. Additionally, the program aided to dispel perceptions of some parents who earlier though malnutrition was as a result of witchcraft. Generally, we achieved the agenda of promotion of food nutrition in Mayuge though not on a very large scale.

**Q: In your opinion, do you think the PD Half program achieved it desired objectives?**

**R**: Yes. Much as it was not 100%, atheist we achieved over 70% success.

**Q: What were some of the unintended consequences of this PD Half program?**

**R:** The defaulting of some mothers from coming for more training sessions hindered the effective implementation of the program. That was the only unintended consequence, the rest of the program was largely a success.

**Q: What are the challenges you met during the implementation of this PD Half project?**

**R**: The issues of peer mothers providing their own food for the nutrition training sessions prevented some mothers from completing the 14 days of training due to lack of resources.

**Q: In regard to the issue of the 18-year-old girl who was malnourished that you talked about earlier, what was done to solve her problem?**

**R**: Initially this girl had been misguided by her peers to eat less so that she can have a great figure and become more attractive. Based on this bad advice she stopped eating vital meals like lunch and would even go the whole day without eating anything. This led to her malnutrition. After her diagnosis, she was admitted and fed on nutritious supplements and she later started improving and becoming a better.

**Q: Can you think of any program that can brought on board to improve nutrition among adolescents and young adults?**

**R**: In Mayuge we have abundance of locally produced nutritious food the only problem is the knowledge gap. I ideal program to solve this problem would be on that aims to teach the mothers or caretakers here in Mayuge to effectively prepare the readily available local nutritious food to realise the intended nutrition benefits. There needs to be a program that champions the sensitization of adolescents to eat a balanced diet. This program should also focus on the caretakers especially the mothers who decide and prepare what is eaten at home most of the times.

**Q: Who do you think is the best person to implement this program you are proposing and why?**

**R**: The best place to implement this program that focuses on nutrition literacy would be schools since most adolescents are in schools and also the school environment can aid us have practical illustration of these trainings. So, the teachers in these schools are vital people in the implementation of this program because these teachers spend more time with these students.

**P: What about the adolescents that are out of school?**

**R**: The best people to teach these adolescents about nutrition would be their care takers. That is why caretakers are vital in the implementation of this program. But there are very adolescents at home, most of them are at school.

**Q: How frequent should this nutrition literacy program be delivered?**

**R**: In my onionin, I believe this nutrition literacy program should be included in the school curriculum. For example, we can start teaching it as a subject or part of a broader subject like science as early as Primary Four. The frequency can be yearly according to the age of the adolescent.

**Q: In terms of nutrition literacy, how differently would you like this to be dome in schools.?**

**R**: The topic of nutrition is being taught in schools though at a very low and limited level. So, there is need to incorporate more information about nutrition education in the school curriculum and most importantly provide practical classes to demonstrate what is being taught theoretically in school. There is need for demonstration classes at these schools to buffer the practical bit of the nutrition education.

**Q: What kind of resources would be needed to implement this program?**

**R:** We mainly need well trained teachers to pass on this nutrition knowledge to the children. Secondly, we need finances to use in setting up these demonstration gardens. The two will be very vital for the success of this program. The only problem here in Mayuge is that we have few organisations interested in nutrition education, that is mainly World Vision and Nutrition Component and Uganda Life Activities.

**Q: What challenges do you envision to be encountered during the implementation of this proposed program?**

**R**: Lack of funds mainly.

**Q: How can this challenge be overcome?**

**R**: There is need for effective utilisation of available resources like the UPE fund and PHC at the health facilities. The other source of funds can be through lobbying from like-minded organizations like World Vision and other NGOs as I had earlier mentioned.

**Q: Is there any other thing regarding nutrition of adolescents and young adults that we have not talked about that you would like to shade more light on?**

**R**: Improving the nutrition status of adolescents is easy since this age group has a very high appetite for food. There is also need to also control these adolescents regarding what they are eating because sometimes eating everything one lands can lead to obesity, which is also not admirable.

**I: Thank you very much for your insightful views and time sir. I am very grateful.**

**KII_Mayuge District Education Staff**

**Socio-Demographic of the Participant**

| **Age** | **45 years** |
| --- | --- |
| **Job title** | **XXXXxxx** |
| **Working Experience** | **2 years** |

Interviewer(I)

**I: So regarding nutrition, what are the major concerns regarding nutrition and diet among adolescents in Mayuge district in this context we are looking at those in schools?**

R: Issues concerning nutrition in our schools what I have seen is that our children in schools are not getting what they are required of them to get as far as nutrition is concerned because this is whereby… you know like provision of meals at school the children are not getting what they are supposed to get while at school like parents developed this thing of providing even the little as far as the meals are concerned with the children and those who are trying to do it they are just managing to feed them on porridge… really porridge and not this other type of porridge which is nutritious because remember those days we used to have porridge whereby they don’t remove the coating they just grind it and that was way nutritious by then, today the learners I don’t know maybe because of the changes that are taking place and there are the changes that we are putting you find that even those who are trying to eat porridge at school it is not even nutritious and fairly porridge… others are not even managing to eat beans and posho so nutrition in our schools for sure it is not there because children are running with hungry stomachs in schools whereby if a child is not fed that is why you see those other issues are now coming in things like ulcers are coming in, things like diabetes are coming in because our children are not eating at schools in summary that is what I have to tell you schools are not feeding the children the parents have given up… the community involvement; parents and community involvement in education issues it is really very weak thus leading to poor community participation…

**I: I think that answers part of the next question because I wanted to ask you that what do you perceive to be the biggest barriers to health diets among adolescents in Mayuge District but maybe you may have an addition in addition to the fact that they are not feeding, what are some of the barriers that are leading them not to eat?**

R: You see now… you know our people I want to give you some little background a bit, when UPE was introduced we are all aware that UPE was a very good policy when it was introduced so whereby they were saying that 4 children were to be supported by government and those who had more than 4 would now take it up on his or her own but later soon or later the policy changed you know in Busoga here people want free things you are also aware about it and now when you compare for example I want to give this an example, I went for a study tour in western Uganda and the children in schools are feeding on Matooke and they are having milk tea like they are eating well and in some other school they were even giving them eggs which is not the case this side so when UPE was introduced people turned this to free education and indeed even the government decided to take it like that and with free education people thought that everything is free that is why you see that the provision of learning materials to the learners (scholastic materials for the learners) parents are not buying books for their children a child leaves home and goes to school without a book, without a pencil, a pen and without a mathematical set so there is that… I don’t want to qualify it to be a political interference but somehow somewhere it is as a barrier sometimes when pronouncements are made by the president that low payments of that nature people thought that even feeding their children they should not feed them so the payments are not there and I don’t also believe in payments but if they are making payments to feed their children that is okay to me which parents don’t want to do so the politicians come in because of their personal gains they come in and confuse the parents like for example in Mayuge here we touch the policy because we want to have an audience but we realized that an audience will take long so we decided to pass the feeding policy in schools. The IPs have come in to help us enforce the feeding policy in schools but they have also failed so even the feeding policy that we passed which was a council resolution to have a feeding policy is not working because of the interferences which are political sometimes people bring in the economic and we are not well we are not okay even feeding their own child as if when the child is at home they cannot feed so those are some of the things but the major barrier is political and some people have… misinterpretation of the political pronouncements has caused us issues because people have misinterpreted when the president says there should not be this then you find people like “but why are you telling us to feed children, the president said so” and it is fine but the president said that you can organize in your local area to see what works for you but for real if the children are only fed on porridge really is the porridge nutritious…

R: But also, the availability of food in a given season largely determines what adolescents eat. Their diet changes with the season

**I: It is not but now I want you to elaborate more about your feeding policy and any other mechanism that you have come up with in line with feeding the adolescents in schools.**

R: When you talk about the feeding policy, the resolutions were made within that policy saying that one; all schools had been mandated to feed their children in schools whereby any parent found not contributing towards the feeding of their children would be reprimanded that was the first resolution which was made and like I said that now even when we were like let us involve the education committee… the education committee took it up and they said no the education committee should move to the villages to mobilize people, sensitize people and tell them the good that is in… and the reason as to why it is good to feed their children while they are at school so the education committee that political method started moving out schools to mobilize parents and to inform parents as a way of retaining learners in school feeding them is one of the ways of course telling them a healthy mind leads to a healthy body that can be promoted by feeding the learners literally that was what was involved in our feeding policy which was passed on 22^nd^ of December, 2023 that is when the policy was passed by the country so we are yet to get there. Yes, the introduction of the policy there are some who have started feeding and others are still I school but of course it is just a grain of salt in a lake it is a very small number of those who are trying to do it the majority has not really taken it up and some schools have reached the extent of even forcing the learners not to go home during lunch time but skillfully the children still go home because definitely when hunger approaches you, you cannot resist you have to find solutions on how to go about that.

**I: So going forward, what determinants shape up the dietary choices of these adolescents in schools now that the porridge is not given even the parental support is little even when there is a feeding policy what is shaping up their choices of what they eat?**

R: That one now goes back to… sometimes I don’t want to say this but you know when you look at the family background sometimes it causes issues of that nature for example when you go to the Banyole…Among the ethnic groups in this area, the Banyole mostly eat millet bread; the Basoga prefer cassava and sweet potatoes; while the few Baganda living here tend to eat matooke. Young people in Mayuge generally follow these culturally rooted food preferences.

**I: So which role do you think does the lack of certain food options in adolescents’ environment play in discouraging them to eat healthy diets?**

R: Yes, it does and yes it doesn’t like it is two-way and I say yes because when you go to some other areas you will find people speaking in point to what they need and their interests you cannot force them to go by what other communities are going with and yet in other areas no because it cuts across others use the cross sectional approach they say no whatever comes to us we can go by it for example is Malongo sub county and Bugono sub county people have now developed the element of growing Matooke and yet Bugono was full of the yaps but now for them they have started even growing Matooke in Bugono so you find that in Bugono there is Kalo for example there is a mixture of those issues they grow Matooke and they millet bread, they grow posho, they have cassava so that is one of the sub counties that at least I know which is trying to have a variety of foods in order to promote a balanced diet to ensure that our learners can or those adolescents can go well by the way do you know that even the diet even affects menopause of the girl child and I didn’t know that before so it affects either positively or negatively and there are those people who are eating… there is a place where I went to it was a school and the children were having difficulty in easing themselves… whether they were eating millet bread or cassava I didn’t know that it also causes issues so the head teacher was wondering what had happened to the children whenever the children would go to ease themselves they would make noise and they would shout and cry and the cause was they were eating millet bread not mixed with cassava.

**I: Other than the feeding program I school, is there any other program designed to promote healthy eating among adolescents in schools at district level?**

R: Yes, there is because we have now tried to encourage some schools those that have some land to start planting… in fact our production officer the district production officer has encouraged to start having school gardens whereby in there they can have Matooke being planted, they can have pineapples and those other demonstration gardens especially when it comes to agricultural lessons in fact the district agricultural officer and the production officer are helping our schools all those schools which are having some land are being encouraged to open up some gardens such that they can supplement all that they have and I have heard of some schools one there they had a garden and it was opened only that care also matters a lot I think of late it is not doing well then we have schools in Kigandaalo who are having gardens, we have schools in Malongo having school gardens having school gardens where they have planted Matooke and they are trying to put the cassava so that is one of the things that are being done at least encouraging these schools having some land to plant some crops.

**I: So who are the primary beneficiaries from the school gardens are they the adolescents or the teachers?**

R: Now those ones having big ones are teachers and adolescents both of these are benefiting but mainly teachers because that one we have to be sincere the adolescents are not considered so much…

**I: So the teachers are the primary beneficiaries and the adolescents are the secondary beneficiaries!**

R: You are right.

**I: So are these gardens delivered by the agricultural officers or by the teachers or by the adolescents at school?**

R: No, the agricultural officer gives the training and gives the encouragements to school to start opening up those gardens so the schools take it up as they are being guided by the agricultural officer.

**I: Is it at every level of the school that has or those ones some have the gardens and some don’t have?**

R: Yes, definitely others have and others don’t have even the fruit trees are being planted in schools though sometimes the communities which are hostile they sometimes end up spoiling what they have put up you know these communities are funny somebody plants the fruit trees then they take their goats and their other animals to destroy because sometimes we have those people who are taking the animals to the school compounds and they end up spoiling these trees like guavas some schools have planted guavas, oranges, mango trees but we also have issues in fact the operation wealth creation gave schools these trees so those who are serious are having them and those that are not serious are not having them.

**I: So I have noted three programs, one there is a feeding program, there is a program of having school gardens and also a program of planting fruit trees… is there any other program that is targeting nutrition that I have not noted?**

R: No.

**I: So now that takes me to another question that how do you perceive the utility of those programs do you think they are well utilized by the right populations, are they addressing the needs like what is your general comment?**

R: Those interventions are trying to address the issue that we have but the challenge we have that is why I said that some have and some don’t have, if one is having a fixed mindset which else leads to positive attitude because negative attitude leads to a fixed mindset for those who are having a positive attitude which leads to growth mindset things are working well and those who believe in starting small grow big and they are also doing well but you find that our people also sometimes the head teacher who is resistant to change might end up saying no but now I have to go I am here for two or three years so I might be transferred why do they bother with me if the community is not positive especially those communities which are hostile sometimes head teachers give up quickly so they be like I don’t want to bother myself with this so you see that so those are some of the moments which I have seen there.

**I: As we are about to end this discussion, can you think of any programs that could complement or replace the already existing ones in line with improving nutrition?**

R: I want to tell you that the nutrition program was introduced in some other district but in Mayuge it was not introduced here… I know of districts like Namutumba it was introduced in Namutumba but with us here we had not been supported fully to that capacity I want to say that even the people who would go in to give support to our people in terms of… when we talk about nutrition that is written about because the communities have not been sensitized meaning this is between me and you I know when you talk about nutrition I understand it because it is not all about eating meat, chicken… no it is more than that but even the greens which are more nutritious are not there so there is need for sensitization or training to make our people and the communities to be aware that what is causing some of these issues especially for example the non-communicable diseases is because of the ways they are being fed. The way we are feeding the children some of these issues are coming in as a result of that and it would be prudent if our communities are sensitized especially in the parents meeting, when we call the parents in our schools I think nutrition should be key and should be one of the things on the agenda to talk about but it will require somebody who is an expert in knowing what nutrition is and how it does support the growth of the children. It is not just a matter of somebody talking about it and then it ends there. I f we can have such slots for example we can have even our people the head teachers when we talk about nutrition they don’t understand it to a certain extent they might understand but they may not know it in depth so if we can have such meetings where we can have the head teachers maybe we can have the management committee members in zones or maybe in constituencies to me I think we shall have diminished a certain problem somewhere.

**I: Okay, this is very good submission… are there challenges you anticipate that might be encountered during sensitization?**

R: I don’t think so, maybe time because if… for example I called for a head teacher’s meeting to talk about issues we would have a slot of somebody who is very much aware about nutrition to come and talk about it. We can give a slot of like 30 minutes for somebody to talk about nutrition, what it is all about and what we need in our schools and how can we help the children. We can even go to the extent of encouraging the children in their homes to start putting a few things in their homes like there is where I went in Bushenyi can you imagine children are having gardens in their homes with some plants I saw it but they got the idea from school and they went and planted avocadoes in their homes but today we even have people who don’t know the importance of having avocado trees in a home yet we even have them in our schools because avocado is something very nutritious and those things have gone my brother by the time we were in school we used to have those things in schools but today even communities like for example most of our schools are either church founded or Muslim founded so these people the management have hired out lands to the sugarcane growers instead of giving the school that land so that the school uses it but they have hired out none and yet at the end of the story they say the school is under their foundation so it is their school but they are not even supporting the school because if they gave the land to the school people would be planting nutritious crops on the land so if head teachers are sensitized and the management committee members also on how nutrition is affecting our adolescents today for me I would think we shall be good to go. Now that those schools the children are trained the children go home and transcribe what they have learnt at school to their homes we would encourage these children to at least plant an avocado tree in their homes they would get all that from school so there is that bit of relaxing in doing some of those things.

**I: Okay, unless when there is any addition but this has been so informative I want to thank you for your information, for your time and everything.**

R: You are welcome sir.

**I: Thank you so much.**

**END**

**KII_CSO_MAYUGE**

**Socio-Demographic of the Participant**

| **Age** | **26 years** |
| --- | --- |
| **Job title** | **XXXXX** |

**Interviewer(I)**

**I: Thank you for giving me this opportunity to speak to you I just need you to introduce yourself briefly and tell me about your organization then we go on.**

R: Yeah my name is ……. and I am a fellow working with XXXX (CSO) (full organization name withheld) in the eastern region of Uganda that is Mayuge district and we look at quality education and we also do various projects that support nutrition education within school and so these projects require us to do for as long as we have to identify a project that suits within the area in which we operate and that helps us to solve the challenges that we find within that place yeah and for this case maybe depending on what I shared with you is that personally I found there is a challenge to do with meals for the learners and challenges to do with the malnutrition there is no suitable food program for the learners with in the school and the things that happen at home because some learners don’t have breakfast or even lunch at school so that affects their way of learning and so I came up with a small solution which I thought overtime it is going to be a bigger project.

R: Okay, now XXXX (CSO) is another invention like I said that it deals in offering quality education in low income societies and basically they train fellows who are in simple terms teachers like we are not specifically teachers but we are trained to do the work of teachers but given various trainings that can help the learners much better on their output so with those trainings they help us to be able to also be supportive to government teachers who are already in place and then to come up with various projects that can help support the wellbeing of the learners like T4G also offers digital learning opportunities like it offers the projectors and what I am talking about these are local schools and extreme village schools but they are offered with such things so that they look like they are meant for the village so when learners use projectors, when they complete studying and maybe they move to a modern area or to town they will not look like they have never seen this thing before they will be like at least my teacher has ever taught me using this thing and they know it and it is called a projector so the intention is for them to learn equally like those who are in Kampala.

**I: Where are you based as** XXXX (CSO)**?**

R: The headquarters are in Muyenga and we have a streamlined kind of administration where the CEO or maybe the executive director and the managing director both sit in Kampala plus a team that sits in Kampala but in field it is what we call fellows and me I am a fellow in a specific school.

**I: Do you have an office in Mayuge as** XXXX (CSO)**?**

R: What we have are the supervisors who live within… the supervisors we call leadership development officers and those are the people who supervise the work we do then on top of the leadership development officers we have what we call the senior program officer that one is the regional coordinator for example in eastern Uganda where I am we have around 6 districts we have Mayuge, we have Namayingo, we have Bugiri and we have Buikwe and we also have Namutumba…

**I: So the leadership development officer is allocated to a district…**

R: Yeah to a district so we have the leadership development officer within a district so whatever district I have mentioned has a leadership development officer then the district development officer reports to the Senior program officer that is the regional office then the senior program officer reports to the head of programs in Kampala.

**I: I understand. So how many schools are you attached to?**

R: No me I am attached to one school and the reason I am attached to that school is that we are only attached to a school for a period of two years and the reason we are attached there is that we expect that within that period you have coped up with the environment of the school and you have gone ahead to know the people you are living with and they are able to identify certain challenges within your community and then in your second year of fellowship in the Teach Uganda fellowship you come up with a project that identifies the largest number of challenges that affect within that community or within the school because you either do a community impact project or a school impact project depending on what you have identified as per but most importantly it should be helping the school…

**I: I understand so now that takes me to one of the questions, what are the major concerns regarding nutrition and diet among adolescents in Mayuge schools?**

R: For the time I have spent, one is that there are challenges to do with meals and when I talk of meals I mean timely meals… the learner I have interacted with that is both the young and those ones in upper classes that is from P1 to P7 there are challenges to do with meals and the meals are not timely there are learners who come from home where there are no meals in the morning and that learner come to school to study and then there are those who even come to school and they have not paid like you know that contribution towards their meals and so that means they also will not have meals at school because that one is for those who have paid. So it is quite challenging and that is one of the areas that I can look into…

**I: So one of the major concerns is meals is there any other concern?**

R: Then in the social work language in which I am I will call it poverty because the challenge is that you will find a parent has 8 children and among the 8 children 4 are going to school and those 4 children need a certain contribution of school fees and on top of school fees they need to eat so you find parents they neglect certain roles and they say that if I am able to provide food at home maybe a meal a day and actually in our organization we do what we call home visits I have visited some homes and I have seen young children who are malnourished because of the way how they feed you will find a child feeding on cassava throughout and then it leads to a certain form of… I have been to different homes and sometimes I mean this keeps us from going to certain homes because some of these people are violent so you restrict yourselves because you might go to a home where there are challenges and you fail to… and that one also affects us in terms of trying to find out.

**I: So, what do you perceive to be the biggest barriers to healthy diet among adolescents in Mayuge?**

R: Now what I would perceive is firstly the parental nudism because I cannot blame a school because all these adolescents or learners come from their homes to the school and the role of the teachers is to make sure that these learners get to understand what they are teaching hence that is one of the things I would say then secondly I can term it as laziness of parents you will find out that some parents don’t actually put a lot of concern on how the children feed they just say for as long as I have provided cassava that is food and my children can have that and then maybe three I am trying to find a way of rephrasing it… okay I can bring it this way that parents concentrate a lot on income generating agriculture rather than healthy diet because you find a parent might have a very large portion of land and then maybe because this one of the key issues is that most parents grow sugarcanes so they will use the largest portion of land to grow sugarcanes instead of planning for one acre to try and use it to plant rice and then plant here cassava and then maize there because food can never be enough because sometimes it will disturb them so you find that they cannot keep out enough food for themselves so they concentrate a lot more on income generating agriculture rather than healthy diet for their families.

**I: Can you think of anyways how we can overcome these barriers?**

R: I had first come up with a few solutions which actually they are not yet fully complete and one was engagement with school administration on how we can… my suggestion was like for example there are schools which have large portions of land and we could designate a certain portion of land for example if we plant maize within a period it means in the next term if we are able to harvest very well we can get back our seeds then the rest of the harvest could be taken to the machine to be grinded and then that would be the first installation for the learners to have a feeding scheme at school then also we want to have sensitization with the community and the community here we have to inform them that in spite of them needing money there is also need for health so in this case we wanted to come with a strategy of informing the parents that you see if you have a certain portion of land let us make sure you raise a 50-50 balance like 50% can take care of you family and 50% would be income generating and then lastly which is also an implemented plan already in my school, we intend to have more of vegetables and fruit trees and this is a long term goal but at least if a school can have more than 200 fruit seedlings of different kinds, then it can be a plan that also be helpful and for it, it can be continuous overtime that at least learners can find something to eat on the school premises like when there is a mango tree, there is a pawpaw tree, there is an avocado tree, there is a… for example in my school we have jack fruit and if all those trees are within the school community it means… for example if a learner is hungry and they can see a mango tree outside in the compound and picked a mango and eat and at lunch time they can maybe move out and see maybe a jack fruit and they said let me have the jack fruit for lunch I will get back home and find something it means we shall have the problem half solved but at least we would know that if a learner comes to school at least they have something to eat and then we want to incorporate this in the community. We had a training on nursery bed establishment for both fruit seedlings and indigenous trees which intended that we help parents to know how to create their own seedlings and they can plant more seedlings within their homes which means this can be two-way it can be income generating and at the same time it can provide food for that particular home after we have given them that knowledge so we hope that if this is pushed it is going to be helpful to such…

**I: But I would want to ask you, what determinants shape up the dietary choices of adolescents in Mayuge?**

R: Number one is where they were raised, if they were raised in a home where they eat cassava and that is the food provided there is no way even if you are an adolescent or a young learner there is nothing you will do about it because that is where you are and that is… two it is also the available food like the food that is available for them if there was an option… this side there is an option for rice but there are parents who cannot afford because of the income generating so you find that the person will only give you cassava and that is what he will offer as a father so there are choices that cannot be done by specifically them except if maybe we… if I am to talk about the adolescents who are non-school going and maybe they are getting some little money it depends on where they are if they are working in sugarcane plantations that is what they will have and maybe if they have some money that is when they can afford to go to… but that is a percentage which I may calculate out of 100 we may have 5% who make their own money and the rest the 95% are dependents on their parents so most of them I don’t think they have their own decision to say that me I want to eat this yet your father… your father cannot bring cassava and you say that me I want ice yet it is what he has provided so that is what I can say that is hindering what I have seen…

**I: Do you think that level of indication plays an important role in what they base on to choose food?**

R: Yeah, that is true and my reasons to support that for example we have educated teachers who are teaching these things but I don’t think they will have their challenges because of the education level you will find that the person knows and you will find that a teacher has a program and it is done then they tell you that every Tuesday I have to buy fish for my family and so that tells you a lot because I have shared with a number of teachers and I have moved in a number of schools and in spite of this placed on one school I have visited quite a number of schools and more than 4 where I have been physically and still trying I have done this same approach of planting fruit trees in over five schools within Mayuge and that has given me a picture when I share with them you find that the challenges are the same, the problems are similar and they are trying to meet the same goal so if a person is educated I think there is a huge difference because it is the families where we have had these home visits are the ones that are having quite a number of challenges I think I do not want to blame them but because of the education levels… the person we have is not educated and they do not have sustainable income sources so you will find that they will only provide what they can and then maybe there is something that I forgot which I observed within the communities there is what we call polygamous marriages and actually early marriages so you will find now that you have very many families belonging to one person and that is why you are seeing there are many numbers of children and when you have very many numbers of children it is hard for you to take care of some of them so that one is also a cause and a challenge in the community.

**I: So on what information do you believe these adolescents are basing on to make their food choices?**

R: One; the interactions within the school community here where I come from there is one that I shared with and there was a time just when I was with a few learners having physical education lessons and one collapsed and when I took the child aside and I am like what is the problem then the child told me that at home it is compulsory for me to fast and it was during Ramadhan and then I asked him and told him you are at school you don’t need to be doing this and the child told me no, so he depicted a picture of the rules they follow and how they are treated at home so I gave the child something to eat and the child refused so I think should I call it religious norms or family… that come in that line. Then through the home visits we have made I have been in my community this is what I will maybe call random sampling because I live in Wandegeya primary school is located in Wandegeya B village and it is surrounded by Wandegeya A, Watundu, Kanyana… so those are families that are surrounding me and at least I have had a chance to visit all of them and you realize almost the same challenges that is why I said when you ask a question you find out that the challenges affecting one person are quite similar to the same because of the place within… how they have been living their life so I think they see it as normal. Ignorance, yeah let me use that so ignorance within the parents because… so that is also what I can say about that.

**I: So you have talked about this but maybe you could have something else to add on and the question says which role do you think does the availability of certain food options in adolescents’ environment play in encouraging a healthy diet?**

R: Okay, one is that it helps in their growth and also their mental capability… we had a scenario in the statistics of XXXX (CSO), Eastern Uganda has been one of the worst performing regions when it comes to academic performance and it is not just recorded on just specific… okay it is not just from the blue there are underlined challenges and so these challenges nutrition could be part of them so I think these adolescents could be given specific timely health diets it means it could also improve their mental capabilities to understand and give them a chance to give their education much more time.

**I: So do you think if there are different types of food around where the adolescents live this can help them to choose a healthy diet or you think differently?**

R: I think differently because when I came to Mayuge in Wandegeya community I found in the community there was only one jack fruit tree but I can assure with these seedlings being availed the learners would pick these fruits themselves and that shows a vision that those foods are within the community and it means they will still go for them the same with mangoes if you are to take the mango trees that have existed before you will not find the mango there when it is season for them so it means they will be able to have those specific mangoes so to me I would encourage you that it is about the availability because whenever there is access of the available foods it means they are available and it means there might be a difference and I would use a saying that says half a loaf is better than none.

**I: So we now go to programs, do you hold any kinds of programs that are implemented at district level that are promoting healthy eating among adolescents in Mayuge?**

R: At the district I have not seen any because I think if it was there then it should have already come maybe talk to us about that and then maybe share with us but I haven’t heard about it…

**I: So at sub-county level maybe where your school is, have you heard of any programs?**

R: No, I haven’t and that is why I have said if they were really there maybe there would be a communication and if I had heard about it because I am already working for them I think I would have got a chance to their and share with them that…

**I: Okay, since you have not heard of any we are about to finish… could you think of programs that could maybe come up to encourage adolescents to eat healthily?**

R: Now me I would suggest there should be a program called food and nutrition program for adolescent learners in both primary and secondary schools and these programs they target the adolescents that live between the age of 12 to at least 17 because why I am saying so in this some adolescents have not yet matured to take maybe their own personal decision and if they studied them it means it will make them to learn when they are still young and the activities of the program should be sensitized and then there could be visiting of certain areas that have done well in nutrition because if you take there the students it means that they could learn and you show them how things can be done and they should be done with everything so it helps them then the last activity could be physical engagement in terms of maternal provision especially for the things that can actually be bought those that can be provided if maybe there are trees they are going to help over the years it’ll be good that those trees are planted within and it should be designated areas and it should be these specific kind of trees that are specifically for nutrition so I think that is my say on that.

**I: So what challenges maybe encountered during implementing such a program?**

R: There are not so many and the challenges sometimes are the roads… the road network is quite challenging in some areas and that disturbs quite a lot but then there are schools that are across the lake… Lake Victoria I have been to the shores there at the area of the district of Mayuge but there are schools that are there so that means there is also a requirement of visiting them even if I have not been there I have a feeling that since they are situated in the same area they might need the same information which requires at least extra effort to transport by water transport to such areas and then for others I think the other challenges are quite not hard but I don’t think there is a challenge to do with that as long as you can reach the place then the other things can be manageable. Maybe what I forgot as a challenge could be lack of land for certain schools you might find for example my school is advantaged but it was given church land earlier before we came and it has around 8 hectares that are free and can be used for certain programs…

**I: But now how do we go about the lack of land as a challenge?**

R: Now that one it is an agreement between the parents… that needs an engagement between the school management committee and the PTA so that can be solved by them and then they come up with a solution together.

**I: Thank you so much for this information it has been a pleasure to interact with you.**

**END**

**KII_CSO STAFF**

**Interviewer(I)**

**I: Good evening XXX. My name is XX, a researcher from Makerere University, School of Public Health. I am part of a team examining young people’s nutrition and health in Uganda, specifically in Mayuge District, Eastern Uganda on Arise Nutrition Project. The information collected in this research will help us understand the foods adolescents eat and what determines the food choices among adolescents’ aged 10 to 24 years.**

**Q: What is your age?**

**R:** I am 33 years old.

**Q: What is your current job title?**

**R**: I work as a project officer with *[CSO name withheld]*.

**Q: How many years did you spend in school?**

**R**: 17 years

**Q: What is your current marital status?**

**R**: I am married.

**Q: What type of area do you stay in? Is urban, peri-urban or rural?**

**R**: I currently stay in a peri-urban area.

**Q: Do you have any disability?**

**R**: No.

**Q: What is your economic status? Consider these three variables; above average, average, below average.**

**R:** My economic status is average.

**Q: What issues or challenges do adolescents face in regard to nutrition or diet?**

**R**: Adolescents in most areas of Mayuge district usually do not balance their diet. In most cases they take one meal a day.

**Q: What do you think influences what adolescents eat? For example, how does hunger influence food choices of these adolescents.**

**R:** It affects them negatively because usually, when one is hungry, they pick what is around to eat and eat that.

**P: What about taste?**

**R**: Adolescents love tasty and oily things. They take things with a lot of salt, sugar. They generally prefer sweet things.

**P: Do you think sex influences food choices of these adolescents?**

**R:** Yes it does. In most communities, girls always prefer a specific kind of food and the boy prefer another. Most boys here in Busogo prefer posho and the girls usually prefer cassava and sweet potatoes.

**Q: Does ages influence the food choice of these adolescents. For example do we have choices where adolescents aged 10 prefer a specific kind of food and the ones aged 20 prefer another specific kind of food? Generally, do you think the there is a difference in food choices among these adolescents?**

**R**: Yes. For the young adolescents, the parents usually decide for them what to eat. For the adults, they easily decide for themselves. Most of the adolescents here are working, they do not child labor. So it is easier for working adolescents like those at 20 years to decide for themselves what to eat.

**Q: How does cost (price of food) influence food choices of the youth here in Mayuge District?**

**R**: Yes, the cost of food affects their food preferences. For example if the cost of a particular food is high, they can go for cheaper food.

**P: What the about the income they earn, or the one earned by their parents?**

**R:**  It also affects their food choices. Most of the adolescents and parents here in Busogo do not earn a high income, this therefore force the people here in Mayuge to demand cheaper food dishes.

**Q: How does education level influence what an adolescent eats?**

**R**: If someone is educated they always know a suitable diet to eat than one who is not educated. So, educated adolescents here in Mayuge make better food preferences than the undedicated ones because they learn a thing or two about diet in school.

**Q: How do cultural beliefs influence the food choices of adolescent here in Mayuge district?**

**R:** Tribes and clan also influence the food choice of adolescents. For the Buganda here usually do not eat some specific food stuffs due to the totems in the clans. So, some adolescents do not eat some food stuffs due to their clan limitations, however nutritious the food might be.

**Q: Does stress affect the affect the food choices of the adolescents here in Mayuge district as well.**

**R**: Yes it does. When an adolescent is stressed or working a lot, they rarely think about food and end up eating less often affecting the uptake of nutritious foods.

**Q: How does knowledge about nutritious foods influence food choices of most adolescents?**

**R:** The level of knowledge about nutritious food here in Mayuge is very low mainly among the adolescents. Most of the adolescents here usually just cook or eat any available food they are exposed to so, they do not balance their foods well. So, low level of knowledge about a balanced diet and nutritious foods to eat negatively affects the food choices of most of the adolescents here in Mayuge district. Additionally, most adolescents here in Mayuge still live with their families so, they normally eat what the family or parents have prepared. In most of the cases they have limited power of selecting the food they would like to eat. They eat whatever is available in the household.

**Q: How do cultural beliefs or myths affects the food choices of adolescents here in Mayuge district?**

**R:** The adolescents of these days dwell less on cultural beliefs or myths on food. They just eat whatever comes their way.

**Q: On what information do you think adolescents base on to make food choices?**

**R:** I think the source of information they base on is from within the household they are living in. So, they grow up knowing that they are supposed to eat this type of food and not necessarily the other type of food.

**P: What about the school going ones?**

**R:** The ones going to school get this information from school and health centers as well. We the implementing partners also teach these adolescents about different food types and nutrition to aid them have a balanced diet always.

**Q: What do you perceive as the biggest barriers to health diets among adolescents in Mayuge district?**

**R**: I believe it is lack of knowledge and information about these food diets.

**P: If they get this information about diets and nutrition, can they change and eat better food that is nutritious?**

**R**: At both the household and school level the capacity is there though it would require some time for the change in food choices to nutritious food to be realized. There need for demonstrations gardens at homes and in schools to aid in the teaching of these adolescents about better food choices.

**P: Is there any other barrier that prevents adolescents from eat healthy foods?**

**R**: There is also a problem of lack of land in Mayuge where families can grow their own nutritious food crops. Most of the land here in Mayuge has been rented out to sugarcane growers.

**Q: What can be done to reduce these barriers that are preventing the adolescents from eating nutritious foods and a balanced diet?**

R: There need to extensively share information about how the adolescents should go about their diets. This can mainly be done through sensitization. Secondly, there is need for demos where these youth spend most of their time for example homes, schools and heath centers where they can ne sensitized about the right foods to grow and eat. There is also need for a by-law here in Mayuge district that stipulates the required land that needs to remain for growth of food crops per household. This will prevent the practice of lending out all the land to sugarcane growers leaving not land to household to grow nutritious food crops.

**Q: What role does the availability of certain healthy food options play in encouraging adolescents to have a balanced diet?**

**R:** Yes, I think it plays a great role. If healthy food is readily available the youth will certainly eat it because they want to live longer. The kind of work they do also requires a lot of energy so; they need to balance their diet very well.

**Q: Does lack of certain healthy foods in a given community where adolescents stay discourage them from eating certain healthy foods?**

**R**: Yes. Some would never eat them since they are not readily available to them.

**Q: In terms of the available food not being healthy, how does that discourage them?**

**R**: Most of the adolescents here in Mayuge district do not know the difference between healthy and unhealthy foods. They just eat what is readily available. A few adolescents know about these differences between health and unhealthy foods.

**Q: What programs are being implemented in Mayuge district (National, district or county level) that is aimed at improving food diet and nutrition among adolescents?**

**R**: I haven’t heard of any government program to this regard. For my organization we have a component of good nutrition among adolescents under the program we are currently implementing. The project we are currently implementing is on child labor and there is a component where we are supporting the families where these child laborers are coming from with nutrition and balanced diet support programs to enable them become stronger and live longer during the time span of their work.

**P: In this project your organization is implementing, what exactly do you do to ensure that you improve their diet?**

**R**: We have sensitization on good feeding practices. Secondly, we also always do dialogues on good feeding with their care takers. We also encourage each household to have demonstration gardens that have nutritious food crops to aid in the provision of a balanced diet to the youth.

**P: Who are the primary beneficiaries of this program?**

**R:** The primary beneficiaries are the child labor victims.

**P: Who are the secondary beneficiaries?**

**R**: The care takers of these child laborers.

**Q: What is the age of child labor victims?**

**R**: It is averagely between 5 and 17 years depending on the legal framework being used. Because the children with 15 to 17 years are supposed to work under maximum supervision.

**P: Who delivers this program on ground and how often id it delivered or implemented?**

**R**: It is us the program officers and project assistants that implement the program. The demonstrations on healthy food are done weekly. The dialogues with care takers are done monthly.

**P: What is the coverage for this program?**

**R**: We implement this program in mainly sub-countries here in Mayuge district. Averagely, four parishes per sub-county. We also implement it in 22 villages.

**Q: How do you perceive the utility of these programs and policies? For example, this program you have talked about that aims to promote nutrition and balanced diet promotion among child laborers.**

**R**: Our target groups are positively taking these programs though on a rather slow pace. This is because mindset change takes time.

**Q: What do you do as an organization to target the beneficiaries for your programs and how do you vet these beneficiaries?**

**R**: Before we started the implementation of this program, we did a baseline study and mapped out beneficiaries according to already set criteria. After that we physically went on ground to verify and visit the families of our prospective beneficiaries to clearly ascertain the authenticity of our beneficiaries. Additionally, we went to sugar cane plantations and quarrying, mines where most of these children laborers work to assess their working conditions. This is the process that we used that aided us get the right beneficiaries for our program. We refrained from using local leaders because they are politicians and would sabotage the program by bringing in their voters or family members even if they do not have the right qualifications.

**Q: How do you ensure that most people get to know about your program?**

**R:** We usually do awareness campaigns with the help of LCs and VHTs who mobilize for us the people in their respective communities. We also use community radios to sensitize the people on the various components of our program. The other way we use is creating WhatsApp groups where we share massages to the youth and caretakers about the services being offered by our program. These are mainly the ways we use to ensure that people get to know about our programs.

**Q: How do you ensure that the target beneficiaries participate in this program you are currently implementing?**

**R**: We use local community association leaders to mobilize them for example those that lead village saving groups. Most women usually go for these saving groups meetings where we usually meet them in large groups and offer our services to our target beneficiaries.

**P: How else do you target your prospective beneficiaries in an event you do not easily meet them at local community gatherings like savings meetings?**

**R**: We also reschedule our community engagements to others in an event where our target population is not activity available. We also try to find out why they never participated in order to plan better.

**Q: What is the level of success or impact that you have achieved with this nutrition-oriented program that you are implementing in this community?**

**R**: For now, it is still a work in progress. Additionally, some of our beneficiary families have also been able to plan leafy vegetables at their respective homes as a result of our teachings on the use and need for vegetables to boost nutrition and balanced diet of these households. The only problem now is that the parents are sending children out to sell these vegetables on streets which is leading to more child labor and more of a challenge to us.

**Q: In your view do you believe the program has succeeded and met most of its intended results?**

**R**: The program has now only spent 7 months so I can not evaluate its success effectively at this stage but it will surely succeed.

**Q: How do you rate the level of success in implementing this program?**

**R**: As I has said earlier, the program is till too young to effectively rate but I am confident with time both the implementation and impact will attain great results.

**Q: What reasons are helping you succeed so far in your implementation of this program?**

**R**: The program is not so capital intensive, so, we have been able to provide our beneficiaries with adequate inputs to plant in their home gardens which are yielding fruit so far. Additionally, or beneficiaries are not incurring any of their money in participating in this program. This is encouraging them to continue participating in the program. More so, whenever our beneficiaries need our support, we are always readily available which is also making our implementation a success. We also do weekly follow ups and evaluations which are aiding us keep at pace with t impact and evaluation. We have also set up saving groups for the mothers and youth in our program. This has aided them to easily get access to finance their small vegetable farms for more growth and development.

**Q: What can your organization or any other organization should do better in order to improve the quality of the program about improving the nutritional diet of the youth in your community?**

**R**: I think it would be great for my organization to design nutrition programs that target households inclusively not just particular groups of people in the community. This will ensure that everyone is served because everyone in the community needs a healthy diet not only the adolescents so, an inclusive nutrition program for all I the community would be more ideal.

**P: Are you as an organization, are you able to implement such a broad and inclusive program?**

**R**: Yes.

**Q: Which other organization or entity do you think can effectively deliver an inclusive nutrition-based program targeting all the members of the community?**

**R:** The collective collaboration of all local development partners like NGOs and government institutions would be a great fit to implement this program.

**Q: If we are to implement this comprehensive nutrition-based program, what would be the ideal frequency engaging with the beneficiaries?**

**R**: It can be seasonal. For example, one a quarter in the financial year.

**Q: What resources are needed to implement such a program?**

**R**: The funds would be key and the well-trained personnel to effectively implement this program. Additionally, there is also need of inputs like seeds, pesticides, fertilizers and sometimes even land for the households that do not have land, this land may need to be hired.

**Q: I f you were to implement such a program, what are some of the challenges that you anticipate to encounter?**

**R**: Sometimes the community may not be willing to participate in the program which would sabotage the program. Additionally, there could arise other challenges like pests and diseases, and poor weather conditions like too much sunshine that might hinder the effective growth of the vegetables or other nutrition crops vegetable demonstration farms. The other challenge that might be encountered is limited funding mainly from donors for the case of locals NGOs like ours that heavily rely on donor funding. The political atmosphere may also hinder the success of the program for example some politicians may use or shun the project away for their own political interests especially if the project has been supported by the opponents. There is also an issue of aflatoxins mainly during the processing od these supposedly nutritious foods which end up leading to more diseases among the people that could have benefited from the project.

**Q: What solutions do you propose for these challenges stated above for the success of the program?**

**R:** There is need to involve local leaders in the implementation of the program for better community mobilization and sensitization. There is also need to start small in coverage and expand over time in order to be effective in the implementation phase of the program. For cultural rigidities, there is need for continuous sensitization so that they can be able to change with time. The crops or vegetables being introduced should also be locally known in order to increase their uptake in the community.

**I: Is there any other thing that you would like to share with me which you have not touched?**

**R**: No

**I: Thank you so much for your time and valuable insights.**

**END**
