# Supplementary material for: Socio-ecological factors influencing dietary behaviours among adolescents and young adults in rural Eastern Uganda: A qualitative study
Source: PLoS One. 2025 Dec 2;20(12):e0337797. doi: 10.1371/journal.pone.0337797 (PMC12671741; doi:10.1371/journal.pone.0337797)
Supplement: Attachment — Submitted filename: PLOS manuscripts.docx [file pone.0337797.s006.docx]

**Socio-ecological Factors Influencing Dietary Behaviors among Adolescents and Young Adults in Rural Eastern Uganda: A Qualitative Study**

**Review :**

This qualitative study explored factors associated with dietary choices among factor among adolescents in Eastern Uganda using the socioecological model. Data was collected cross-sectionally using both FGDS and KII over 3 months. The authors noted that rural youth favored the functionality of food over its nutritional value with uses ranging from physiological including getting energy to conduct manual labor-intensive activities, to emotional and social and suggested multilevel approaches to improve nutrition in this population.

This manuscript is very well structured and the scope is clear.

**Major comments**:

Line 93: Clarify Population based qualitative study, does this refer to your sampling frame being derived from the DHSS or the generalizability of your data

Line 94: Other than age and sex what informed your sampling frame for AYA and how does this frame reflect the earlier assertion of a population based qualitative study. Were participants selected from various regions in Mayuge. Were there other criteria informing sampling , how did the community leaders decide who to reach out to and recommend them for recruitment into the study or was this convenience sampling. Could you explain it was not possible to use a more robust sampling technique?

Line 113:Provide details on participant selection among the purposively selected participants. For example, were parents/guardians related to the AYA in the FGDS or were these just parents in the community, what informed their selection?

**Minor comments**

Line 72: “Only 23.6% consumed leafy vegetables/fruits 4+ times/week” ; Use sentence structure and replace symbols with words for proper flow. Do this across the document replacing symbols with words (except in the abstract) and removing unnecessary capitalization.

Line 120: Basket foot vendors is likely a contextually specific name, define it please or use a more common term.

Line 549-558: Is not supported by your results as the two people who spoke about this were KII and not AYA . It would be good to point out that this perception came from the adults unless you have an AYA quote backing up this opinion.

Line 579-581 “In contrast conducted in urban LMICs or HICs often have more decision-making power at the household level, influenced by media exposure, peer trends, and access to pocket money, such as in Zambia [26] and across Africa [49].” This statement has missing information, you might want to name the population you refer to for clarity.

Line 604: the word Uganda is missing

Line 73: Overall you have strong references but Sawyer ( Ref 18) should not be the primary reference in that introduction section as that publication is a review, replacing with a primary study would be more informative.
