# Supplementary material for: Socio-ecological factors influencing dietary behaviours among adolescents and young adults in rural Eastern Uganda: A qualitative study
Source: PLoS One. 2025 Dec 2;20(12):e0337797. doi: 10.1371/journal.pone.0337797 (PMC12671741; doi:10.1371/journal.pone.0337797)
Supplement: Attachment — Submitted filename: Response_to_reviewers.docx [file pone.0337797.s008.docx]

| **Reviewer comments - Authors’ response table** | | |
| --- | --- | --- |
| **Comment** | **Authors’ responses** | **Line numbers** |
| 1. Please ensure that your manuscript meets PLOS ONE's style requirements, including those for file naming. | We have carefully reviewed the PLOS ONE style requirements and made the necessary adjustments to ensure compliance, including correction of file naming conventions for the main manuscript and all supplementary files |  |
| In the online submission form, you indicated that “Due to confidentiality concerns and the potential for deductive disclosure in this qualitative dataset, the data are not publicly available. However, relevant portions of the de-identified excerpts may be shared by the corresponding author (TB) upon reasonable request and in line with the ethics approval conditions.”  All PLOS journals now require all data underlying the findings described in their manuscript to be freely available to other researchers, either 1. In a public repository, 2. Within the manuscript itself, or 3. Uploaded as supplementary information. This policy applies to all data except where public deposition would breach compliance with the protocol approved by your research ethics board. If your data cannot be made publicly available for ethical or legal reasons (e.g., public availability would compromise patient privacy), please explain your reasons on resubmission and your exemption request will be escalated for approval. | We have updated the data availability statement to indicate that all de-identified data analyzed for this manuscript are provided as **S4 File**, in line with PLOS ONE requirements. |  |
| Please amend either the abstract on the online submission form (via Edit Submission) or the abstract in the manuscript so that they are identical. | We have revised the submission so that the abstract in the online submission form and the abstract in the manuscript are now identical. | 17 – 47 |
| 4. We note that “Supplementary File 2.” in your submission contain copyrighted images. All PLOS content is published under the Creative Commons Attribution License (CC BY 4.0), which means that the manuscript, images, and Supporting Information files will be freely available online, and any third party is permitted to access, download, copy, distribute, and use these materials in any way, even commercially, with proper attribution. For more information, see our copyright guidelines: <http://journals.plos.org/plosone/s/licenses-and-copyright>.  We require you to either (1) present written permission from the copyright holder to publish these figures specifically under the CC BY 4.0 license, or (2) remove the figures from your submission | We have removed the copyrighted figures from **S2 File** as advised, to ensure compliance with PLOS ONE’s CC BY 4.0 licensing requirements. |  |
| **Reviewer 1** |  |  |
| **Comment** | **Authors’ Response** |  |
| This qualitative study explored factors associated with dietary choices among factor among adolescents in Eastern Uganda using the socioecological model. Data was collected cross-sectionally using both FGDS and KII over 3 months. The authors noted that rural youth favored the functionality of food over its nutritional value with uses ranging from physiological including getting energy to conduct manual labor-intensive activities, to emotional and social and suggested multilevel approaches to improve nutrition in this population.  This manuscript is very well structured and the scope is clear.  The qualitative results in this manuscript would help contextualize dietary choices among AYA in a rural region within an LMIC, making this research informative. The manuscript is well structured with a detailed and well contextualized introduction setting a strong basis for the scope and expected outcomes for the rest of the manuscript. However, the authors will need to improve the methods section by providing details on their sampling approach. Qualitative studies are not necessarily expected to be generalizable so subject selection must be systematic. Authors could add the justification for convenience sampling if that was the approach they chose to use. Further review details are below. | We thank the reviewer for this important observation. The study did not employ convenience sampling. Instead, **purposive sampling** was used to ensure inclusion of participants with diverse perspectives on AYAs’ dietary behaviors. Among AYAs, maximum variation was achieved by sex, age group, schooling status, and residence within the Iganga–Mayuge HDSS. Community council leaders supported mobilization but did not decide who participated; AYAs were directly approached at households and schools. Parents/guardians of purposively selected AYAs, teachers within the HDSS, and food vendors at schools were also included. This approach was deemed most appropriate for this exploratory qualitative study, where the objective was to capture in-depth details from the purposively selected respondents, rather than achieve statistical generalizability. | 110 - 133 |
| **Major comments** |  |  |
| Line 93: Clarify Population based qualitative study, does this refer to your sampling frame being derived from the DHSS or the generalizability of your data | To avoid ambiguity, we have revised the description and removed the term “population-based.” The study is now described simply as a qualitative study. This provides the intended context without implying generalizability. | 96 - 98 |
| Line 94: Other than age and sex what informed your sampling frame for AYA and how does this frame reflect the earlier assertion of a population based qualitative study. Were participants selected from various regions in Mayuge. Were there other criteria informing sampling, how did the community leaders decide who to reach out to and recommend them for recruitment into the study or was this convenience sampling. Could you explain if was not possible to use a more robust sampling technique? | We have clarified that, in addition to age and sex, variation in schooling status (in-school and out-of-school) and residence within the Iganga–Mayuge HDSS catchment informed the selection of AYAs. Recruitment occurred in multiple settings: out-of-school AYAs and parents were approached through household visits, and in-school AYAs, basket food vendors, and teachers during school visits, with community leaders providing guidance for mobilization but not determining participation. District staff and CSO officials were recruited at their workplace offices. We further emphasize that purposive sampling was selected as the most appropriate strategy for this exploratory qualitative study. | 111 - 125 |
| Line 113: Provide details on participant selection among the purposively selected participants. For example, were parents/guardians related to the AYA in the FGDS or were these just parents in the community, what informed their selection? | Parents/guardians of purposively selected AYAs were recruited to provide complementary perspectives on adolescent dietary practices at the household level. Teachers were recruited from schools within the Iganga–Mayuge HDSS where some AYAs were enrolled, while food vendors from these schools were included given their routine interactions with adolescents through the sale of ready-to-eat snacks. District- and community-level stakeholders (district education officer, nutrition focal person, and CSO representatives) were selected based on their direct involvement in adolescent health and nutrition programming. This clarified selection ensured that all participants contributed relevant and context-specific experiences. | 121- 125 |
| **Minor comments** |  |  |
| Line 72: “Only 23.6% consumed leafy vegetables/fruits 4+ times/week” ; Use sentence structure and replace symbols with words for proper flow. Do this across the document replacing symbols with words (except in the abstract) and removing unnecessary capitalization. | We appreciate your suggestion regarding sentence structure and the replacement of symbols with words to improve readability. We have revised the manuscript accordingly, replacing symbols such as “/” and “+” etc. (e.g., “four or more times per week”) and removing unnecessary capitalization.  With respect to the percent symbol (%), it appears more than 20 times in our main text, and converting all instances to “percent” may introduce redundancy and reduce readability. We also note that a recent PLOS ONE article (published July 28, 2025) consistently uses “%” in the main text; for example, “over 40 % tree shade coverage accounted for 76.22 % of the total” (<https://journals.plos.org/plosone/article?id=10.1371%2Fjournal.pone.0329278>). For this reason, we suggest retaining the “%”.  We are, however, happy to make this adjustment should the Editorial team deem it necessary | Through-out |
| Line 120: Basket foot vendors is likely a contextually specific name, define it please or use a more common term. | To enhance clarity and contextual understanding, we have defined the term in the methods section. In the rural Uganda context, *basket food vendors* *are* ***community members who carry baskets containing ready-to-eat foods such as fried cassava, roasted groundnuts, fruits, etc., and sell them to students around schools.*** | 111 - 113 |
| Line 549-558: Is not supported by your results as the two people who spoke about this were KII and not AYA. It would be good to point out that this perception came from the adults unless you have an AYA quote backing up this opinion. | We have revised the text to clarify that the perception linking food and sexual attractiveness were voiced commonly by **teachers and district staff, who were included in our study as stakeholders with influence on adolescents’ health and nutrition**. This framing better reflects the intention behind our sampling and ensures the discussion accurately represents the sources of this perception. However, we have also iteratively reviewed the transcripts again, re-analyzed data and were able to obtain additional relevant quotes from AYAs. | 331-342, 589 - 602 |
| Line 579-581 “In contrast conducted in urban LMICs or HICs often have more decision-making power at the household level, influenced by media exposure, peer trends, and access to pocket money, such as in Zambia [26] and across Africa [49].” This statement has missing information, you might want to name the population you refer to for clarity. | We have clarified the population referred to by specifying that it is adolescents and young adults who are reported to have greater decision-making power at the household level in urban LMIC and HIC contexts. | 605 - 607 |
| Line 604: the word Uganda is missing | We have revised by adding the missing word, “Uganda” | 618 - 619 |
| Line 73: Overall you have strong references but Sawyer (Ref 18) should not be the primary reference in that introduction section as that publication is a review, replacing with a primary study would be more informative. | We have replaced Sawyer et al. (2012), which is a review, with primary empirical studies that directly examine the impact of poor dietary patterns on adolescent growth and development. Specifically, we now cite Caleyachetty et al. (2018), a multi-country study analyzing data from over 129,000 adolescents in 57 LMICs; Martínez Arroyo et al. (2020), a longitudinal cohort study showing that poor dietary quality contributes to growth and obesity risk; and the National Longitudinal Study of Adolescent to Adult Health (Add Health), which demonstrates long-term associations between adolescent diet and weight-related outcomes in adulthood. Together, these provide robust primary evidence to support our statement. | 76 - 78 |
| **Reviewer 2** |  |  |
| **Comment** | **Authors’ Response** |  |
| Line 53-55: The logical progression in this section is unclear. The statement that “changes that come along with the transition from adolescence to adulthood make individuals more susceptible to emotional and social factors” requires further clarification and supporting evidence. It is not explained which specific changes are being referred to, or the mechanism by which these changes increase susceptibility. Furthermore, the link between such susceptibility and a tendency to favour short-term over long-term benefits is asserted but not sufficiently justified. Please consider elaborating on these points and providing references to support the proposed causal relationships. | we have revised the section in the introduction to improve logical flow and provide clearer justification. We now clarify that adolescence is a period where increased nutritional requirements coincide with rapid physical and cognitive maturation, while social and developmental changes heighten susceptibility to external influences. We further specify that such influences include peer norms and social approval, which contribute to adolescents’ tendency to prioritize immediate rewards over long-term health benefits. To strengthen this argument, we have added references to Steinberg and Morris (2001), Steinberg (2010), and Somerville et al. (2010), which provide empirical and theoretical support for these developmental mechanisms. | 52 -59 |
| Line 68: It would be helpful to briefly clarify what is meant by “dietary diversity” for readers less familiar with the term. | We have added information on how low dietary diversity was operationally defined in the study we cited (Isabirye et al., 2020). Specifically, the revised sentence now reads: “In 2020, among 498 adolescents in Iganga and Mayuge in Eastern Uganda, 45.3% had low dietary diversity (consumed fewer than four of the nine standard food groups in the past 24 hours), while 87% consumed fats or oils (Isabirye et al., 2020).” This revision provides clarity for readers who may be less familiar with the concept. | 69 - 71 |
| Line 388: Quote is missing. | We have carefully cross-checked the transcripts and confirm that there was no missing quote in this section. The information previously shown in brackets (“FGD, mother Teachers, Secondary School”) was inadvertently placed in error and has now been removed. The revised text now retains only the verified quotes and their accurate attributions. | 401 - 414 |
| **Line 635: General comments:** |  |  |
| Highlight the novelty and rural specificity: Make it clearer which findings are novel or distinctive to Mayuge, and how they extend or differ from what is already known from urban SSA or HIC contexts. This will help bridge the current evidence gap on rural settings. | We revised the discussion to consistently highlight rural-specific findings and their novelty. For example, we emphasized how satiety-driven choices, parental dominance, and seasonal food dynamics are distinctive to rural Mayuge in Eastern Uganda and differ from urban SSA or HIC contexts where autonomy, convenience, and commercial food environments play a larger role. These revisions explicitly situate our findings as rare empirical evidence from a rural context, bridging an important evidence gap. | 553 – 563, 564 - 638 |
| Clarify expected differences: When comparing rural findings to urban SSA or HIC studies, briefly explain where differences are expected, for example, due to variations in income, infrastructure, or food environments. | We expanded our comparative statements to explain why rural findings diverge from urban or HIC contexts. For instance, we noted that limited income and reliance on subsistence farming in rural Uganda constrain adolescents’ autonomy, in contrast to urban settings where access to pocket money and exposure to commercial food environments grant adolescents greater decision-making power. These expected differences are now explicitly linked to structural and contextual variations. | 553 – 563, 571 - 575, 577 – 583, 603 - 605 |
| Make subgroup patterns more explicit: Subgroup differences are hinted at but not systematically presented. It would strengthen the discussion to explicitly outline patterns by gender, in-school vs. out-of-school status, and adolescents vs. young adults. | We have rewritten results and discussion sections to explicitly expand subgroup patterns, highlighting, for example, that in-school AYAs relied on teachers for nutrition knowledge while out-of-school adolescents showed limited understanding of dietary diversity. Gendered norms were detailed, such as how food taboos disproportionately restricted girls. Differences between adolescents and young adults were also clarified in relation to autonomy and body image perceptions. | 247 – 549, 562 - 638 |
| Elaborate more on cross-level interactions: Some important intersections are mentioned but could be more fully developed, for example: Seasonality amplifying satiety-driven choices; Peer norms combining with pocket money to shape purchases; gender norms interacting with parental control and food taboos; media influences reinforcing existing body ideals, and linking these intersections explicitly across socio-ecological levels would make the multi-level analysis more cohesive and impactful. | We have restructured the results and discussion sections to explicitly highlight these intersections across socio-ecological levels. For example, we have explained how seasonality intensified satiety-driven choices, how pocket money interacted with peer norms to encourage unhealthy purchases, how parental control combined with gender norms and food taboos to constrain adolescents’ diets, and how media exposure reinforced body image ideals rooted in cultural norms. These cross-level linkages are now systematically integrated, strengthening the multi-level analysis and demonstrating how overlapping influences shape dietary behaviors. | 247 – 549, 550 - 638 |
